# Safety profile of metformin in adolescents with type 2 diabetes: A pharmacovigilance analysis of the FDA Adverse Event Reporting System

**DOI:** 10.1371/journal.pone.0337204

**Published:** 2025-11-21

**Authors:** Mengsi Peng, Peng Shen, Kyung-In Joung, Kwang Joon Kim

**Affiliations:** 1 College of Pharmacy, Chonnam National University, Gwangju, Republic of Korea; 2 School of Pharmaceutical Science, Wenzhou Medical University, Wenzhou, Zhejiang, China; 3 Department of Rehabilitation Therapy, The Second Affiliated Hospital of Hainan Medical University, Haikou, China; 4 School of AI Healthcare, College of Health Science, CHA University, Pocheon-si, Republic of Korea; UCSI University, MALAYSIA

## Abstract

**Background:**

Although metformin is the first-line medicine for type 2 diabetes (T2D), its safety profile in adolescents remains poorly understood. This study seeks to investigate the adverse events linked to metformin use in adolescents diagnosed with T2D.

**Methods:**

Data from the Food and Drug Administration Adverse Event Reporting System (FAERS), spanning Q1 2004 to Q2 2024, were retrospectively analyzed in this study. Adverse reactions were standardized using the Medical Dictionary for Regulatory Activities, then significant adverse drug reaction signals were identified through disproportionality analysis employing reporting odds ratio (ROR) and information component (IC) methods.

**Results:**

Of 17,956,653 FAERS reports, 80,187 involved metformin, including 973 in adolescents (10–19 years), with 174 cases were identified with a T2D indication. Analysis at the system organ class level revealed that congenital, familial, and genetic disorders [ROR: 8.8 (4.0, 19.3); IC: 2.2 (1.1, 2.9)] and pregnancy conditions [ROR: 4.9 (2.5, 9.5); IC: 1.8 (0.8, 2.5)] showed the most significant signals. At the preferred term (PT) level, three signals were identified across all sexes and subgroups: treatment noncompliance [ROR: overall 4.14 (2.44, 7.02), male 4.27 (2.00, 9.12), and female 4.65 (2.22, 9.74); IC: overall 1.67 (0.88, 2.22), male 1.60 (0.46, 2.36), and female 1.74 (0.60, 2.50)], lactic acidosis [IC: overall 2.99 (1.91, 3.72), male 2.53 (0.76, 3.61), and female 2.76 (1.34, 3.67)], and gastrointestinal disorder [ROR: overall 13.09 (4.73, 36.23), male 54.33 (6.05, 487.96), female 5.34 (1.10, 25.84)]. Neurological disorders were observed only in males, while pregnancy-related adverse effects and renal disorders occurred exclusively in females. Additionally, the study identified potential new signals not documented in metformin labeling, including areflexia, muscle weakness, ataxia, decreased vibratory sense, rhabdomyolysis, substance use, and axillary pain.

**Conclusion:**

The study reveals a complex safety profile of metformin in adolescents with T2D, warranting further research to confirm risks.

## Introduction

Adolescent-onset type 2 diabetes (T2D), as a diagnosis occurring between ages 10 and 19 according to the World Health Organization [1], is a significant chronic condition affecting individuals <20 years [2]. Globally, the incidence of T2D in adolescents has increased significantly in recent decades. In certain racial groups, the incidence rate of T2D among adolescents is twice that of T1D [2,3]. Projections indicate that between 2010 and 2050, the global prevalence of T2D in individuals <20 years is expected to nearly double [4]. In adolescents, T2D often develops gradually and is clinically challenging due to pronounced pubertal insulin resistance, which reduces the effectiveness of oral hypoglycemic agents [5,6]. Compared to that of the adult-onset T2D, the adolescent form progresses more rapidly and tends to be more aggressive, leading to earlier onset of microvascular complications and a higher risk of metabolic comorbidities [7,8]. Impaired glucose regulation during adolescence may also interfere with neurodevelopment, potentially affecting cognitive and behavioral outcomes. Without timely and effective intervention, T2D and its associated complications can significantly increase the risk of premature mortality in affected youth.

Current clinical guidelines consistently recommend metformin, alongside lifestyle modification, as the first-choice intervention in managing T2D among adolescents [9,10]. Data from the Diabetes Alliance cohort indicate that over 60% of adolescents with T2D received metformin-based regimens [11]. Metformin monotherapy, combined with standard diabetes education, achieves glycated hemoglobin (HbA1c) levels < 8.0% in most newly diagnosed adolescents, with sustained efficacy observed in approximately 50% of cases over 12–24 months [12]. Several new drugs, which have received approval from the Food and Drug Administration (FDA), are accessible for use in this population. However, they are typically administered in combination with metformin in clinical practice to improve efficacy and safety profiles [13,14]. Among adolescents with T2D, metformin use has been linked to a lower risk of all-cause hospitalization compared to insulin or sulfonylurea treatments [15].

Despite its range of use, metformin is associated with adverse effects that may compromise patient adherence and disrupt treatment continuity. Most available pharmacovigilance data are derived from adult or mixed-age populations [16–18], with limited evidence specific to adolescents. Adolescents are particularly sensitive to drug safety concerns owing to the immaturity of hepatic and renal functions, alongside differences in drug metabolism and tolerance compared to those of adults [19]. In addition, poor adherence and limited self-monitoring skills among adolescents can lead to delays in recognizing and addressing adverse reactions [20]. These factors underscore the urgent need for youth-specific pharmacovigilance of metformin.

The FDA Adverse Event Reporting System (FAERS) plays a crucial role in safety monitoring after marketing, functioning as a centralized repository for spontaneous reports of adverse drug reactions (ADRs) reported by healthcare workers, patients, and pharmaceutical manufacturers. It facilitates pharmacovigilance by supporting disproportionality analyses, which assess how strongly specific drugs are associated with reported ADRs, aiding in the identification of potential safety signals. This study aims to analyze the FAERS data to comprehensively assess the possible ADR profile of metformin in adolescents with T2D and to investigate potential sex-specific differences in ADRs.

## Materials and methods

### Data source

This study conducted the analysis based on FAERS Quarterly Data Extract Files obtained via the FDA website [21]. The study period was determined on the basis of the availability of relevant drug data. At the time of analysis, the FDA had published data only up to the second quarter of 2024. Given that metformin was approved by the FDA in December 2000, and that the FAERS database contains reports starting from Q1 2004, our analysis included all FAERS reports from the first quarter of 2004 through the second quarter of 2024.

### Definition of cases and drugs of interest

ADR data were standardized using the Preferred Term (PT) classification from the Medical Dictionary for Regulatory Activities (MedDRA), while the System Organ Class (SOC) classification was applied to categorize ADR by physiological systems. Metformin was designated as the primary drug of interest (S1 File). All cases in which metformin was used and classified as the “primary suspect (PS)” drug were included in the analysis.

### Data processing procedure

**Fig 1:**
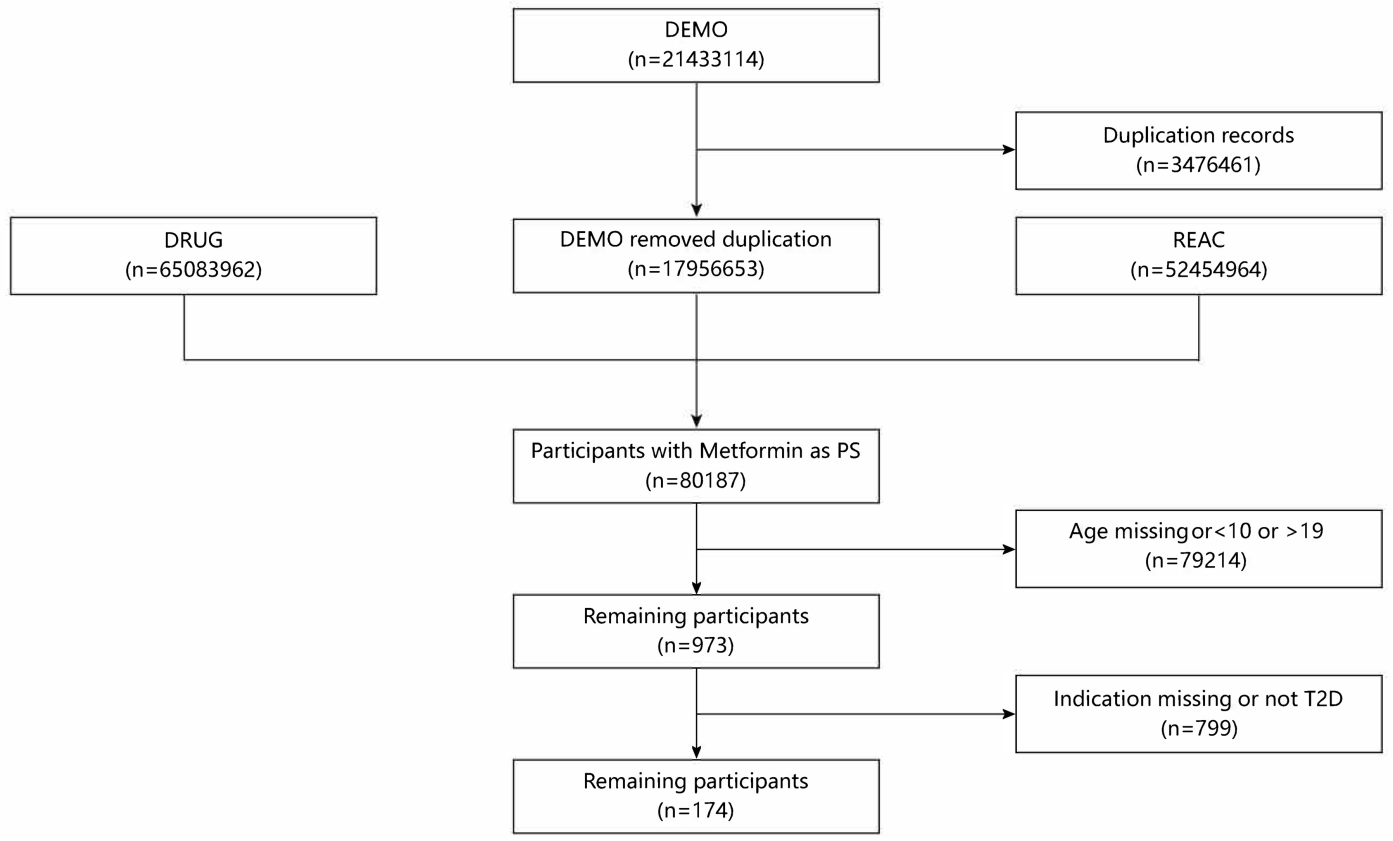
Flowchart of case selection process from the FAERS database. The FAERS database consists of several data files, among which DEMO (demographic), DRUG (drug), and REAC (reaction) were included in this study. Abbreviations: FAERS, Food and Drug Administration Adverse Event Reporting System;PS, primary suspect; T2D, type 2 diabetes.

The FAERS database comprises seven distinct datasets, each serving a specific purpose. The DEMO dataset contains patient demographic information. The DRUG dataset provides details regarding the drugs in the reports. The INDI dataset specifies the medical indications for which the drugs were prescribed. The OUTC dataset records patient outcomes following adverse events. The REAC dataset lists adverse events coded using MedDRA terminology. The PRSR dataset identifies the sources of each report related to the events. The THER dataset records the initiation and termination dates of the therapies associated with the reported drugs. All datasets are interlinked through a primary identifier. Additionally, cases withdrawn by the FDA or manufacturers—owing to reasons such as case consolidation—are documented in the Deleted files. To remove duplicate records, the entry with the latest report date (FDA_DT) was retained when multiple reports used the same case identifier (CASEID). If CASEID and FDA_DT were also the same, the record that had the highest PRIMARY_ID was selected, following FAERS user guidance [22]. Fig 1 presents the data processing procedure.

### Statistics

#### Descriptive analysis.

Reports involving adolescents treated with metformin included demographic information (age, sex, and weight) and clinical characteristics.

#### Disproportionality analysis.

To identify potential ADRs in adolescents with T2D, disproportionality analysis was conducted through the application of the reporting odds ratio (ROR) and information component (IC). ROR is a frequency-based measure used to assess the association between a specific drug and an adverse drug reaction, and is particularly sensitive for ADR signal detection. IC is a Bayesian-based measure that evaluates the deviation between observed and expected counts, providing more robust results for sparse or low-report data. Using both metrics together balances sensitivity and specificity, reducing potential biases from a single method and improving the reliability of signal identification [23,24]. To improve result robustness, a statistical shrinkage transformation was applied. The relative statistical formula is presented below:


$$ROR=\frac{a/b}{c/d}$$

$$\;95\%CI=e^{\ln\left(ROR\right)\pm 1.96\sqrt{\frac{1}{a}+\frac{1}{b}+\frac{1}{c}+\frac{1}{d}}}$$

$$IC=\log_{2}[(a+0.5)/(Nexpected+0.5)]$$

Nexpected=[(a+b)*(a+c)]/(a+b+c+d)

The formulas used to calculate signal strength are as follows [25]: a refers to the number of reports involving both the target drug and the specific adverse event; b represents reports of other adverse events linked to the target drug; c denotes reports of the same adverse event associated with other drugs; and d indicates reports concerning other drugs and other adverse events.

In pharmacovigilance, disproportionality analysis is a key approach for assessing possible associations between specific drugs and adverse events. A signal is considered significant when the lower bound of the 95% confidence interval (CI) for the ROR (ROR₀₂₅) exceeds 1, or when the lower bound of the 95% CI for the IC (IC₀₂₅) is greater than 0. The presence of such signals suggests a potential link between a drug and an adverse reaction.

Beyond standard signal detection at the PT level, adverse events of clinical interest were also analyzed at the SOC levels. To improve the robustness of the findings, separate disproportionality analyses were performed based on patient sex.

### Ethics approval

Patient information in the FAERS database is both anonymized and de-identified, and the database is publicly available. Consequently, this study was exempted from ethical clearance and informed consent requirements. The exemption was granted by the Institutional Review Board of Chonnam National University (IRB No. 1040198–250108-HR-008–01).

## Results

### Descriptive analysis

From the Q1 of 2004 to the Q2 of 2024, 21,433,114 cases were the recorded in FAERS. After removing 3,476,461 duplicate entries, 17,956,653 unique reports were left for analysis (Fig 1). Among these, 80,187 reports involved metformin use. Of this subset, 973 cases were reported in patients aged 10–19 years, while 79,214 involved individuals outside this age range. Finally, 174 adolescent cases with a reported indication of T2D were included for further analysis. Table 1 presents the characteristics of adolescent patients with T2D undergoing metformin treatment.

**Table 1:** Characteristics of adolescents with T2D cases reporting ADRs to metformin in FAERS.

Characteristics	Number	Percentage
Sex		
Female	76	43.70
Male	81	46.60
Missing	17	9.80
Age (years)		
10 ~ 15	64	36.80
16 ~ 19	110	63.20
Weight (kg)		
< 50	8	4.60
50 ~ 100	56	32.20
> 100	10	5.70
Missing	100	57.50
Reporter		
Consumer	20	11.50
Pharmacist	69	39.70
Physician	44	25.30
Other health-professional	29	16.70
Pharmacist	8	4.60
Missing	4	2.30
Country		
United States	57	41.00
Germany	28	20.10
United Kingdom	12	8.60
Saudi Arabia	10	7.20
Others	29	20.90
Missing	3	2.20

Abbreviations: ADRs, Adverse drug reactions; T2D, type 2 diabetes; FAERS, Food and Drug Administration Adverse Event Reporting System.

### Disproportionality analysis

**Fig 2:**
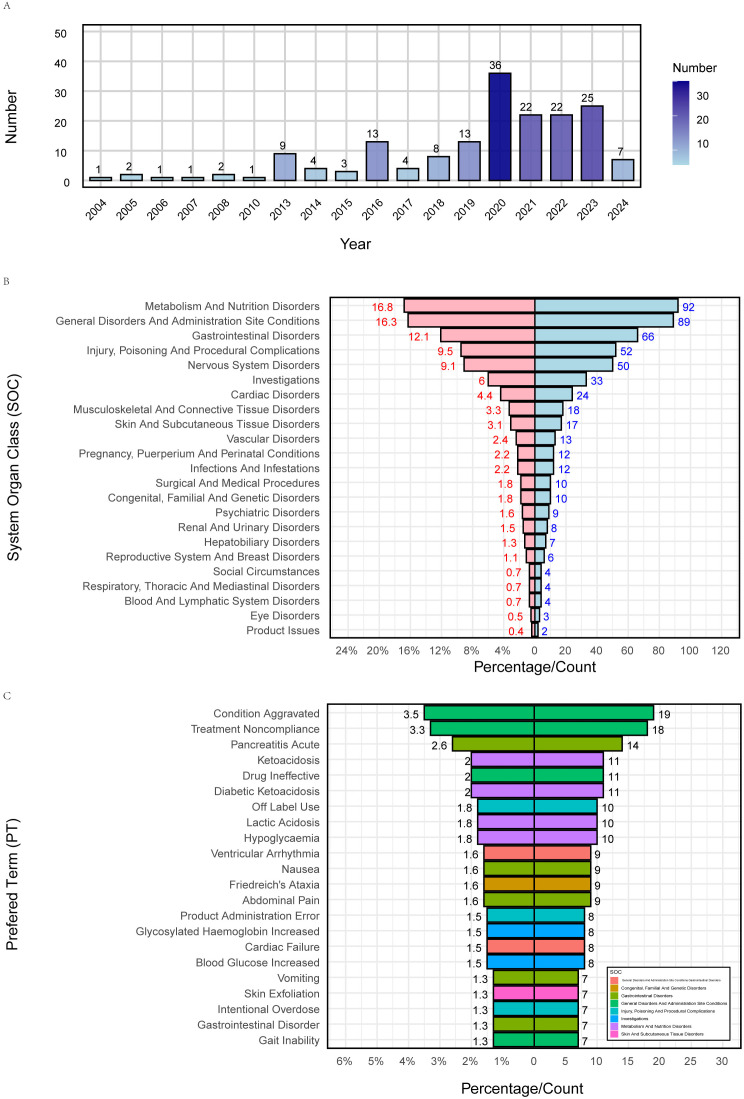
Distribution of cases and adverse events associated with metformin. **(A)** Number of cases by report year; **(B)** Adverse events at SOC Level; **(C)** Top 20 adverse events at PT Level.

Fig 2 shows the distribution of metformin-related cases and associated adverse events. Fig 2A presents the annual number of reported cases. The first adolescent case was reported in 2004, with the highest annual count observed in 2020 (n = 36). Fig 2B and 2C illustrate the number and proportion of adverse events reported at the SOC and PT levels, respectively. At the SOC level, the three most commonly reported adverse event categories were metabolism and nutrition disorders (n = 92, 16.8%), general disorders and administration site conditions (n = 89, 16.3%), and gastrointestinal (GI) disorders (n = 66, 12.1%). At the PT level, the three most frequently reported specific adverse events were condition aggravated (n = 19, 3.5%), treatment noncompliance (n = 18, 3.3%), and pancreatitis acute (n = 14, 2.6%).

**Fig 3:**
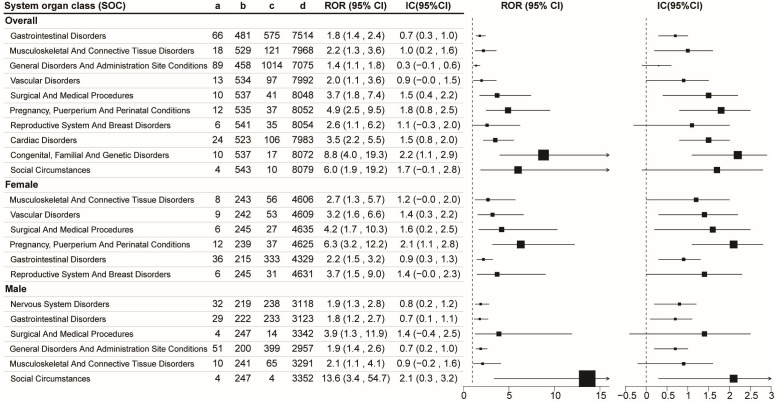
Signals detection based on sex and subgroup at the SOC level utilizing two disproportionality analysis methods. Abbreviations: ROR, Reporting Odds Ratio; IC, Information Component; CI, Confidence Interval.

At the SOC level, the ROR method identified 10 positive signals, while the IC method detected six (Fig 3). The strongest signal was observed for Congenital, familial, and genetic disorders [ROR: 8.8 (4.0, 19.3); IC: 2.2 (1.1, 2.9)], followed by pregnancy, puerperium, and perinatal conditions [ROR: 4.9 (2.5, 9.5); IC: 1.8 (0.8, 2.5)], and surgical and medical procedures [ROR: 3.7 (1.8, 7.4); IC: 1.5 (0.4, 2.2)].

When stratified by sex at the SOC level, six positive signals were observed in adolescents of both sexes using the ROR method, while four signals were identified using the IC method. In males, the strongest signal was observed for social circumstances [ROR: 13.6 (3.4, 54.7); IC: 2.1 (0.3, 3.2)]. In females, the most prominent signal was for pregnancy, puerperium, and perinatal conditions [ROR: 6.3 (3.2, 12.2); IC: 2.1 (1.1, 2.8)]. Additionally, signals for social circumstances [ROR: 13.6 (3.4, 54.7); IC: 2.1(0.3, 3.2)] and nervous system disorders [ROR: 1.9 (1.3, 2.8); IC: 0.8 (0.2, 1.2)] were observed exclusively in males. In contrast, signals for vascular disorder [ROR: 3.2 (1.6, 6.6); IC: 1.4 (0.3, 2.2)], pregnancy, puerperium, and perinatal conditions [ROR: 6.3 (3.2, 12.2); IC: 2.1 (1.1, 2.8)], and reproductive system and rreast disorders [ROR: 3.7 (1.5, 9.0)] were identified only in females.

Several ADRs identified at the SOC level—including general disorders and administration site conditions, pregnancy, puerperium, and perinatal conditions, congenital, familial, and genetic disorders, social circumstances, and nervous system disorders—were not included in the official drug labeling for metformin.

At the PT level, the ROR analysis identified two significant signals—treatment noncompliance [ROR: overall, 4.14 (2.44, 7.02); male: 4.27 (2.00, 9.12); female: 4.65 (2.22, 9.74)] and gastrointestinal disorder [ROR: overall, 13.09 (4.73, 36.23); male: 54.33 (6.05, 487.96); female: 5.34 (1.10, 25.84)]—across the overall population and sex-specific subgroups (Table 2). Table 2 presents the results of the IC analysis, which identified two significant signals across all sex and subgroups: lactic acidosis, with IC values of overall: 2.99 (1.91, 3.72), male: 2.53 (0.76, 3.61), and female: 2.76 (1.34, 3.67); and treatment noncompliance, with IC values of overall: 1.67 (0.88, 2.22), male: 1.60 (0.46, 2.36), and female: 1.74 (0.60, 2.50).

**Table 2:** Signal detection by sex and subgroup at the PT level.

	Preferred Term	Overall			Male			Female		
		N	ROR (95% CI)	IC (95% CI)	N	ROR (95% CI)	IC (95% CI)	N	ROR (95% CI)	IC (95% CI)
1	Treatment Noncompliance	18	4.14 (2.44, 7.02)	1.67 (0.88, 2.22)	9	4.27 (2.00, 9.12)	1.60 (0.46, 2.36)	9	4.65 (2.22, 9.74)	1.74 (0.60, 2.50)
2	Lactic Acidosis	10	50.19 (13.77, 182.91)	2.99 (1.91, 3.72)	4	NA	2.53 (0.76, 3.61)	6	38.03 (9.45, 152.97)	2.76 (1.34, 3.67)
3	Gastrointestinal Disorder^#^	7	13.09 (4.73, 36.23)	2.37 (1.07, 3.22)	4	54.33 (6.05, 487.96)	2.41 (0.64, 3.49)	2	5.34 (1.10, 25.84)	1.38 (−1.21, 2.77)
4	Intentional Overdose	7	4.18 (1.80, 9.71)	1.57 (0.27, 2.42)	1	3.35 (0.37, 30.09)	0.82 (−2.96, 2.51)	6	5.68 (2.26, 14.27)	1.83 (0.41, 2.74)
5	Product Administration Error	8	3.06 (1.42, 6.58)	1.29 (0.08, 2.09)	0	NA	NA	8	8.49 (3.66, 19.72)	2.22 (1.01, 3.02)
6	Medication Error	5	3.72 (1.39, 9.95)	1.40 (−0.16, 2.38)	5	6.80 (2.31, 20.05)	1.83 (0.27, 2.81)	0	NA	NA
7	Drug Abuse	4	29.79 (5.44, 163.01)	2.35 (0.58, 3.43)	4	27.16 (4.95, 149.02)	2.29 (0.52, 3.37)	0	NA	NA
8	Product Prescribing Error	3	22.30 (3.72, 133.74)	2.10 (0.03, 3.31)	3	20.29 (3.37, 122.00)	2.05 (−0.02, 3.26)	0	NA	NA
9	Toxicity To Various Agents	5	74.61 (8.70, 639.77)	2.64 (1.08, 3.62)	4	NA	2.53 (0.76, 3.61)	1	18.64 (1.16, 298.89)	1.32 (−2.46, 3.01)
10	Substance Use	4	NA	2.58 (0.81, 3.66)	4	NA	2.53 (0.76, 3.61)	0	NA	NA
11	Off-Label Use	10	1.49 (0.77, 2.87)	0.49 (−0.59, 1.22)	7	2.72 (1.20, 6.19)	1.13 (−0.17, 1.98)	3	0.88 (0.27, 2.82)	−0.15 (−2.22, 1.06)
12	Overdose	5	2.75 (1.05, 7.17)	1.12 (−0.44, 2.10)	2	2.69 (0.59, 12.34)	0.91 (−1.68, 2.30)	3	3.31 (0.96, 11.37)	1.20 (−0.87, 2.41)
13	Elevated Glycosylated Hemoglobin	8	1.68 (0.80, 3.51)	0.63 (−0.58, 1.43)	8	3.42 (1.56, 7.50)	1.37 (0.16, 2.17)	0	NA	NA
14	Metabolic Disorder	5	10.65 (3.37, 33.67)	2.13 (0.57, 3.11)	5	34.09 (6.58, 176.61)	2.48 (0.92, 3.46)	0	NA	NA
15	Ketoacidosis	11	1.89 (1.00, 3.56)	0.78 (−0.24, 1.47)	7	3.41 (1.47, 7.89)	1.35 (0.05, 2.20)	4	1.29 (0.46, 3.58)	0.30 (−1.47, 1.38)
16	Metabolic Acidosis	6	4.47 (1.79, 11.18)	1.60 (0.18, 2.51)	1	6.71 (0.61, 74.26)	1.08 (−2.70, 2.77)	5	5.24 (1.93, 14.23)	1.72 (0.16, 2.70)
17	Hyperkalemia	5	18.65 (4.99, 69.65)	2.36 (0.80, 3.34)	4	18.10 (4.03, 81.33)	2.19 (0.42, 3.27)	1	18.64 (1.16, 298.89)	1.32 (−2.46, 3.01)
18	Hepatic Steatosis	3	8.92 (2.13, 37.42)	1.80 (−0.27, 3.01)	0	NA	NA	3	28.19 (4.69, 169.48)	2.21 (0.14, 3.42)
19	Pancreatitis Acute	14	8.15 (4.23, 15.70)	2.26 (1.36, 2.88)	13	30.50 (11.49, 80.96)	2.89 (1.95, 3.53)	1	0.93 (0.12, 6.96)	−0.07 (−3.85, 1.62)
20	Abdominal Pain	9	2.28 (1.12, 4.62)	0.98 (−0.16, 1.74)	2	1.49 (0.34, 6.46)	0.40 (−2.19, 1.79)	7	3.40 (1.51, 7.68)	1.40 (0.10, 2.25)
21	Abdominal Distension	4	5.41 (1.72, 17.05)	1.63 (−0.14, 2.71)	0	NA	NA	4	9.42 (2.82, 31.50)	2.02 (0.25, 3.10)
22	Nausea	9	1.28 (0.64, 2.54)	0.31 (−0.83, 1.07)	1	0.34 (0.05, 2.49)	−1.13 (−4.91, 0.56)	8	2.40 (1.14, 5.06)	1.04 (−0.17, 1.84)
23	Caesarean Section	3	6.37 (1.64, 24.70)	1.63 (−0.44, 2.84)	0	NA	NA	3	8.04 (2.07, 31.28)	1.79 (−0.28, 3.00)
24	Pregnancy	3	NA	2.34 (0.27, 3.55)	0	NA	NA	3	NA	2.42 (0.35, 3.63)
25	Presyncope	4	29.79 (5.44, 163.01)	2.35 (0.58, 3.43)	0	NA	NA	4	75.48 (8.40, 677.86)	2.57 (0.80, 3.65)
26	Dizziness	6	1.98 (0.84, 4.66)	0.80 (−0.62, 1.71)	0	NA	NA	6	4.37 (1.78, 10.72)	1.61 (0.19, 2.52)
27	Areflexia	4	59.58 (6.65, 534.00)	2.46 (0.69, 3.54)	4	54.33 (6.05, 487.96)	2.41 (0.64, 3.49)	0	NA	NA
28	Muscular Weakness	4	8.51 (2.48, 29.16)	1.91 (0.14, 2.99)	4	27.16 (4.95, 149.02)	2.29 (0.52, 3.37)	0	NA	NA
29	Ataxia	3	14.86 (2.99, 73.80)	1.99 (−0.08, 3.20)	3	13.52 (2.71, 67.34)	1.93 (−0.14, 3.14)	0	NA	NA
30	Friedreich’s Ataxia^#^	9	NA	3.15 (2.01, 3.91)	1	NA	1.40 (−2.38, 3.09)	NA	NA	NA
31	Decreased Vibratory Sense	4	NA	2.58 (0.81, 3.66)	4	NA	2.53 (0.76, 3.61)	0	NA	NA
32	Gait Inability	7	NA	2.99 (1.69, 3.84)	7	NA	2.93 (1.63, 3.78)	0	NA	NA
33	Vitamin B12 Deficiency Neuropathy	6	NA	2.88 (1.46, 3.79)	6	NA	2.82 (1.40, 3.73)	0	NA	NA
34	Vitamin B12 Deficiency	3	NA	2.34 (0.27, 3.55)	3	NA	2.30 (0.23, 3.51)	0	NA	NA
35	Cardiac Arrest	3	8.92 (2.13, 37.42)	1.80 (−0.27, 3.01)	3	13.52 (2.71, 67.34)	1.93 (−0.14, 3.14)	0	NA	NA
36	Swelling	4	11.91 (3.19, 44.48)	2.07 (0.30, 3.15)	4	27.16 (4.95, 149.02)	2.29 (0.52, 3.37)	0	NA	NA
37	Hypertension	5	3.72 (1.39, 9.95)	1.40 (−0.16, 2.38)	2	2.99 (0.64, 13.91)	0.98 (−1.61, 2.37)	3	5.11 (1.42, 18.43)	1.53 (−0.54, 2.74)
38	Cardiac Failure^#^	8	40.00 (10.58, 151.21)	2.83 (1.62, 3.63)	1	6.71 (0.61, 74.26)	1.08 (−2.70, 2.77)	NA	NA	NA
39	Condition Aggravated^#^	19	13.19 (7.09, 24.52)	2.65 (1.88, 3.19)	10	23.17 (8.35, 64.29)	2.70 (1.62, 3.43)	1	1.16 (0.15, 8.78)	0.13 (−3.65, 1.82)
40	Suicide Attempt	3	2.34 (0.69, 7.93)	0.89 (−1.18, 2.10)	0	NA	NA	3	4.02 (1.15, 14.08)	1.35 (−0.72, 2.56)
41	Multiple Organ Dysfunction Syndrome	3	44.60 (4.63, 429.50)	2.22 (0.15, 3.43)	3	NA	2.30 (0.23, 3.51)	0	NA	NA
42	Ventricular Arrhythmia	9	135.30 (17.11, 1069.94)	3.07 (1.93, 3.83)	1	13.42 (0.84, 215.20)	1.23 (−2.55, 2.92)	NA	NA	NA
43	Acute Kidney Injury	3	2.12 (0.63, 7.13)	0.79 (−1.28, 2.00)	0	NA	NA	3	3.75 (1.08, 13.04)	1.30 (−0.77, 2.51)
44	Nephropathy	4	NA	2.58 (0.81, 3.66)	1	NA	1.40 (−2.38, 3.09)	3	NA	2.42 (0.35, 3.63)
45	Elevated Blood Creatinine	5	24.86 (5.93, 104.30)	2.45 (0.89, 3.43)	1	4.47 (0.46, 43.13)	0.95 (−2.83, 2.64)	4	NA	2.68 (0.91, 3.76)
46	Rhabdomyolysis	5	10.65 (3.37, 33.67)	2.13 (0.57, 3.11)	4	27.16 (4.95, 149.02)	2.29 (0.52, 3.37)	1	3.73 (0.43, 32.05)	0.90 (−2.88, 2.59)
47	Back Pain	3	5.57 (1.47, 21.06)	1.55 (−0.52, 2.76)	0	NA	NA	3	9.39 (2.33, 37.77)	1.87 (−0.20, 3.08)
48	Axillary Pain	4	29.79 (5.44, 163.01)	2.35 (0.58, 3.43)	4	27.16 (4.95, 149.02)	2.29 (0.52, 3.37)	0	NA	NA
49	Decreased Exercise Tolerance	4	NA	2.58 (0.81, 3.66)	4	NA	2.53 (0.76, 3.61)	0	NA	NA
50	Subcutaneous Abscess	4	11.91 (3.19, 44.48)	2.07 (0.30, 3.15)	4	27.16 (4.95, 149.02)	2.29 (0.52, 3.37)	0	NA	NA
51	Skin Exfoliation^#^	7	20.96 (6.63, 66.26)	2.57 (1.27, 3.42)	NA	NA	NA	NA	NA	NA

#Total cases may not equal the sum of males and females due to missing data.

NA for N: Cases count unavailable due to missing data.

NA for ROR or IC: Not applicable when either b (number of reports with other adverse events for the target drug) or c (number of reports with the target adverse event for other drugs) is zero.

Abbreviations: CI, confidence interval; ROR, reporting odds ratio; IC, information component.

Further analysis revealed that neurological-associated disorders including, areflexia [ROR: 54.33 (6.05, 487.96), IC: 2.41 (0.64, 3.49)], muscle weakness [ROR: 27.16 (4.95, 149.02), IC: 2.29 (0.52, 3.37)], ataxia [ROR: 13.52 (2.71, 67.34)], decreased vibratory sense [IC: 2.53 (0.76, 3.61)], gait inability [IC: 2.93 (1.63, 3.78)], and vitamin B12 deficiency neuropathy [IC: 2.82 (1.40, 3.73)], were reported only in males. In contrast, pregnancy-related ADRs such as pregnancy [IC: 2.42 (0.35, 3.63)] and caesarean section [ROR: 8.04 (2.07, 31.28)], and renal diseases, including acute kidney injury [ROR: 3.75 (1.08, 13.04)], nephropathy [IC: 2.42 (0.35, 3.63)], and elevated blood creatinine [IC: 2.68 (0.91, 3.76)], occurred exclusively in females.

**Fig 4:**
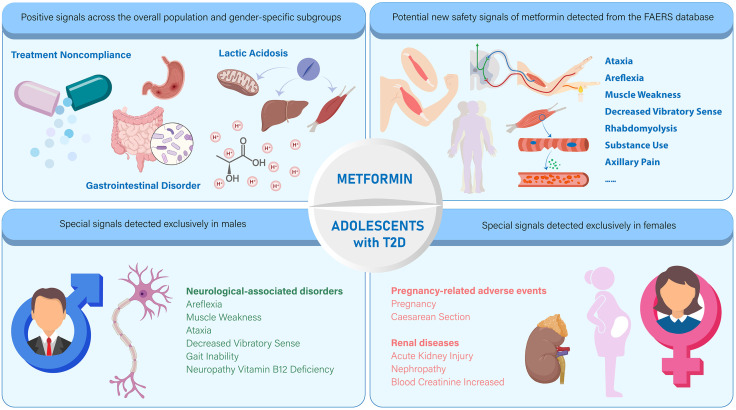
Visual summary of metformin-associated ADRs in adolescents with T2D. Abbreviations: ADRs, adverse drug reactions; T2D, type 2 diabetes.

To facilitate interpretation of the identified adverse event signals, including sex-specific and potentially novel associations, a summary visualization is presented in Fig 4.

## Discussion

This study is the first pharmacovigilance analysis of metformin-related ADRs in adolescents with T2D, highlighting unique challenges in this population. At the PT level, both the overall cohort and subgroups showed significant disproportionality signals for treatment noncompliance, lactic acidosis and gastrointestinal disorder. Notably, sex-specific differences emerged: males were more likely to develop neurological disorders, with severe cases involving condition aggravated and multiple organ dysfunction syndrome (MODS); while adolescent females showed higher reporting of pregnancy-related ADRs and renal diseases, and in severe cases, they may report suicide attempt.

### Common signals for all populations

#### Treatment noncompliance.

Adolescents differ from adults in pharmacokinetic profiles and self-management, resulting in unique metformin-related ADRs. While GI disturbances, anemia, and lactic acidosis are common ADRs in adults with T2D [26], adolescents face significant challenges with treatment noncompliance. One study attributed metformin failure in 62% of adolescent cases to noncompliance rather than pharmacologic inefficacy [27]. In contrast, metformin-related ADRs in adults rarely disrupt treatment continuity [28]. In this study, treatment noncompliance was the second most frequently reported ADR and demonstrated consistent signal detection across multiple analytical methods. This finding is clinically significant, as serious ADRs such as elevated glycated hemoglobin, disease exacerbations, and acute pancreatitis may result from treatment noncompliance. However, the observational nature of spontaneous reporting systems limits causal inference, requiring caution when interpreting associations between treatment noncompliance and other ADRs. It remains unclear whether treatment noncompliance leads to disease progression and complications (thereby increasing ADR reporting) or if ADRs contribute to noncompliant behaviors. Among medication-related problems, sex differences were observed: in females, intentional overdose and product administration errors showed significant signals, while in males, signals were detected for medication errors, substance abuse, off-label use, and product prescription errors.

#### Lactic acidosis.

At the PT level, lactic acidosis consistently emerged as a positive signal across all subgroups. It is also listed as a black box warning by the FDA. This life-threatening complication results from multiple pathophysiological pathways, including metformin overdose, cardiac decompensation, and organ dysfunction [29]. Lactic acidosis often presents insidiously and may be masked by other complications. Early recognition and prompt intervention are critical, as delayed treatment can lead to renal failure, cardiac arrest, and other severe outcomes. [30]. While another pharmacovigilance study identified lactic acidosis as the strongest disproportionality signal for metformin [18], this study ranked it third (IC method). This discrepancy highlights the need for population-specific monitoring in adolescents, who may struggle more than adults to recognize and report lactic acidosis in its asymptomatic early stages. Notably, ketoacidosis and metabolic acidosis showed positive signals in males and females, respectively. Metabolic acidosis includes both ketoacidosis and lactic acidosis. This pattern likely reflects reporting bias rather than true biological sex differences, as reporters may vary in how precisely they describe acidosis, influencing adverse event coding. Clinically, accurate differentiation of acidosis subtypes is essential for precision treatment strategies.

#### Gastrointestinal disorder.

GI disturbances are consistently identified as the predominant ADRs associated with metformin [31]. A previous pharmacovigilance analysis of metformin also ranked GI disorders as the second most reported ADRs [18]. A meta-analysis indicated that adults with T2D receiving metformin had a 50% higher risk of abdominal pain compared to controls, with no additional risk when another hypoglycemic drug was added [16]. Notably, adolescents with T2D exhibit distinct GI tolerance profiles. A network meta-analysis showed that metformin monotherapy did not significantly increase GI disorders compared to placebo in adolescents, though the combination therapy with liraglutide may increase the risk of abdominal pain [13]. Moreover, GI disorders such as nausea and diarrhea in adolescents with T2D typically do not worsen with long-term metformin use [32]. In our study, GI disorders ranked as the third most frequently reported ADR signal at the SOC level. This contrasts with the TODAY study, which identified GI disorders as the most common metformin-related ADR in youth [26]. At the PT level, metformin was significantly linked to GI disturbances in males, while nausea, abdominal distension, and pain were the primary GI signals in females. GI severity is much lower in adolescents than in adults with T2D. This may be attributed to the frequent co-administration of metformin with other hypoglycemic drugs, where lower metformin doses or complementary pharmacodynamics reduce the risk of GI discomfort. Additionally, poor adherence among adolescents, including intermittent skipping of doses, may reduce exposure to metformin-associated GI disorders. Furthermore, the spontaneous reporting nature of FAERS likely underrepresents mild-to-moderate ADRs (such as GI disorder), as severe events are more frequently reported.

### Differences between sexes

Sex-specific analysis is essential in pharmacovigilance for adolescents with T2D, as biological and behavioral differences between sexes significantly affect drug safety profiles. Adolescents exhibit pronounced sex-specific differences in endocrine profiles, including sex hormones, insulin sensitivity, and adiponectin levels [33]. These variations influence glucose metabolism and the degree of insulin resistance, thereby modulating both the efficacy and the adverse effect profile of antidiabetic therapies. In the case of metformin, its distribution and elimination depend on a range of organic cation transporters, whose expression and activity can be regulated by sex hormones and are subject to genetic polymorphisms [34]. Accordingly, the marked endocrine and pharmacokinetic differences between male and female adolescents are closely linked to the observed sex-specific signals of metformin-related adverse reactions. Epidemiological data show that, compared to males, female adolescents exhibit a greater prevalence of prediabetes and T2D, likely attributed to greater insulin resistance and visceral adiposity [10,35,36]. Women with early-onset T2D are at elevated risk of negative pregnancy outcomes such as preterm delivery and fetal macrosomia [37], and are more likely to develop polycystic ovary syndrome (PCOS) during reproductive age (15 years and older) [38]. These findings are consistent with our study, which identified positive signals for pregnancy and caesarean section in females —likely attributable to T2D itself rather than metformin. Notably, metformin exerts pleiotropic endocrine effects, including delayed menarche, normalized menstrual cycles, and reduced androgen/lipid levels, all relevant to PCOS management [39]. The TODAY trial reported 65 pregnancies among youth with T2D over 2–6.5 years of follow-up, including six cases of congenital anomalies (9.2% prevalence) attributed to maternal metformin discontinuation and disease decompensation [26]. We speculate that the pregnancy and caesarean section signals observed in our study may reflect the protective role of metformin during pregnancy in adolescent females with T2D. Unintentional treatment cessation may lead to fetal malformations, prompting early caesarean delivery. Prior studies also suggested that metformin confers renal protection by significantly reducing the risk of chronic kidney disease (CKD) in treatment-naive patients with T2D without preexisting CKD [40]. In our study, renal-related signals, including acute kidney injury, nephropathy, and elevated blood creatinine, were observed exclusively in females. This pattern may be related to faster progression of T2D in females, along with treatment noncompliance and higher drug resistance [41]. Our results highlight the importance of ensuring adherence to prescribed therapy in women with T2D during pregnancy to prevent adverse maternal-fetal outcomes and renal complications. Evidence indicates that metformin can impair vitamin B12 absorption in intestinal cells, potentially causing or exacerbating neurological disorders [42]. While the approved metformin label lists vitamin B12 deficiency as an adverse reaction, our analysis identified previously unreported neurological signals, including areflexia, muscle weakness, ataxia, decreased vibratory sense, gait inability, and decreased exercise tolerance, all statistically significant in males. Routine monitoring and supplementation of vitamin B12 are recommended for male adolescents with T2D undergoing long-term metformin therapy to support neurological health [43].

Pharmacovigilance analysis reveals that, compared to females (ROR: 16/42; IC: 13/41), males (ROR: 20/42; IC: 25/41) exhibit more positive safety signals associated with metformin use. For severe ADR, males are more frequently associated with “condition aggravated” and MODS, while females show positive signals for “suicide attempt”. These significant sex-based differences may be influenced by sociocultural factors. Compared to males, female patients with T2D typically adopt more proactive health behaviors, including stricter adherence to dietary modifications, exercise routines, and weight management strategies. And females tend to have more frequent interactions with healthcare professionals and adjust treatment plans more promptly, contributing to improved glycemic control and lower incidence/severity of metformin-associated ADRs [44,45]. Men often underestimate the effect of illness on their daily lives and tend to wait passively for symptoms to resolve rather than actively seeking medical attention. This behavior may contribute to an elevated risk of ADR and a higher likelihood of disease progression. While metformin may alleviate depressive symptoms by improving insulin sensitivity, abrupt discontinuation in patients with mood disorders can destabilize their mental health. Additionally, adolescent girls are more susceptible to depression, potentially due to hormonal fluctuations and other psychosocial factors. Therefore, mental health surveillance should be performed in adolescents, particularly adolescent girls with preexisting mental health conditions, to prevent suicidal behavior [46]. Furthermore, significant differences exist in the strength and ranking of ADR signals between sexes. Even within the same disease category, the specific types of conditions associated with metformin-related ADRs may differ between males and females. For example, among cardiovascular disorders, males exhibit positive signals for cardiac arrest and peripheral swelling, while females show positive signals for hypertension. These sex-based differences highlight the need for sex-specific pharmacovigilance and targeted interventions to optimize the therapeutic efficacy and safety of metformin.

### Rare adverse drug reactions

Our analysis revealed several rare ADRs not listed on the metformin label, including rhabdomyolysis (n = 5), substance use (n = 4), axillary pain (n = 4), and MODS (n = 3). While the metformin prescribing information recognizes pediatric patients as a special population for monitoring adverse effects, available data remain extremely limited. This scarcity is largely due to the fact that most adolescent patients are minors, and studies in this population are often constrained by recruitment criteria, ethical review requirements, and informed consent procedures. Our analysis revealed ataxia, ventricular arrhythmia, and lactic acidosis as the strongest pharmacovigilance signals, in contrast to findings from the TODAY trial, which reports gastrointestinal disturbances, anemia, and psychiatric events as the leading adverse effects in adolescents [26]. This discrepancy is potentially stem from differences in study design: while clinical trials typically evaluate short-term safety within highly controlled and selectively enrolled populations, spontaneous reporting systems capture the long-term safety of medications across varied real-world populations. Our findings may address gaps in clinical research by highlighting ADRs previously underrepresented or not prominently listed in existing drug labels. Future rigorous clinical trials or large-scale real-world studies may be required to further confirm the incidence, risk factors, and detailed characteristics of these rare ADRs, providing essential evidence to support potential updates to drug labeling and clinical guidelines.

### Clinical implications

Our findings highlight several implications for clinical practice. Treatment noncompliance, lactic acidosis, and gastrointestinal disorders emerged as the most clinically significant ADRs in adolescents with T2D, underscoring the need for adherence support and timely monitoring. Moreover, the observed sex-specific differences—neurological ADRs confined to males and pregnancy-related or renal complications occurring exclusively in females—emphasize the importance of incorporating sex-based considerations into pharmacovigilance and individualized care. Recognition of underreported ADRs further suggests that clinicians should remain vigilant for a broader spectrum of potential harms and adapt monitoring strategies accordingly. It should be noted that the FAERS data used in this study contain missing information, which may limit the generalizability of the findings. Future research could explore ADR specificity more thoroughly by stratifying adolescents according to age, BMI, or country, to better understand factors influencing the occurrence of adverse reactions.

### Limitations and advantages

This study has several limitations. First, as a spontaneous reporting system, the FAERS database has inherent shortcomings such as underreporting, incomplete entries, and data inaccuracies. The missing rate of age entries in the FAERS database is as high as 40% [22], which drastically reduced our effective sample size. Additionally, 57.5% of body weight entries were missing in this study, further limiting the ability to perform detailed subgroup analyses. Second, the majority of data used in this study originated from the United States, and T2D in adolescents varies by race and ethnicity; so, the findings may need to be treated with caution in their application. Third, the cross-sectional nature of FAERS reporting limits causal interpretation, as the lack of temporal data makes it unclear whether medication non-adherence precedes adverse events or occurs as a result of them. This limitation applies to all observed associations, as the database lacks longitudinal data necessary to determine causa directionality or evaluate the sequential progression of ADRs. Fourth, several key confounding variables were not captured in the FAERS database, including disease duration, glycemic control indicators, comorbidity burden, and concomitant medication use. The absence of these variables introduces potential ecological bias, as uncontrolled confounding may distort associations between ADR and metformin use [23].

## Conclusion

This study identified treatment noncompliance, lactic acidosis, and gastrointestinal disorders as the three most clinically significant ADRs in adolescents with T2D, along with sex-specific disparities in neurological, pregnancy-related, and renal complications. In addition, we report several ADRs not described in current product labeling, including areflexia, muscle weakness, ataxia, decreased vibratory sense, rhabdomyolysis, substance use, axillary pain, and MODS. These findings expand the known safety profile of metformin in this population and provide evidence that may inform updates to clinical guidelines and drug labeling. Future large prospective studies are warranted to validate these associations and guide the development of targeted strategies for optimizing metformin safety and efficacy.

## Supporting information

S1 File Search terms for metformin in MedDRA version 27. MedDRA, Medical Dictionary for Regulatory Activities.
(DOCX)

S2 File Original data (a, b, c, d) and disproportionality analysis results (ROR & IC). ROR, Reporting odds ratio; IC, information component.
(XLSX)
