# Supplementary material for: Safety profile of metformin in adolescents with type 2 diabetes: A pharmacovigilance analysis of the FDA Adverse Event Reporting System
Source: PLoS One. 2025 Nov 21;20(11):e0337204. doi: 10.1371/journal.pone.0337204 (PMC12637960; doi:10.1371/journal.pone.0337204)
Supplement: S1 File — MedDRA, Medical Dictionary for Regulatory Activities. (DOCX) [file pone.0337204.s001.docx]

**S1 file. Search terms for metformin in MedDRA version 27**

"METFORMIN HCL", "METFORMIN", "EMTFORMIN HYDROCHLORIDE METFORMIN HYDROCHLORIDE", "METFORMIN HYDROCHLORIDE GLUCOPHAGE SLOW RELEASE", "METFORMINE MERCK", "METFORMINE CHLORHYDRATE", "GLUCOPHAGE RETARD METFORMIN HCL", "METFORMIN EMBONATE METFORMIN EMBONATE", "METFORMIN METFORMIN TABLETS", "STAGID METFORMIN EMBONATE", "METFORMIN IMMEDIATE RELEASE TAB", "GLUCOPHAGE XR METFORMIN HYDROCHLORIDE", "METFORMIN HCL 500", "METFORMIN 850 APOTEX", "APOTEX METFORMIN", "METFORMIN 850MG APOTEX TORPHARM", "METFORMIN TAB", "METFORMIN ER 500 PAR", "DIFORMIN METFORMIN HYDROCHLORIDE TABLETS", "METFORMIN HYDROCHLORIDE", "GLUCOPHAGE ABIC METFORMIN HYDROCHLORIDE", "METFORMIN GLYBURIDE", "METFORMIN EMBONATE STAGID TABLETS", "GLIBOMET METFORMIN HYDROCHLORIDE GLIBENCLAMIDE", "GLUCOPHAGE METFORMIN HYDRCHLORIDE", "METFORMIN XR", "GLUCOPHAGE METFORMIN HYDROCHLORIDE 500 MILLIGRAM", "GLUCOPHAGE ABIC METFORMIN HYDROCHLORIDE", "METFORMIN HYDROCHLORIDE ROSIGLITAZONE MALEATE TABLET METFORMIN HCL", "GLUCOPHAGE METFORMIN HYDROCHLORIDE FILM COATED", "APO METFORMIN METFORMIN HYDROCHLORIDE", "METFORMIN HYDROCHLORIDE GLIBENCLAMIDE GLUCOVANCE", "GLUCOVANCE GLYBURIDE METFORMIN", "GLIPIZIDE METFORMIN", "METFORMIN METFORMIN 1500 MILLIGRANM", "METFORMINE METFORMIN HYDROCHLORIDE", "METFORMIN HCL ER 500MG IVAX", "DIANBEN METFORMIN HYDROCHLORIDE", "METFORMIN ER 500 MG MNF PAR", "METFORMIN HCL METFORMIN HYDROCHLORIDE", "METFORMIN 850 MG APROTEX", "METFORMIN 500 APOTEX", "METFORMIN METFORMIN 500 MILLIGRAM", "METFORMIN HYDROCHLORIDE METFORMIN HCL", "METFORMIN METFORMIN 1500 MILLIGRAM", "GLUCOPHAGE UNS METFORMIN HYDROCHLORIDE", "METFORMIN ER 500 MG IVAX PHARMACEUTICALS INC", "METFORMIN APOTEX ER 500 MG", "GLYBURIDE AND METFORMIN HCL", "MELBIN METFORMIN HYDROCHLORIDE", "METFORMIN HCL METFORMIN HCL", "GLYBURIDE 1 25 MG METFORMIN HCL", "METFORMIN 1000 MG TORPHARM APOTEX", "DIABEX METFORMIN HYDROCHLORIDE TABLETS", "STAGID 800 METFORMIN EMBONATE", "GLUCOPHAGE METFORMIN HYDROCHLORIDE UNSPECIFIED", "METFORMIN EMBONATE", "GLUCOPHAGE METFORMIN HYDROCHLORIDE TABLETS", "DIAFORMIN METFORMIN HYDROCHLORIDE", "METFORMIN 850 MG APOTEX TOEPHARM", "METFORMIN CR 500 MG PER MFR", "GLYBURIDE AND METFORMIN HYDROCHLORIDE", "METFORMIN 500 MG UDL", "GLIBENCLAMIDE METFORMIN HCL GLUCOMIDE", "GLIBENCLAMIDE METFORMIN GLYBURIDE METFORMIN HCL GLIBOMET", "DIABETASE METFORMIN HYDROCHLORIDE", "GLUCOVANCE GLYBURIDE METFORMIN", "DIANBEN METFORMIN HYDROCHLORIDE TABLET 850 MG", "METFORMIN METFORMIN", "METFORMIN TABLETS METFORMIN", "METFORMIN METOFRMIN", "METFORMIN METFORMIN", "GLUCOPHATE METFORMIN HYDROCHLORIDE", "METFORMIN ER 500MG TABS APOTEX", "METFORMIN 850MG TABS TORPHARM APOTEX", "METFORMIN 500MG TABS TORPHARM APOTEX", "METFORMIN ER 500 MG APOTEX TORPHARM", "GLIBOMET GLIBENCLAMIDE METFORMIN HYDROCHLORIDE", "METFORMIN HYDROCHLORIDE EXTENDED RELEASE", "DIANBEN 850 MG TABLETS METFORMIN HDYROCHLORIDE", "METFORMINE METFORMIN", "GLUCOPHAGE 850 MG TABLETS METFORMINH HYDROCHLORIDE", "GLUCOPHAGE METFORMIN HYDROCHLORIDE 850 MILLIGRAM", "METFORMIN METFORMIN TABLET", "METFORMIN METFORMIN NR", "METFORMIN METFORMIN TABLETS", "GLIBENCLAMIDE METFORMIN", "METFORMIN CARACO PHARMACEUTICALS METFORMIN 1000MG", "STAGID METFORMIN", "LOMOTIL METFORMIN HYDROCHLORIDE", "GLUCOPHAGE BRISTOL MYERS SQUIBB METFORMIN", "STAGID METFORMIN EMBONATE", "GLYBURIDE METFORMIN HYDROCHLORIDE", "METFORMIN GENERIC 850 MG", "METFORMIN METFORMIN 0", "GLYBURIDE AND METFORMIN", "METFORMINE METFORMIN", "METFORMIN GLYBURIDE 5 500", "GLYBURIDE METFORMIN 2 5 500 BMS PAR", "METFORMIN 850 MG MGM SANDOZ GENERIC", "GLYBURIDE METFORMIN GLUCOVANCE", "METFORMIN 850MG TABLETS SANDOZ INC", "ORAL HYPOGLYCEMIC AGRNTS METFORMIN GLIBENCLAMIDE", "METFORMIN METFOMRIN", "METFORMIN METFORMIN 500", "GLIBOMENT GLIBENCLAMIDE METFORMIN HYDROCHLORIDE", "ORAL HYPOGLYCEMIC AGENTS METFORMIN GLIBENCLAMIDE", "METFORMIN MANFACTURER IS TEVA", "DIABESIN METFORMIN HYDROCHLORIDE", "GLYBURIDE METFORMIN IPI TABLETS", "GLUCOPHAGE 850 METFORMIN HYDROCHLORIDE", "GLYBURIDE METFORMIN", "GLYBURIDE METFORMIN", "METFORMIN HYDROCHLORIDE GLYBURIDE", "GLIBOMET GLIBENCLAMIDE METFORMIN", "METFORMIN 500 MG TABLET SANDOZ MANUFACTURER", "METFORMIN 500MG TABLET SANDOZ MANUFACTURER", "ATORVASTATIN HYDROCHLORIDE METFORMIN HYDROCHLORIDE", "METFORIN HYDROCHLORIDE METFORMIN HYDROCHLORIDE", "METFORMINI HCL METFORMIN HYDROCHLORIDE TA", "GLUCOPHAGE METFORMING HYDROCHLORIDE", "METFORMIN 500 MGS", "METFORMIN HCL GLIPIZIDE", "METFORMIN HYDROHCLORIDE METFORMIN HYDROHCLORIDE", "METIGUANIDE METFORMIN HYDROCHLORIDE", "METIGUANIDE METFORMIN HYDROCHLORIDE TABLET", "GLUCOPHAGE UNSPECIFIED METFORMIN HYDROCHLORIDE", "GLUCOPHAGE METFORMIN 850 MILLIGRAM", "METFORMIN ER METFORMIN HYDROCHLORIDE 500 MILLIGRAM TABLETS", "METFORMIN HYDROCHLORIDE WATSON LABORATORIES METFORMIN HDYROCHLORIDE", "DIABEX METFORMIN HYDROCHLORIDE", "GLUCPHAGE XR METFORMIN HYDROCHLORIDE", "METFORMIN HYDROCHLORDE", "METFORMIN 1000 MG BID HS502", "METFORMIN HYDROCHLORIDE TABLETS 1000 MG PUREPA", "METFORMIN HYDROCHLORIDE ROSIGLITAZONE METFORMIN HYDROCHLORIDE ROSIGL", "METFORMIN HCL METFORMIN", "METFORMIN ER METFORMIN HYDROCHLORIDE", "GLUCOPHAGE METFORMIN HDYROCHLORIDE", "METFROMIN METFORMIN", "GLUCOVANCE METFORMIN HYDROCHLORIDE GLIBENCLAMIDE", "METFORMIN TABLET", "METFORMIN HYDROCHLORIDE METFORMIN HYDROHLORIDE", "METFORMIN 500 MG ONE A", "DIABEX TABLET METFORMIN HYDROCHLORIDE", "METFORMIN HYDROCHLORIDE METFORMIN HYDROCHORIDE", "METFORMIN HCL METFORMIN HYDROCHLORIDE 500 MILLIGRAM", "METFORMIN METFORMIN UNKNOWN", "METFORMIN HYDROCHLORIDE TABLETS USP 100 MG PUREPAC", "METFORMIN HYDROCHLORIDE TABLETS USP 1000 MG PUREPAC", "METFORMINE MERCK METFORMIN HYDROCHLORIDE TABLET", "METFORMIN HCL EXTENDED RELEASE", "GLUFORMIN METFORMIN HYDROCHLORIDE", "METFORMIN XR 500", "METFORMIN METFORMIN 3 DOSE FORM", "GLJUCOPHAGE METFORMIN", "METFORMIN 850MG TABLETS", "METFORMIN METFORMIN HYDROCHLORIDE", "GLUFORMIN METFORMIN HYDROCHLORIDE", "METFORMIN METFORMIN TAF", "METFORMIN TA", "METFORMIN HYDRCOHLORIDE", "METFORMIN METFORMIN0 1000 MILLIGRAM", "GLUCOPHAGE METFORMIN HYDORCHLORIDE", "GLUCOPHAGE XR METFORMIN HYDROCHLORIDE", "METGORMIN METFORMIN 500 MILLIGRAM", "GLIBOMET METFORMIN HYDROCHLORIDE", "METFORMIN 500 MG", "METOFORMIN METFORMIN", "NMETFORMIN", "METFORMIN HYDROCHLROIDE", "GLIFAGE METFORMIN NR", "METFORMIN 800MG", "METFORMIM HYDROCHLORIDE METFORMIN HYDROCHLORIDE", "METFORMIN METHFORMIN 500 MILLIGRAM", "METFORMIM METFORMIN", "GLIPIZIDE METFORMIN HYDROCHLORIDE METFORMIN HYDROCHLORIDE GLIPIZIDE", "METFORMIN MERCK METFORMIN HYDROCHLORIDE", "METFORMIN HYDROCHLORIDE METFORMIN HYDOCHLORIDE", "METFORMINE MERCK METFORMIN HYDROCHLORIDE", "GLYBURIDE 5MG METFORMIN HCL", "GLYBURIDE 2 5MG METFORMIN HCL 500MG", "GLYBURIDE W METFORMIN HYDROCHLORIDE", "GLUCOPHAGE SCH METFORMIN HYDROCHLORIDE", "GLIBENCLAMIDE W METFORMIN HYDROCHLORIDE", "METFORMIN NGX METFORMIN UNKNOWN", "METFORMIN METFORMIN 1000 MILLIGRAM", "GLUCOPAGE METFORMIN", "METFORMIN 500MG", "METFORMIN HYDROCHLORIDE ROSIGLITAOZNE METFORMIN HYDROCHLORIDE ROSIGL", "GLUCONORMIN METFORMIN", "METFORMIN HYDROCHLORIDE ROSIGLITAZONE METFORMIN HYDROCHLORIDE ROSIG", "GLIBOMET GLIBENCLAMIDE METFORMIN HYDROCHLORIDE", "METFORMIN NGX METFORMIN UNKNOWN", "DIABEX METFORMIN HYDROCHLORIDE", "DIABOMET METFORMIN GLYCYCLAMIDE", "METFORMIN EMBONATE METFORMIN EMBONATE", "METFORMIN 2000 MG", "GLUCOPAHTE METFORMIN HYDROCHLORIDE", "METFORMIN HEUMANN METFORMIN", "METFORMIN 500 MG ONE DAILY", "METFORMIN 500MG BRISTOL MEYERS SQUIB", "METFORMINE", "METFORMIN 500 MG MG TID", "METFORMIN GENERIC", "METFORMIN 500 FOUR QD", "GLIPIZIDE METFORMIN HYDROCHLORIDE GLIPIZIDE METFORMIN HYDROCHLORIDE", "METFORMIN HYDROCHLORIDE METFORMIN H HYDROCHLORIDE", "METFORMIN 850MG", "GLUCOPAHGE METFORMIN HYDROCHLORIDE", "MELLBIN METFORMIN HYDROCHLORIDE", "GLUCOPAGE METFORMIN HYDROCHLORIDE 1000 MILLIGRAM", "AVANDAMET ROSIGLITAZONE MALEATE METFORMIN HYDROCHLORIDE", "METFORMIN NGX METFORMIN", "AVANDAMET TABLETS ROSIGLITAZONE METFORMIN", "GLYBURIDE METFORMIN 2 5 500 BMS PAR", "GLUCOHAGE METFORMIN", "METFORMIN 850 MG", "BI EUGLUCON GLIBENCLAMIDE METFORMIN", "METFORMIN 1000 MG", "GLUCOVANCE GLYBURIDE METFORMIN", "GLYBURIDE METFORMIN 5MG 500MG IVAX", "GLUCOPHAGE METFORMIN 1000 MILLIGRAM PILL", "METFORMIN NGX", "METFIN METFORMIN HYDROCHLORIDE", "METFORMIN NGX METFORMIN", "METFORMIN NGX MIETFORMIN", "METFORMIN NGX METFORMIN", "METFORMIN HCL GLUCOPHAGE", "SIL NORBORAL GLIBENCLAMIDE METFORMIN", "GLUOCPHAGE METFORMIN HYDROCHLORIDE", "METFORMIN HYDROCHLORIDE METFROMIN HYDROCHLORIDE0", "METFORMIN ER 500 MG PUREPAC", "GLIPIZIDE METFORMIN HYDROCHLORIDE", "METFORMIN XR 750MG", "METFORMIN 1000MG", "METFORMIN HYDROCHLORIDE METHFORMIN HYDROCHLORIDE", "METFORMIN 850MG", "MERFROMIN HYDROCHLORIDE METFORMIN HYDROCHLORIDE", "DIABEX METFORMIN HYDROCHLORIDE TA", "METFORMIN 500MG", "METFORMIN 1000MG WATSON", "METFORMIN 1000", "METFORMIN 850 BID", "GLUCOPAHGE METFORMIN", "METFORMIN SANDOZ NGX METFORMIN FILM COATED TABLET", "METFORMIN ALPHARMA METFORMIN HYDROCHLORIDE", "WELBIN METFORMIN HYDROCHLORIDE", "METFORMIN HYDROCHLROIDE METFORMIN HYDROCHLORIDE", "METFORM HYDROCHLORIDE METFORMIN HYDROCHLORIDE", "GLUCOPAGE ARON METFORMIN HYDROCHLORIDE", "METFORMIN 1000MG", "METFORMI METFORMIN", "GLIMEPERIDE METFORMIN", "METFORMIN HYROCHLORIDE ROSIGLITAZONE METFORMIN HYDROCHLORIDE ROSIGLI", "METFORMIN 850MG", "METFORMIN ROSIGLITAZONE", "GLUCOPHATE METFORMIN", "METFORMIN 500 MG TAB", "METFORMIN 850 MG BID", "METFORMIN METFORMIN METFORMIN", "AVANDAMET METFORMIN HYDROCHLORIDE ROSIGLITAZONE", "DIABEX METFORMIN HYDROCHLORIDE", "METFORM METFORMIN", "GLUCOVANCE GLINENCLAMIDE METFORMIN HYDROCHLORIDE", "GLUCOPHAGE METFORMIN 00082701", "GLUCOPHAGE METFORMIN 00082701", "GLUCOPHAGE METFORMIN 00082701", "METFORMIN W ROSIGLITAZONE", "METAFORMIN METFORMIN", "METFORMIN 500 MG", "METFORMIN HYDROCHLORIDE METFORMIN", "STAGID METFORMIN EMBONATE", "GLIPIZIDE METFORMIN", "METFORMIN HCL XR TABS 500 MG", "LEVOTHYROXINE GLUCOPHAGTE METFORMIN", "METFORMINE HCL", "METFORMIN HYDROCHORIDE", "METFORMIN METFORMIN HYDROCHLORIDE METFORMIN", "METFORMIN HYDROCHLORIDE METFORMIN HYDROCHLORIDE UNKNOWN", "GLUCOPAGE METFORMIN HYDROCHLORIDE", "METFORMIN 1000 MG TABLET APOTEX", "METFORMIN GLYBURIDE 500 25", "METFORMIN 500 MG BID", "METFORMIN 1000MG TABLET WATSON", "GLYBURIDE METFORMIN 25 500", "NOVO METFORMIN", "METAGLIP GLIPIZIDE METFORMIN HYDROCHLORIDE", "GLIBOMET GLIBCENCLAMIDE METFORMIN HYDROCHLORIDE", "GLUCOPHAGE METFORMIN HYDROCHOLRIDE", "METFORMIN SA", "ROSIGLITAZONE METFORMIN", "AVANDAMET METFORMIN ROSIGLITAZONE MALEATE", "GLUCOFORMIN METFORMIN HYDROCHLORIDE", "GLYBURIDE METFORMIN HCL TABS", "GLUCOPHAGE METFORMIN 00082701", "METFORMIN 500 MG", "MERCK METFORMINE", "METFORMIN 500 MG CARACO", "METFORMIN PAMOATE", "GLUCOPHAGE METFORMIN 00082701", "METFORMIN MEFORMIN", "METFORMIN 500MG UNKNOWN", "ADIMET METFORMIN HYDROCHLORIDE", "GLUCOVANCE 2 5 MG 500 MG TABLET METFORMIN HYDROCHLORIDE GLIBENCLAM", "DIABETASE NGX METFORMIN", "DIABEX METFORMIN HYDROCHLORIDE METFORMIN HYDROCHLORIDE", "METFORMIN 500 MG IVAX PHARMACEUTICALS", "ORABET METFORMIN HYDROCHLORIDE", "CELEXA CITALOPRAM HYDROBROMIDE PHAGE ABIC METFORMIN HYDROCHLORIDE", "METFORMIN HYDROCHLORIDE 500 MG", "GLUCOPHAGE ABIC METFORMIN HYDROCHLORIDE TABLET 850 MG", "METFORMINE BIOGARAN", "DIAFORMIN TABLET METFORMIN HYDROCHLORIDE", "GLIBOMET GALENIC GLIBENCLAMIDE METFORMIN", "GLIBOMET GLIBENCLAMIDE METFORMIN HYDROCHLORIDE", "AVANDAMET METFORMIN HYDROCHLORIDE ROSIGLITAZONE MALEATE", "MERCKFORMIN METFORMIN HYDROCHLORIDE", "GLIBENCLAMIDE W METFORMIN", "GLYBURIDE 5MG METFORMIN HCL 500MG", "METFORMIN METFORMIN 850 MILLIGRAM", "MERCKFORMIN METFORMIN HYDROCHLORIDE", "GLUCOPHAGE METFORMIN HYDROCHLORIDE METFORMIN", "GLIBOMET GALENIC GLIBENCLAMIDE METFORMIN", "ROSIGLITAZONE METFORMIN", "METFORMIN METFORMIN", "METPHORMAX METFORMIN HYDROCHLORIDE", "METFORMIN HCL XR TABS", "METFORMIN 850 MG CARACO", "METFORMIN 500MG TABLETS", "GLUCOVANCE METFORMIN GLYBURDIE", "CLUCOPHAGE METFORMIN HYDROCHLORIDE", "METFORMIN HYDROCHLORIDE METFORMIN HYDROCHLORIDE S", "METFIN METFORMIN HYDROCHLORID", "METFORMIN HYDROCHLORIDE TABLETS 500MG 625MG 750MG 850MG 1000MG", "METFORMIN HYDROCHLORIDE 500MG METFORMIN HYDROCHLORIDE TABLET 500MG", "DIABEX METFORMIN HYDROCHLORIDE0", "METFORMIN 500MG", "MERCKFORMIN METFORMIN HYDROCHLORIDE", "RISIDON METFORMIN HYDROCHLORIDE", "METFORMIN 850 MG BID", "GLYBURIDE 5 MG METFORMIN", "METFORMIN 1000 MG", "ORABET METFORMIN", "METFORMIN ER TABS 6065 500MG", "METFORMIN 1000 MG EON LABS", "METFORMIN HYDROCHLORIDE TABLETS 1000 MG PUREPAC", "GLUCOPHAGE METFORMIN FILM TABLET", "FORTEMET METFORMIN HYDROCHLORIDE", "GLYCORIN METFORMIN HYDROCHLORIDE", "MEGLUCON METFORMIN HYDROCHLORIDE", "METFORMIN ER 500 MG PAR", "GLYBURIDE W METFORMIN GLYBURIDE W METFORMIN", "METFIN METFORMIN", "GLUCOPHAGE METFORMIN HYDROCHLORIDE METFORMIN", "GLUCOPHAGE SLOW RELEASE METFORMIN HYDROCHLORIDE TABLET", "GLUCOPHAGE SLOW RELEASE METFORMIN HYDROCHLORIDE TABLET", "METFORMIN MFETFORMIN", "GLUCINAN METFORMIN CHLOROPHENOXYACETATE TABLETS", "METFORMIN ER 500 MG 2 BID", "METFORMIN BIPHASIC TABS 500 MG", "METFORMIN GLIPIZIDE", "GLUCINAN METFORMIN CHLOROPHENOXYACETATE TABLETS", "METFORMAIN METFORMIN", "METFORMN METFORMIN", "NOVO METFORMIN METFORMIN", "METFORMIN TABLETS BP 500 MG METFORMIN", "NELBIS METFORMIN HYDROCHLORIDE", "GLYBURIDE METFORMIN 5 500", "GLIBENCLAMIDE METFORMIN", "METFORMIN APOTEX", "METFORMIN HYDROCHLORIDE AND ROSIGLITAZONE MALEATE", "METFORMIN XL", "GLYBURIDE W METFORMIN GLIBENCLAMIDE METFORMIN", "METFORMIN ER", "METFORMINE FORTE", "METFORMIN HYDROCHLORIDE EXTENDED RELEASE TABLETS 750 MG PUREPAC", "METAGLIP GLIPIZIDE METFORMIN", "ACTOPLUS MET PIOGLITAZONE HYDROCHLORIDE METFORMIN HYDROCHLORIDE TA", "METFORMINA", "NOVALGIN SCH METFORMIN SODIUM MONOHYDRATE", "GLYBURID METFORMIN 5 500 TABS", "METFORMIN METFORMIN 1000 MILLIGRAM TABLETS", "GLUCOPHAGR METFORMIN HYDROCHLORIDE", "METFORMIN TABLETS 500MG METFORMIN HYDROCHLORIDE TABLET", "GLYURIDE METFORMIN", "METFORMIN NGX METFORMIN TABLET", "METFORMIN XR METFORMIN", "METFORMIN METFORMIN 00082701", "GLYBURIDE 1 25MG METFORMIN HCL 250MG", "GLIBENCLAMIDE PLUS METFORMIN", "APO METFORMIN", "METFORMIN IMETFORMIN", "ACTO METFORMIN", "METFORMIN CHLORHYDRATE", "DIAFORMIN METFORMIN", "GLYBURIDE 2 5 MG METFORMIN HCL", "AVANDAMET ROSGLITAZONE METFORMIN", "METAGLIP GLIPIZIDE METFORMIN", "MELLIMET NGX METFORMIN TABLET", "AVANDAMET METFORMIN HYDROCHLORIDE ROSIGILITAZONE", "GLYBURIDE 5MG METFORMIN", "DIABEX METFORMIN", "METFORMIN METFORMIN SOLUTION", "METFORMIN ER METFORMIN", "GLUCOPHAGE 00032601 METFORMIN", "AVANDAMET ROSIGLITAZONE MELEATE AND METFORMIN HYDROCHLORIDE", "GLUCOPHAGE BOEHRINGER MANHEIM METFORMIN HYDROCHLORIDE", "METFORMIN GLIBENCLAMIDE", "GLYBURIDE 5 MG METFORMIN HCL", "DIFORMIN METFORMIN HYDROCHLORIDE", "METFORMIN GLIPIZIDE", "GLYBURIDE METFORMIN MIX", "GLIPIZIDE METFORMIN GLIPIZIDE METFORMIN", "GLYBURIDE METFORMIN 5 500", "ACTOS METFORMIN", "GLUCOPHAGE METFORMIN 500 MILLIGRAM", "METFORMIN METFORMIN TABLET 850 MG", "FORTAMET METFORMIN", "METFORMIN BIOCHEMIE NGX METFORMIN UNKNOWN", "METFORMIN 1000 MG PO BID", "ACTOS METFORMIN", "METFORMIN ACTOS COMBINATION", "AVANDAMET ROSIGLITAZONE MALEATE AND METFORMIN HYDROCHLORIDE TABLETS", "METFORMINE HYDROCHLORIDE METFORMIN HYDROCHLORIDE", "AVANDAMET METFORMIN HYDROCHLORIDE ROSGLITAZONE", "METFORMIN HYDROCHLORIDE METFORMIN HYDROCHLORIDE TABLET 1000MG", "GLYB METFORMIN", "GLIPIZIDE METFORMIN", "GLUCOPHAGE METFORMIN HYDROCHLORIDE TABLET", "DIABETES MEDICATION METFORMIN HYDROCHLORIDE", "METFORMIN METFORMIN", "METFORMIN 500MG BID 2 6 03", "PIOGLITAZONE METFORMIN", "DIABEN METFORMIN HYDROCHLORIDE", "METFORMIN TABLETS 500MG METFORMIN TABLETS", "MEGLUCON METFORMIN HYDROCHLORIDE", "METFORMIN METFORMIN", "GLYBURIDE METFORMIN METFORMIN GLIBENCLAMIDE TABLETS", "METFORMIN METFORMIN", "METFORMIN 500MG TWO PO QHS", "GLYBURIDE METFORMIN METFORMIN GLIBENCLAMIDE", "DIABEX METFORMIN HYDROCHLORIDE", "METFORMIN METFORMIN 500 MILLIGRAM TABLETS", "GLUCOPHAGE 00082701 METFORMIN", "METFORMIN HYDROCHLORIDE TABLETS 1000 MG PUREPAC METFORMIN HYDROCHLOR", "METFORMIN HYDROCHLORIDE TABLETS 1000 MG PUREPAC METFORMIN HYDROCH", "METFORMIN HYDROCHLORIDE", "METFORMIIN METFORMIN", "GLYBURIDE METFORMIN SIL NORBORAL", "METFORMIN METFO0RMIN", "METFORMIN GLYBURIDE", "GLUCOPHAGE METFORMIN TABLET", "GLYBURIDE W METFORMIN METFORMIN GLIBENCLAMIDE", "ACTOS PLUS METFORMIN", "FORTAMET METFORMIN HYDROCHLORIDE", "METFORMIN TIME RELEASED", "METFORMIN METFORMIN HYDROCHLORIDE TABLET 500MG", "DIFORMIN RETARD METFORMIN HYDROCHLORIDE", "METFORMIN HEXAL NGX METFORMIN FILM COATED TABLET 1000MG", "METFORMIN GLIBENCLAMIDE", "YMETFORMIN", "ACTOS PLUS WITH METFORMIN", "METFORMIN TABLET METFORMIN", "METFORMIN EXTENDED RELEASE", "METFORMIN HYDROCHLORIDE TABLETS 500 MG PUREPAC", "SIL NORBORAL GLIBENCIAMIDE METFORMIN", "STAGID METFORMIN EMBONATE", "GLCOPHAGE METFORMIN", "METFORMIN METFORMIN ER", "METFORMIN MERCK 850 MG 850 MG FILM COATED TABLET METFORMIN", "DIABETEX METFORMIN HYDROCHLORIDE", "METFORMIN FAMILY METFORMIN HYDROCHLORIDE TABLET", "METFORMIN METFORMIN HYDROCHLORIDE UNKNOWN", "GLUCOPHAGE METFORMIN METFORMIN", "METAGLIP METFORMIN HYDROCHLORIDE GLIPIZIDE", "METFORMIN HCL ER", "MEDET METFORMIN HYDROCHLORIDE TABLETS", "METFORMIN QUALIMED 850 MG 850 MG FILM COATED TABLET METFORMIN", "GLUCOVANCE GLIBENCLAMIDE METFORMIN HYDRCHLORIDE", "METFORMIN STADA", "DIABET METFORMIN HYDROCHLORIDE", "METFORMIN HYDROCHLORIDE NGX METFORMIN UNKNOWN", "METAGLIP GLIPIZIDE METFORMIN HYDROCHLORIDE", "METFORMIN XR METFORMIN", "METFORMIN HCL APO", "METFORMIN ER 750MG", "METFIN NGX METFORMIN FILM COATED TABLET 850MG", "TORASEMIDE NGX METFORMIN FILM COATED TABLET 850MG", "METAGLIP GLIPIZIDE METFORMIN HYDROCHLORIDE", "METFORMIN UNK UNKNOWN", "METFORMIN HYDROCHLORIDE CON", "METFORMIN NGX METFORMIN TABLET", "METFORMIN HYDROCHLORIDE ROSIGLITAZONE METFORMIN HYDROCHLORIDE ROSIGLI", "METFORMIN PIOGLITAZONE METFORMIN PIOGLITAZONE", "METFORMIN PIOGLITAZONE GLIMEPIRIDE GLIMEPIRIDE PIOGLITAZONE HYDROCHL", "GLUCOPHAGE METFORMIN HYDROCHLORIDE METFORMIN HYDROCHLORIDE", "METFORMIN METFORMIN HCL", "METFORMIN HCL TABLETS NGX METFORMIN TABLET", "METFOREM METFORMIN HYDROCHLORIDE", "METFORMIN HCI", "METORMIN METFORMIN", "METOPROLOL METFORMIN TARTRATE", "GLUCONORM METFORMIN", "METFORMIN HYDROCHLORIDE TABLETS 1000 MG PUREPAC METFORMIN HYDROCHL", "METFORMIN METFORMIN 1000 MILLIGRAM", "METFORMIN 500MG", "GLUCOFORMIN METFORMIN HYDROCHLORIDE TABLET", "GLUCOPHAGE 00082701 METFORMIN", "GLUCOPHAGE 00082701 METFORMIN", "GLUCOPHAGE 00082701 METFORMIN", "GLUCOPHAGE 00082701 METFORMIN", "METFORMIN HYDROCHLORIDE GLIBENCLAMIDE GLYBURIDE METFORMIN", "METFORMIN PIOGLITAZONE", "METFORMIN WITH GLYBURIDE", "ACTOS AND METFORMIN", "METFORMIN SR", "METFORMINE RPG 850 MG COMPRIME PELLICULE METFORMIN HYDROCHLORIDE UNK", "GLUCOHAGE METFORMIN HYDROCHLORIDE", "MEFORMIN METFORMIN", "ACTOPLUS METFORMIN", "GLYBURIDE METFORMIN 5 500MG", "GLUBURIDE METFORMIN", "METFORMIN UNKNOWN UNKNOWN", "GLUCOPHAGE 01224501 METFORMIN", "METFORMIN 1000 MG BID", "METFORMIN 850 MG TID", "GLYCORAN METFORMIN HYDROCHLORIDE", "GLYBMETFORMIN", "COMBINATION METFORMIN", "METFORMIN HYDROCHLORID UNKNOWN", "METFORMIN BASICS 500MG METFORMIN HYDROCHLORIDE UNKNOWN", "GLUBURIDE METFORMIN", "METFORMIN NGX METFORMIN TABLET 500MG", "MEDET METFORMIN HYDROCHLORIDE", "METFORMIN HYDROCHLORIDE METFORMIN", "GLIBOMET GLYBURIDE METFORMIN", "METFORMIN NGX METFORMIN TABLET 850MG", "RISIDON TABLET METFORMIN", "GLICONORM COATED TABLET METFORMIN GLIBENCLAMIDE", "OXYBUTYNIN METFORMIN 00082701", "MEDET METFORMIN HYDROCHLORIDE", "METFORMIN UNK", "DIAMBEN METFORMIN", "GLUCOPHAGE METFORMINE", "METFORMIN HYDROCHLORIDE METFORMIN HYROCHLORIDE", "METFORMIN SANDOZ NGX METFORMIN FILM COATED TABLET 500MG", "GLYBURIDE WITH METFORMIN", "METFORMIN ACTOS", "MEFTFORMIN METFORMIN METFORMIN", "DIANBEN METFORMIN", "METFORMIN 00082702 CON", "GLYBURIDE PLUS METFORMIN", "DI AMBEN METFORMIN 850 DOSAGE FORMS", "ACTOPLUS PIOGLITAZONE METFORMIN", "ROSIGLITAZONE MALEATE AND METFORMIN HCL", "GLYBURIDE METFORMIN 5 500 MG", "METFORMINI HYDROCHLORIDUM", "METFORMIN 00082701 METFORMIN", "METFORMIN GLUCOVANCE", "METFORMIN 2500 MG", "METFORMIN CON", "GLYBURIDE METFORMIN CON", "METFORMIN TABLET METFORMIN", "GLUCOPHAGE METFORMIN 00082701", "METFORMIN 00082702 METFORMIN HYDROCHLORIDE", "AVANDAMET METFORMIN HYDROCHLORIDE ROSIGLITAZONE MALEATE CAPSULE", "GLUCOPHAGE 00082701 METFORMIN TABLET", "COMPETACT PIOGLITAZONE HYDROCHLORIDE METFORMIN HYDROCHLORIDE", "GLUCOPHAGE S METFORMIN HYDROCHLORIDE METFORMIN HYDROCHLORIDE", "METFORMIN HYDROCHLOIRDE METFORMIN", "METFROMIN METFORMIN 500 MILLIGRAM", "DIABETEX METFORMIN HYDROCHLORIDE", "METFORMIN HYDROCHLORIDE AND PIOGLITAZONE HYDROCHLORIDE", "DIABEX 1 GRAM TABLET METFORMIN", "DIFORMIN METFORMIN HYDROCHLORIDE TABLET", "METFORMINI", "FORMIN METFORMIN HYDROCHLORIDE", "ORABET METFORMIN", "METFORMIN METFORMIN HYDROCHLORIDE", "GLUCOPHAGE 850 MG METFORMIN", "INSULIN METFORMIN", "GLUCOPHAGE USA METFORMIN HYDROCHLORIDE", "DIANBEN 850 MG TABLET METFORMIN", "METFORMIN METFORMIN UNKNOWN", "GLUCOPHAGE 00082701 METFORMIN TABLET", "ORABET METFORMIN HYDROCHLORIDE0", "INSULIN METFORMIN", "GLYBURID METFORMIN", "GLUCOPHAG METFORMIN HYDROCHLORIDE", "METFORMIN AND GLYBURIDE", "METFORMIN SANDOZ NGX METFORMIN UNKNOWN 850MG", "METFORMIN ER", "METFORMIN GLYBURIDE COMBINATION", "GLUMETZA METFORMIN XL", "METFORMIN METFORMIN UNKNOWN", "GLUCOPAHGE METFORMIN", "METFORMIN GLIBENCLAMIDE GLUCOVANCE", "AVANDAMET METFORMIN HYDROCHLORIDE ROSIGLITAZONE MALEATE", "METFORMIN GLIBENCLAMIDE GLUCOVANCE 01503701", "METFORMIN HYDROCHLORIDE GLUCOPHAGE XR", "METFORMIN METFORMIN TABLETS", "METFORMIN HYDROCHLORIDE ROSIGLITAZONE MALEATE AVANDAMET", "ROSIGLITAZONE MALEATE METFORMIN HYDROCHLORIDE FORMULATION ROSIGLIT", "METFORMIN METFORMIN", "METFORMIN HYDROCHLORIDE ROSIGLITAZONE MALEATE AVANDAMET", "METRION METFORMIN HYDROCHLORIDE", "RATIO METFORMIN", "METFORMINE METFORMIN HYDROCHLORIDE", "METFORMIN NGX METFORMIN UNKNOWN", "METFORMIN 500MG TABLETS METFORMIN", "METRION METFORMIN HYDROCHLORIDE", "METFORMIN TABLETS", "METFORMIN NGX METFORMIN UNKNOWN 500MG", "METFORMIN NGX METFORMIN TABLET 500MG", "METFORMIN 500MG TABLETS METFORMIN UNKNOWN", "GLYBURIDE METFORMIN METFORMIN GLIBENCLAMIDE", "MESCORIT METFORMIN HYDROCHLORIDE", "GLUCOVANCE TABLET METFORMIN GLIBENCLAMIDE", "SITAGLIPTIN METFORMIN", "PIOGLITAZONE HYDROCHLORIDE METFORMIN HYDROCHLORIDE CODE NOT BROKEN", "METFIN NGX METFORMIN UNKNOWN", "MELBIN METFORMIN HYDROCHLORIDE TABLET", "METFORMIN 1A PHARMA NGX METFORMIN UNKNOWN 850", "METFORMIN HYDROCHLORIDE METFORMIN HYDROCHLORIDE UNKNOWN", "METFORMIN HCL METFORMIN HYDROCHLORIDE TABLETS", "LMF237 VS VILDAGLIPTIN VS METFORMIN", "COMPETACT PIOGLITAZONE HYDROCHLORIDE METFORMIN HYDROCHLORIDE PRIOR", "GLUCOVANCE GLIBENCLAMIDE METFORMIN HYDROCHLORIDE TABLET", "METFORMIN METFORMIN TABLET 500 MG", "GLYBURIDE W METFORMIN HYDROCHLORIDE GLIBENCLAMIDE METFORMIN HYDROCHL", "DIABEX TABLET METFORMIN", "GLUCOPHAE METFORMIN", "GLIPIZIDE W METFORMIN GLIPIZIDE METFORMIN", "GLYBURIDE METFORMIN 5 500MG TABLET", "AVANDAMET METFORMIN HYDROCHLORIDE ROSIGILATZONE MALEATE", "AVANDAMMET METFORMIN ROSIGILITAONE MALEATE", "METFORMIN METFROMIN", "GLUCOPHGE METFORMIN", "GLUCOPHAGE XR METFORMIN HYDROCHLORIDE", "BLYBURIDE W METFORMIN HYDROCHLORIDE GLIBENCLAMIDE METFORMIN HYDROCHL", "GLUCOVANCE GLIBENCLAMIDE METFORMING HYDROCHLORIDE", "METFORMIN HEXAL NGX METFORMIN FILM COATED TABLET 850MG", "GLUCOPHAGE XR METFORMIN HYDROCHLORIDE", "METFORMIN HYDROCHLORIDE METFORMIN HYDROCHLORIDE", "GLYBURIDE WMETFORMIN HYDROCHLORIDE GLIBENCLAMIDE METFORMIN HYDROCHLO", "METFORMIN MEPHA METFORMIN HYDROCHLORIDE", "METFOMIN METFORMIN", "GLUCOVANCE GLIBENCALMIDE METFORMIN HYDROCHLORIDE", "METAGLIP GLIPIZIDE METFORMIN HYDROCHLORIDE", "GLUCOPHAGE USA METFORMIN HYDROCHLORIDE", "GLUCOVANCE GLIBENCAMIDE METFORMIN HYDROCHLORIDE", "FORMIDIAB METFORMIN", "GLUCOPHAGE METFORMIN HYDROCHLORIDE TABLETS", "COMPETACT PIOGLITAZONE HYDROCHLORIDE METFORMIN HYDROCHLORIDE", "GLIFAGE 850 MG 850 MG TABLET METFORMIN", "GLIFAGE 500 MG 500 MG TABLET METFORMIN", "CO METFORMIN TABLETS LOT NO EXPIRY DATE UNKNOWN", "FLIFAGE 500 MG 500 MG TABLET METFORMIN", "METFORMIN GLYBURIDE METFORMIN HYDROCHLORIDE GLIBENCLAMIDE", "METFORMIN HYDROCHLORIDE ER TABLETS 500MG", "METFORMIN HCL ER TABS 571 500MG", "METFORMIN HCL ER TABS 571 500MG ANDRX", "METFORMIN HCL ER TABS 571 500MG", "METFORMIN HCL ER TABS 500MG", "METFORMIN TABLETS METFORMIN HYDROCHLORIDE", "GLUCOVANCE METFORMIN HYDROCHLORIDE GLIBENCLAMIDE METFORMIN HYDROCH", "DIANBEN METFORMIN HYDROCHLORIDE", "METFORMIN HYDROCHLORIDE PIOG", "MEDET METFORMIN HYDROCHLORIDE", "METFORMIN HCL ER 500MG SUN PHARMACEUTICALS", "METFORMIN 500MG TABLETS METFORMIN HYDROCHLORIDE", "MESCORIT METFORMIN HYDROCHLORIDE", "METFORMIN METFORMIN 500 MG", "METFORMIN METFORMIN TABLET 850 MG", "GLIBURIDE METFORMIN HYDROCHLORIDE", "COMPETACT PIOGLITAZONE HYDROCHLORIDE METFORMIN HYDROCHLORIDE", "METFORMIN 500MG TABLETS", "METFORMINA METFORMIN METFORMIN", "METFORMIN METFORMIN HYDROCHLORIDE", "METFORMIN 00082701 METFORMIN", "COMPETACT PIOGLITAZONE HYDROCHLORIDE METFORMIN HYDROCHLORIDE", "METFORMIN HYDROCHLORIDE SITAGLIPTIN", "METOFORMIN METFORMIN METFORMIN", "GLUCOVANCE GLIBENCLAMIDE METFORMIN", "METFORMIN 00082702", "GLYBURIDE METFORMIN GLIBENCLAMIDE METFORMIN HYDROCHLORIDE", "GLUMET 00082701 METFORMIN", "GLCOVANCE 01182201 GLIBENCLAMIDE METFORMIN HYDROCHLORIDE", "METFORMIN UNKNOWN UNTIL UNKNOWN", "GLUCOPHAGE ER METFORMIN", "METFORMIN UNKNOWN", "METFORMIN NGX METFORMIN TABLET", "METFORMIN HCL TABLET", "METFORMIN METFORMIN HYDROCHLORI", "ROSIGLITAZONE MALEATE AND METFORMIN HYDROCHLORIDE METFORMIN W ROSIGLIT", "METFORMIN METFORMIN 00082701", "METFORMIN 850", "METFORMIN METFORMIN 00082701", "MESCORIT METFORMIN HYDROCHLORIDE", "GLIBENCLAMIDE METFORMIN GLIBENCLAMIDE METFORMIN", "GLUCOVANCE GLIBENCLAMIDE METFORMIN HYDROCLORIDE", "METFORMIN NGX METFORMIN UNKNOWN 850MG", "METFIN NGX METFORMIN UNKNOWN 850MG", "METFORMIN BASICS 1000MG", "GLYBURIDE GLIBENCLAMIDE METFORMIN HYDROCHLORIDE", "METFORMIN METFORMIN UNSPECIFIED", "GLUCOVANCE GLIBENCLAMIDE METFORMIN HYDROCHLORIDE TAB", "METFOMRIN METFORMIN", "GLUCOPHAGE METFORMIN TABLET", "GLUCOVANCE BLIGENCLAMIDE METFORMIN HYDROCHLORIDE", "METFORMIN GLIPIZIDE TABLET", "METAGLIP GLIPIZIDE METFORMIN HYDROCHLORIDE TABLET", "METFORMIN METFORMIN 00082701", "GLUCOVANCE 01182201 GLIBENCLAMIDE METFORMIN HYDROCHLORIDE", "GLUCOPAHGE USA METFORMIN HYDROCHLORIDE", "GLUCOPHAGE USA METFORMIN HYDROCHLORIDE", "GLIPERIDE METFORMIN", "GLICONORM GLIBENCLAMIDE 5 MG METFORMIN 500 MG", "METFORMIN METFORMIN", "METFORMIN 00082701 METFORMIN", "METFORMIN METFORMIN", "METFORMIN METFORMIN", "METFORMIN 500MG TABLETS BP", "METFORMIN HYDROCHLORIDE ER", "GLUCOPHAG XR METFORMIN HYDROCHLORIDE", "AVANDAMET METFORMIN HYDROCHLORIDE ROGISLITAZONE MALEATE", "SIOFOR METFORMIN HYDROCHLORIDE METFORMIN HYDROCHLORIDE", "FORTAMET METFORMIN HYDROCHLORIDE", "METFORMIN 2550MG", "METAGLIP GLIPIZIDE METFORMIN HYDROCHLORIDE", "METFORMIN HYDROCHLORIDE ROSIGLITAZONE", "METFORMIN METFORMIN UNK TO UNK", "GLUCOPHAGE USA METFORMIN HYDROCHLORIDE UNK TO UNK", "GLUCOPHAGE XR METFORMIN HYDROCHLORIDE", "AVANDAMENT METFORMIN HYDROCHLORIDE ROSIGLITAZONE MALEATE", "METFORMIN METFOMIN", "FORTAMET METFORMIN HYDROCHLORIDE", "METFORMIN HCL METFORMIN HYDROCHLORIDE", "GLYCORAN METFORMIN HYDROCHLORIDE", "COMSETACT PIOGLITAZONE HYDROCHLORIDE METFORMIN HYDROCHLORIDE", "METFORMIN 00082701", "METFORMIN HYDROCHLORIDE TABLETS 500MG 625MG 750MG 850MG 1000MG G", "METFORMIN AND RELATED", "METOFORMIN METFORMIN 00082701", "METFORMIN METFORMIN 00082701", "GLUCOPHAGE 00082702 METFORMIN HYDROCHLORIDE TABLET", "METFORMIN 000082701 METFORMIN", "GLIMEPIRIDE METFORMIN AMARYL M", "METFORMIN METFORMIN 0082701", "METFORMIN 850 METFORMIN", "COMPETACT PIOGLITAZONE HYDROCHLORIDE METFORMIN HYDROCHLORIDE 15 MG", "METFORMINE HCL PCH TABLET 1000 MG METFORMIN", "METFORMIN HYDROCHLORIDE EXTENDED RELEASE TABLETS 750MG", "DIAFORMIN METFORMIN HYDROCHLORIDE 500 MG", "MELLITRON CHLORPROPAMIDE METFORMIN", "RIOMET METFORMIN", "GLUCOPHAGE METFORMIN HYDROCHLORIDE", "JANUMET METFORMIN HYDROCHLORIDE SITAGLIPTIN PHOSPHATE", "METFORMIN ER 500 MG TEVA", "METFORMIN HYDROCHLORIDE W ROSIGLITAZONE METFORMIN HYDROCHLORIDE ROSI", "METFORMIN METFORMINI", "GLUCIOPHAGE USA METFORMIN HYDROCHLORIDE", "GLUCOPHAE METFORMIN HYDROCHLORIDE", "GLIPIZDE W METFORMIN GLIPIZIDE METFORMIN", "AVANDAMET METFORMIN HYDROCHLORIDE ROSIGLITAZONE MAELATE", "GLUCOMET 00082702 METFORMIN HYDROCHLORIDE", "GLUCOPHAGE XR METFORMIN HYDROCHLORIDE", "METFORMIN METFORM", "GLUCOPHAGE USA METFORMIN HYDROCHLORIDE", "METFORMIN 9METFORMIN", "GLUCOPHAGE USA METFORMIN HYDROCHLORIDE", "GLYBURIDE W METFORMIN GLIBENCLAMDIE METFORMIN", "GLUCOPHAGE USA METFORMIN HYDROCHLORIDE TABLET", "GLUCOPHAEG USA METFORMIN HYDROCHLORIDE", "AVANDAMET METFORMIN HYDROCHLORIDE ROSIGLTIAZONE MALEATE", "GLUCOPHAGE METFORMIN0", "ORABET THE GENERIC IS METFORMIN HYDROCHLORIDE", "AVANDAMET METFORMIN HYDROCHLORDE ROSIGLITAZONE MALEATE", "GLUCOPHAGE USA METFORMIN HYDROCHLORIDE PEN DISPOSABLE", "AVANDAMENT METFORMIN HYDROCHLORIDE ROSIGLITAZONE MALEATE", "GLUCOPHAE USA METFORMIN HYDROCHLORIDE", "GLUCOVANCE 01182201 GLIBECLAMIDE METFORMIN HYDROCHLORIDE", "COMPETACT FIOGLITAZONE HYDROCHLORIDE METFORMIN HYDROCHLORIDE", "GLYBURIDE METFORMIN METFORMIN GLIBENCLAMIDE", "METFORMIN METFORMIN TABLET", "GLYB METFORMIN 5 500", "MEDET METFORMIN HYDROCHLORIDE", "GLIPIZIDE AND METFORMIN HYDROCHLORIDE", "GLUMETZA METFORMIN HYDROCHLORIDE", "GLUCOPHAGE METFORMIN 0008271", "GLYCORAN METFORMIN HYDROCHLORIDE TABLET", "METFORMIN 1000 MG TABLETS NO PREF NAME 1000 MG", "DIABEX XR 500 MG TABLET METFORMIN", "METFORMIN TABLET MEMTFORMIN", "METFORMIN GLYBURIDE METFORMIN HYDROCHLORIDE GLIBENCLAMIDE", "GLYCORAN METFORMIN HYDROCHLORIDE", "METFORMIN TABLETS 500MG", "METFORMIN XR ANTI DIABETICS", "METFORMINA NOVARTIS", "GLUCOPHAGE XR METFORMIN HYDROCHLORIDE", "STAGID METFORMIN EMBONATE TABLET", "METFORMINA METFORMIN", "GLUCOPHAGE ER METFORMIN 00082701", "METFORMIN 850 METFORMIN", "METFORMIN HYDROCHLORIDE ATORVASTATIN CALCIUM TABLET", "DIANBEN METFORMINE", "METFORMIN HYDROCHLORIDE W ROSIGLITAZONE", "METFORMIN METFORMIN 500 MG", "METFORMIN XR ANTI DIABETICS", "COMPETACT PIOGLITAZONE HYDROCHLORIDE METFORMIN HYDROCHLORIDE", "ACTOPLUS MET PIOGLITAZONE HYDRCOCHLORIDE METFORMIN HYDROCHLORIDE T", "COMPETACT PIOGLITAZONE METFORMIN", "METFORMIN ER CON", "BLINDED METFORMIN COMP MTF", "METFORMIN MEPHA METFORMIN HYDROCHLORIDE", "METFORMIN HC", "GLUCOPHAGE USA METFORMIN HYDRCHLORIDE", "GLUCOPHAGE USA METFORMIN HYDROCHLORIDE UNK", "FORTAMET METFORMIN HYDROCHLORIDE TABLET", "DIANBEN 850 MG METFORMIN HYDROCHLORIDE", "GLUCOPHAGE USA METFORMIN HYDROCHLORIDE CAPSULE", "METFORMIN 1000 MG", "GLUCOPHAGE 00082702 METFORMIN HYDROCHLORIDE", "METFORMIN 000082702 METFORMIN HYDROCHLORIDE", "GLUCOVANCE GLIBENCLAMDIE METFORMIN HYDROCHLORIDE", "GLYBURIDE AND METFORMIN SIL NORBORAL", "METFORMIN HYDROCHLORIDE TABLETS 500 MG PUREPAC METFORMIN HYDROCHLO", "MELBIN METFORMIN HYDROCHLORIDE TABLET METFORMIN HYDROCHLORIDE", "METFORMIN GLIBENCLAMIDE GLUCOVANCE 01503701", "METFORMIN METFORMIN METFORMIN", "METAGLIP GLIPIZIDE METFORMIN HYDROCHLORIDE", "GLUCOPHAGE USA METFORMIN HYDROCHLORIDE", "METFORMIN METFORMIN0", "INSULIN HUMALOG METFORMIN", "ACTOS USA METFORMIN HYDROCHLORIDE", "GLIPIZIDE W METFORMIN GLIPZIDE METFORMIN", "METFORMIN HEXAL NGX METFORMIN FILM COATED TABLET", "GLYBURIDE AND METFORMIN GLIBOMET", "METFORMINE RANBAXY 850MG COMPRIME PELLICULE", "GLIPIZIDE AND METFORMIN HCL", "COMPETACT PIOGLITAZONE HYDROCHLORIDE METFORMIN PIOGLITAZONE 15 MG AN", "COMPETACT PIOGLITAZONE HYDROCHLORIDE METFORMIN PIOGLITAZONE", "METFIN NGX METFORMIN FILM COATED TABLET 500MG", "METFORMIN 500 MILLIGRAM", "METFORMIN COMP MTF", "METFORMIN 00082701", "METFORMIN METFORMIN 0082701", "GUCOPHAGE METFORMIN", "METFORMIN 1A FARMA", "METFORMIN 850MG TEVA", "METFORMING", "GLUCOVANCE 9GLIBENCLAMIDE METFORMIN HYDROCHLORIDE", "GLYBURIDE W METFORMIN HYDROCHLORIE GLIBENCLAMIDE METFORMIN HYDROCHLO", "METFORMIN 00082702 METFORMIN HYDROCHLORIDE TABLET", "GLUCOPHAGE USA METFORMIN HYDROCHLORIDE0", "METFORMIN METFORMIN CAPSULE", "GLYCORAN METFORMIN HYDROCHLORIDE METFORMIN HYDROCHLORIDE", "METFORMINE HCL METFORMIN HYDROCHLORIDE", "METFORMIN METFPRMIN", "PMS METFORMIN", "GLIPZIDE W METFORMIN GLIPIZIDE METFORMIN", "GLUCOPHAGEUSA METFORMIN HYDROCHLORIDE", "GLUCOPHAGE USA METFORMIN HYDROCHLORIDE", "COMPETACT PIOGLITAZONE HYDROCHLORIDE METFORMIN HYDROCHLORIDE PIOGLIT", "METFORMIN ACL", "METFORMIN HYDROCHLORIDE EXTENDED RELEASE TABLETS 500 MG PUREPAC MET", "METFORMIN WITH ACTOS", "GLYPERIDE METFORMIN", "METFORMIN METFDRMIN", "GLIPMET METFORMIN HYDROCHLORIDE GLIPIZIDE TABLET METFORMIN HYDRO", "JANUMET METFORMIN HYDROCHLORIDE", "TAB GLYCORAN METFORMIN HCL", "METFORMIN H", "METFORMIN INSULIN", "METFORMIN GLYBURIDE METFORMIN GLIBENCLAMIDE", "METFORMIN METFORMIN TABLET METFORMIN", "METFORMINE MERCK", "METFORMIN 00082702", "METFORMINE MERFORMIN HYDROCHLORIDE", "AVANDAMET TABLET METFORMIN HYDROCHLORIDE ROSIGLITAZONE MALEATE", "GLUMETZA METFORMIN HYDROCHLORIDE 500 MG", "METFORMIN METFORMIN 1000 MILLIGRAM TABLET METFORMIN", "ORABET METFORMIN HYDROCHLORIDE FILM COATED TABLET", "ORABET UNK METFORMIN HYDROCHLORIDE FILM COATED TABLET 500 MG", "ACTOPLUS MET METFORMIN HYDROCHLORIDE PIOGLITAZONE HYDROCHLORIDE", "GLICONORM METFORMIN GLIBENCLAMIDE", "SIOFOR METFORMIN HYDROCHLORIDE 850 MILLIGRAM TABLET METFORMIN HY", "METFORMIN HYDROCHLORIDE EXTENDED RELEASE TABLETS 750 MG PUREPAC ME", "ACTOSMET PIOGLITAZONE HYDROCHLORIDE METFORMIN HYDROCHLORIDE", "MEFORAL METFORMIN", "SIOFOR METFORMIN HYDROCHLORIDE 850 MILLIGRAM TABLET METFORMIN HYD", "ALTOSE METFORMIN", "METFORMIN HYDROCHLORIDE AND SITAGLIPTIN PHOSPHATE", "METFORMIN SANDOZ NGX METFORMIN FILM COATED TABLET 850MG", "METFORMIN ALIUD PHARMA", "METFORMIN HCL ER 500MG SUN PHARMACUTICAL", "METFORMIN SANDOZ", "METFORMIN MR", "METFORMIN 500 MG UNK", "DIABEX METFORMIN HYDROCHLORIDE TABLET METFORMIN HYDROCHLORIDE", "METFORMIN MERFORMIN", "METFORMIN METFORMIN HYDROCHLORID", "METFORMINE METFORMIN HYDROCHLORODE", "METFORMINE TABLETS", "METFORMIN DURA METFORMIN HYDROCHLORIDE 1000 MG", "METFORMIN HCL TABLET 500 MG", "METFORMIN HCL TABLET 1000 MG", "METFORMIN METFORMIN FORMULATION UKNOWN", "METFORMIN METBAY 500", "METFORMIN MIXTARD 00646001 INSULIN HUMAN", "GLUOPHAGE METFORMIN HYDROCHLORIDE", "METFORMIN ER 500MG SUN PHARMACEUTICAL", "METFORMIN METFORMIN 1000 MILLIGRAM TABLET METFORMIN", "METFORIN METFORMIN", "METFORMIN FORMULATION UNKNOWN", "METFORMIN HYDROCHLORIDE EXTENDED RELEASE TABLETS", "METFORMIN 1000 MILLIGRAM TABLETS", "GLICLAZIDE METFORMIN", "COMPETACT PIOGLITAZONE HYDROCHLORIDE METFORMIN HYDROCHLORIDE", "ARROW METFORMIN", "DIANBEN 850 MG METFORMIN", "METFORMIN HYDROCHLORIDE EXTENDED RELEASE TABLETS 750 MG PUREPAC", "METFORMIN BASICS 850MG", "METFORMIN NGX METFORMIN UNKNOWN 500MG", "METFORMIN 500MG TABLETS EMCURE TEVA", "GLYBURIDE METFORMIN HYDROCHL", "METFORMIN METFORMIN 1000 MG", "METFORMIN METFORMIN 1000 MG", "ORABET NGX METFORMIN UNKNOWN", "METFORMIN HYDROCHLORIDE ER TABLETS 500MG SUN SUN", "ACTOPLUS MEMT METFORMIN HYDROCHLORIDE PIOGLITAZONE HYDROCHLORIDE", "METFORMIN HYDROCHLORIDE AND VILDAGLIPTIN", "METFORMIN TABLET 500MG", "STAGID STAGID METFORMIN EMBONATE", "TAB METFORMIN HCL", "METFORMINE ALTER", "GLUCOPHAGE AND METFORMIN", "METFORMIN MANUFACTURER UNKNOWN", "METFORMIN HYDROCHLOLRIDE METFORMIN HYDROCHLORIDE", "METFORMIN HCL METFORMIN HCL UNKNOWN", "DIABEX XR METFORMIN", "DIABEX XR METFORMIN 500 MG", "GLUCOPHAGE METFORMIN 500 MILLIGRAM METFORMIN", "METFORMIN 500MG TABLET", "METFORMIN METFORMIN 850 MG", "GLIBENCLAMIDE METFORMIN CLIBENCLAMIDE METFORMIN", "METFORMIN HYDROCHLORIDE TABLET 500MG 850MG 1000MG METFORMIN", "METFORMIN HCL ROSIGLITAZONE", "METFORMINEHYDROCHLORIDE GLUCOPHAGE", "METFORMIN HCL ER 500MG", "METFORMIN SANDOZ NGX METFORMIN TABLET 850MG", "METFORMIN 850 METFORMIN 2 5 DF", "NU METFORMIN", "METFORMIN HCL XR METFORMIN", "DIANBEN DIANBEN METFORMIN HCL NOT SPECIFIED", "GENERIC METFORMIN METFORMIN XR", "VARIOUS MANUFACTURE NAME METFORMIN 500MG REGULAR EXP", "GLIPIZIDE W METFORMIN", "JANUMET METFORMIN 00082701", "GLIFAGE XR METFORMIN CHLORIDE METFORMIN HYDROCHLORIDE", "SUGUAN M METFORMIN HYDROCHLORIDE GLIBENCLAMIDE", "METFORMIN HYDROCHLORIDE METFORMIN HYDROCHLORIDE UNSPECIFIED", "METFORMIN HYDROCHLOROTHIAZIDE", "METFORMIN TABLETS", "METFORMIN GLIFAGE XR", "METFORMIN DURA", "METFORMIN HCL PCH", "METFORMIN ALPHARMA", "METFORMIN METFORMIN 000182701", "METFORMIN ARROW R 1000 MG", "METFORMINE 1000", "METFORMIN METFORMIN 500MG", "SUGUAN M METFORMIN HYDROCHLORIDE GLIBENCLAMIDE METFORMIN HYDROCHLO", "METFORMIN MRTFORMIN", "METFORMIN METPORMIN", "METFORMINA 00082701", "METFORMIN 500 MILLIGRAM TABLETS", "COMPETACT PIOGLITAZONE METFORMIN HYDROCHLORIDE", "METFORMINE HYDROCHLORIDE", "METFORMIN 6500 MG UNKNOWN MFR", "METFORMIN UNKNOWN STRENGTHS", "MELBIN METFORMIN HYDROCHLORIDE AMBROXOL HYDROCHLORIDE", "RIVA METFORMIN", "METFORMIN AND SITAGLIPTIN", "SITAGLIPTIN W METFORMIN HYDROCHLORIDE PILL", "METFORMIN 1000 MG MFR UNKNOWN", "METFORMIN MFR UNKNOWN", "METFORMIN 1000 MG MFR UNKNOWN", "METFORMIN HYDROCHLORIDE METFOMRIN HYDROCHLORIDE", "GLUCOPHAGE METFORMIN", "METFORMIN 850 MG TABLETS UNK UNK", "METFORMIN GLYB", "GLIMADAY GLIMEPIRIDE METFORMIN", "GEN METFORMIN", "3M METFORMIN", "JUFORMIN 500 MG METFORMIN", "JANUMET SITAGLIPTIN METFORMIN HYDROCHLORIDE", "METFORMIN HYDROCHLORIDE EXTENDED RELEASE METFORMIN HYDROCHLORIDE EXTE", "GLUCOPHAGE METFORMIN 500 MILLIGRAM METFORMIN", "MERFOMRIN MERFORMIN TABLET METFORMIN", "METFORMIN METFORMIN 1000 MG", "ACTOPLUS MET METFORMIN PIOGLITAZONE HYDROCHLORIDE", "METFORMIN HYDROCHLORIDE EXTENDED RELEASE WATSON LABORATORIES", "METFORMIN HYDROCHLORIDE AND GLYBURIDE", "METFORMIN XL 500 TWICE DAILY 500MG 2 TIMES A DAY", "METFORMIN NGX METFORMIN FILM COATED TABLET", "STAGID METFORMIN EMBONATE", "METFORMIN BIOGARAN", "GLIPIZIDE METFORMIN METFORMIN HYDROCHLORIDE GLIPIZIDE", "METFORMINE RANBAXY 500MG COMPRIME PELLICULE", "METFORMIN ER 500MG", "METFORMIN ZYDUS PHARMACEUTICALS", "METFORMIN EXTENDED RELEASE METFORMIN HYDROCHLORIDE", "METFORMIN HCI METFORMIN HYDROCHLORIDE TABLETS", "METFORMIN 1G", "JANUMET METFORMIN HYDROCHLORIDE SITAGLIPTIN PHOSPHATE TABLET", "VELMETIA METFORMIN HYDROCHLORIDE SITAGLIPTIN PHOSPHATE", "MEDET METFORMIN HYDROCHLORIDE TABLET", "METFORMIN HEXAL NGX", "MEDET METFORMIN HYDROCHLORIDE METFORMIN HYDROCHLORIDE", "VELMETIA SITAGLIPTIN METFORMIN ORAL ANTIDIABETICS", "COMPETACT PIOGLITAXONE HYDROCHLORIDE METFORMIN HYDROCHLORIDE", "METFORMIN AVANDIA", "ACTOSE PLUS METFORMIN", "METFORMIN HYDROCHLORIDE WATSON LABORATORIES", "METFORMIN HYDROCHLORIDE PIOGLITAZONE HYDROCHLORIDE", "METFORMIN GLUCOPHAGE", "D B I AP METFORMINA", "FORTAMET ER METFORMIN HYDROCHLORIDE", "MELBIN METOFORMIN HYDROCHLORIDE METFORMIN HYDROCHLORIDE", "MERFORMIN HYDROCHLORIDE METFORMIN HYDROCHLORIDE", "METFORMIN HEXAL METFORMIN HYDROCHLORIDE", "METFORMIN UNKNOWN MFR", "GLIFAGE XR METFORMIN HYDROCHLORIDE", "METFORMIN HYDROCHLORIDE GLUCOPHAGE SR", "RATIO METFORMIN METFORMIN HYDROCHLORIDE", "GLIMADAY GLIMEPIRIDE METFORMIN TABLET", "GLIPIZIDE METFORMIN METFORMIN GLIBENCLAMIDE", "METFORMIN ER 500MG TEVA", "METFORMIN AVANDIA COMBINATION", "GLUMETZA GLUMETZA METFORMIN HCL 500 MG NOT SPECIFIED", "GLIBENCLAMIDE METFORMIN GLIBENCLAMIDE METFORMIN", "METFORMIN HYDROCHLORIDE MEDET", "METFORMIN METFORMIN HYDROCHLORIDE METFORMIN HYDROCHLORIDE", "METFORMAIN METFORMIN HYDROCHLORIDE", "METFORMINE HCL PCH METFORMIN HYDROCHLORIDE TABLET 500 MG", "METFORMIN HCL PIOGLITAZONE HCL", "METFORMIN HYDROCHLORIDE GLUCOPHAGE SR", "METFORMIN DIABEX", "METFORMIN HPL", "D B I AP METFORMINA", "METFORMAX METFORMIN HYDROCHLORIDE", "METFORMIN VILDAGLIPTIN", "METFORMIN 1A PHARMA NGX", "GLUCOPHAGE METFORMIN 850 MILLIGRAM METFORMIN", "AVANDIA METFORMIN", "GLIBURIDE METFORMIN", "SANDOZ METFORMIN FC", "JANUMET METFORMIN SITAGLIPTIN", "METFORMIN SANDOZ NGX", "METFORMIN ER 500 MG SUNP", "VELMETIA METFORMIN HYDROCHLORIDE SITAGLIPTIN", "METFORMIN FILM COATED TABLET", "STAGIN METFORMIN EMBONATE", "DIFORMIN RETARD METFORMIN HYCROCHLORIDE", "METFORMIN PIOGLITAZONE METFORMIN PIOGLITAZONE", "METFORMIN 2G", "METFORMIN HYDROCHLORIDE 500 MG TABLETS METFORMIN HYDROCHLORIDE METF", "GLUCOPHAGE SR METFORMIN HYDROCHLORIDE", "METFORMIN 00082702 METFORMIN", "METFORMIN AND ROSIGLITAZONE", "METFORMIN GENERIC", "GYLBURIDE METFORMIN GLIBENCLAMIDE METFORMIN HYDROCHLORIDE", "METFORMIN TEVA", "METFORMIN COX", "METFORMIN HYDROCHLORIDE METFORMIN HYDROCHLORIDE METFORMIN HY", "GLUCOPHAGE 0008702 METFORMIN HYDROCHLORIDE TABLET", "METFORMIN HYDROCHLORIDE W SITAGLIPTIN", "METFORMIN HYDROCHLORIDE VILDAGLIPTIN", "METFORMIN METFORMIN ONGOING", "METFORMIN ER METFORMIM HYDROCHLORIDE", "GLUCOVANCE GLIBENCLAIMIDE METFORMIN HYDROXHLORIDE", "METFORMIN 500MILLIGRAM TABLETS", "METFORMIN UNKNOWN", "METFORMIN HEXAL", "METFORMIN RATIOPHARM", "METFORMIN METFORM 1000 MILLIGRAM METFORMIN", "VILDAGLIPTIN METFORMIN", "GLIMEPIRIDE METFORMIN", "METFORMIN HYDROCHLORIDE TABLET METFORMIN HYDROCHLORIDE TABLETS", "METFORMIN SITAGLIPTIN", "METFORMIN HYDROCHLORIDE METFORMIN HYDROCHLORIDE TABLETS METFORMIN", "GLIBURIDE METFORMIN HYDROCHLORIDE GLIBENCLAMIDE METFORMIN HYDROCHLOR", "GLIMEPIRIDE W METFORMIN", "METFORMIN HYDROCHLORIDE SANDOZ NGX", "METFORMIN METFORMIN 1000 MILLIGRAM METFORMIN", "GLUCOFORMIN METFORMIN METFORMIN HYDROCHLORIDE", "JANUMET SITAGLIPTIN METFORMIN", "GLUCOPHAGE METFORMIN FORMULATION UNKNOWN", "METFORMIN 1000MG TABLETS ZYDUS", "METFORMIN HYDROCHLORIDE TABLET METFORMIN HYDROCHLORIDE TABLETS", "METFORMIN XR 500 MG TABLETS TEVA", "METFORMIN GLIMEPIRIDE", "METFORMIN HYDROCHLORIDE NGX", "METFORMIN MEDA", "METFORMINA BLUEFISH", "METFORMINA CLORIDRATO", "GLIBENCLAMIDE METFORMIN HYDROCHLORIDE", "GLUCOPHAGE METFORMIN HYDROCHLORIDE TABLET 500MG", "METFORM METFORMIN HYDROCHLORIDE", "METFORMIN 0082702 METFORMIN HYDROCHLORIDE", "METFORMIN AUROBINDO FILM COATED TABLET", "COMPETACT PIOGLITAZONE HYDROCHLORIDE METFORMIN", "METFORMIN METFORMII METFORMIN", "BLINDED METFORMIN HCL XR", "METFORMIN HCL XR", "METFORMIN HYDROCHLORIDE SITAGLIPTIN PHOSPHATE", "METFORMINE HYDROCHLORIDE NGX", "METFORMIN NO PREF NAME", "METFORMINE METFORMIN HYDROCHLORIDE METFORMIN HYDROCHLORIDE", "METFORMINA WINTHROP", "DIABETEX METFORMIN HYDROCHLORIDE METFORMIN HYDROCHLORIDE", "JANUMET METFORMIN HC1 SITAGLIPTIN PHOSPHATE", "METFORMIN HYDROCHLORDIE UNKNOWN", "COMPETACT PIOGLITAZONE HYDROCHLORDIDE METFORMIN HYDROCHLORIDE", "METFORAL METFORAL METFORMIN HCL NOT SPECIFIED", "METFORMIN 500MG TABLETS ZYDUS", "PIOGLITAZONE HYDROCHI ORIDE METFORMIN HYDROCHLORIDE", "METFORMINUM", "GLUCOVANCE GLYBURIDE AND METFORMIN HCL", "CO METFORMIN", "SITAGLIPTIN AND METFORMIN HYDROCHLORIDE", "JANUMET SITAGLIPTIN METFORMIN", "METFORMIN METFORMIN TABLETS METFORMIN", "GLIPIZIDE W METFORMIN HYDROCHLORIDE", "GLUCOPHAGE XR METFORMIN HYDROCHLORIDE METFORMIN HYDROCHLORIDE", "PIOGLITAZONE HYDROCHLORIDE METFORMIN HYDROCHLORIDE PIOGLITAZONE HYDROC", "METFORMIN 500MG GENERIC", "METFORMIN HYDROCHLORIDE METFORMIN HYDROCHLORIDE 500 MILLIGRAM TABL", "ROXA METFORMIN", "VELMETIA METFORMIN SITAGLIPTIN", "METFORMNI METFORMIN", "PIOGLITAZONE HYDROCHLORIDE AND METFORMIN HYDROCHLORIDE", "METFORMIN ANTIDEPRESSANTS", "GLUCOFORMIN METFORMIN HYDROCHLORIDE METFORMIN HYDROCHLORIDE", "METFORMIN PFIZER", "GLUCOVANCE 01182201 GLIBENCLAMIDE METFORMIN HYDROCHLRIDE", "GLUCOPHAGE XR METFORMIN HYDROCHLORIDE METFORM IN HYDROCHLORIDE", "METFORMIN 850MG TABLET", "METFORMIN METFORMIN 500 MILLIGRAM METFORMIN", "METFORMIN HYDROCHLORIDE SITAGLIPTIN JANUMET", "GLUCOVANCE 01182201 GLIBENCLAMIDE METFORMIN HYDROCHLORIDE", "METFORMIN HYDROCHLORIDE W PIOGLITAZONE HYDROCHLORIDE", "GLUCOPHAGE METFORMIN METFORMTN", "METFORMIN 1000MG TABLET", "METFORMIN ER METFORMIN HYDROCHLORIDE TABLETS", "METFORMIN 500MG XR 2 DAILY", "METFORMIN 4000MG TABLET", "METFORMIN 1000 MG", "METFORMIN VILDAGLIPTIN", "SITAGLIPTIN METFORMIN HYDROCHLORIDE", "METFORMIN 500 MG FILM COATED TABLETS", "PIOGLITAZONE METFORMIN METFORMIN HYDROCHLORIDE PIOGLITAZONE HYDROCHL", "GLUCOVANCE GLYBURIDE METFORMIN GLYBURIDE METFORMIN", "METFORMIN 500MG TABLET ZYDUS", "GLYBURIDE METFORMIN HCL", "DIFORMIN RETARD METFORMIN HYDROCHLORIDE", "METFORMIN HCL EXTENDED RELEASE TABLETS", "METFORMIN METFORMIN 435 MILLIGRAM TABLETS METFORMIN", "METFORMIN GLUCOPHASE", "METFORMIN CON", "METFORMINE MERCK 00082701", "METFORMIN TABLET PFIZER", "GLYME M2 GLIMEPRIDE W METFORMIN GLIMEPRIDE W METFORMIN", "METFORMIN NON ROCHE", "GLUCOFAGE METFORMIN HYDROCHLORIDE", "METFORMIN NON ROCHE NON COMPARATOR", "METFORMIN METFORMIN 50 MILLIGRAM TABLETS", "GLUCOVAN GLIBENCLAMIDE METFORMIN HYDROCHLORIDE", "ACTOS MET METFORMIN HYDROCHLORIDE PIOGLITAZONE HYDROCHLORIDE", "METFORMIN HYDROCHLORIDE W PIOGLITAZONE HYDROC", "GLUKOFEN METFORMINE HCL", "METFORMINE HCL PCH", "METFORMIN METFORMIN 850 MILLIGRAM METFORMIN", "GLIFOR METFORMIN HCL", "EUCREAS METFORMIN VILDAGLIPTIN METFORMIN VILDAGLIPTIN", "METFORIN METFORMIN", "GLYBER METFORMIN", "SITAGLIPTIN PHOSPHATE METFORMIN HYDROCHLORIDE", "SITAGLIPTIN METFORMIN METFORMIN AND SITAGLIPTIN METFORMIN AND SITAG", "PIOGLITAZONE HYDROCHLORIDE METFORMIN HYDROCHLORIDE", "GLIFAGE METFORMIN 500 MILLIGRAM TABLETS METFORMIN", "METFORMIN HYDROCHLORIDE TABLETS 850 MG PUREPAC METFORMIN HYDROCHLO", "METFORMIN HYDROCHLORIDE VILDAGLIPTIN", "ACTOPULSE MET PIOGLITAZONE HYDROCHLORIDE METFORMIN HYDROCHLORIDE", "METFORMIN ACTAVIS", "METFORMIN ARROW", "STAGID METFORMIN EMBONATE METFORMIN EMBONATE", "METGLUCO METFORMIN HYDROCHLORIDE", "METFORMIN METFORIN HYDROCHLORIDE", "GLIFAGE METFORMIN", "METFORIN HCL METFORMIN HYDROCHLORIDE", "JANUMET METFORMIN AND SITAGLIPTIN", "DIABEX METFORMIN HYDROCHLORIDE METFORMIN HYROCHLORIDE", "GLYBURIDE AND METFORMIN HYDROCHL", "METFORMIN HYDRODCHLORIDE SITAGLIPTIN METFORMIN AND SITAGLIPTIN", "METOFRMIN METFORMIN", "METFORMIN MYLAN", "HYDROCHLORIDE METFORMIN HYDROCHLORIDE", "JANUMET METFORMIN SITAGLIPTIN METFORMIN SITAGLIPTIN", "METFORM HCL METFORMIN HYDROCHLORIDE", "EUCREAS METFORMIN VILDAGLIPTIN", "VILDAGLIPTIN METFORMIN HYDROCLORIDE", "METFORMINA CLOROIDRATO", "METFORMINE TABLET 500MG", "JANUMET METFORMIN HYDROCHLORIDE SITAGLITIN PHOSPHATE TABLETS", "METFORMIN 1000 MG FILMTABL 1A PHARMA", "GLUCOPHAGE METFORMIN HYDROCHLORIDE 850 MILLIGRAM TABLETS METFORM", "GALVUS MET 50 850 VILDAGLIPTIN 50 MG METFORMIN 850 MG", "GLYBURIDE METFORMIN METFORMIN HYDROCHLORIDE GLIBENCLAMIDE", "TEVA METFORMIN", "METFORMIN 1000MG TABLET ZYDUS", "SAXAGLIPTIN METFORMIN HCL", "PRO METFORMIN", "METGLUCO METFORMIN HYDROCHLORIDE TABLET", "METFORMIN MIXTURE", "JUANUMET METFORMIN HYDROCHLORIDE SITAGLIPTIN PHOSPHATE", "METFORMIN 2 TABS", "METFORMIN HYDROCHLORIDE UNK", "JANUMET METFORMIN HYDROCHLORIDE SITAGLIPTIN PHOSPHATE METFORMIN HY", "METFORMIN METFORMIN 850 MG METFORMIN", "METFORMIN W SITAGLIPTIN", "METFORMIN W SITAGLIPTIN", "GLIMEPIRIDE METFORMIN HYDROCHLORIDE", "JANUMET METFORMIN", "METFORMIN SITAGLIPTIN METFORMIN SITAGLIPTIN", "COMPETACT PIOGLITAZONE HYDROCHLORIDE METFORMIN HDROCHLORIDE", "METFORMIN HYDROCHLORIDE W VILDAGLIPTIN", "METFORMIN METFORMIN 10012613 DIABETES MELLITUS NON INSULIN DEPEND", "JANUMET WITH METFORMIN", "METFORMIN COMBINATION", "JANUMET METFORMIN HYDROCLORIDE SITAGLIPTIN PHOSPHATE", "JANUMET METFORMIN", "SITAGLIPTIN METFORMIN 50MG 1000MG", "SITAGLIPTIN METFORMIN 50MG 500MG", "JANUMET METFORMIN HYDROCHLORIDE SITAGLIPTIN PHOSPHATE TABLETS", "GLYBURIDE METFORMIN METFORMIN HYDROCHLORIDE GLIBENCLAMIDE TABLETS", "GLIMEPIRIDE METFORMIN HYDROCHLORIDE", "BI EUGLUCON METFORMIN GLIBENCLAMIDE TABLETS METFORMIN GLIBENCLA", "GLUCOPHAGE METFORMIN HYDROCHLORIDE 1000 MILLIGRAM TABLET METFORM", "METFORMIN AL", "GLUMETZA METFORMIN", "GLUPA METFORMIN HYDROCHLORIDE", "JENUVIA METFORMIN", "GLYBURIDE WITH METFORMIN SIL NORBORAL", "METFORMIN SACHETS", "METFORMIN GLYBURIDE METFORMIN", "METFORMINA METFORMIN HYDROCHLORIDE METFORMIN HYDROCHLORIDE", "METFORM METFORMIN METFORMIN", "STAGIG METFORMIN EMBONATE METFORMIN EMBONATE", "METFORMIN METFORMIN HYDROCHLORIDE 500 MG", "GLUCOPHAGE METFORMIN METFORMIN HYDROCHLORIDE", "METFORMINA 00082702", "JANUMET METFORMIN HYDROCHLORIDE SITAGLIPTIN PHOSPHATE MONOHYDRATE", "KOMBIGLYZE METFORMIN HYDROCHLORIDE SAXAGLIPTIN HYDROCHLORIDE", "METOFORMIN METFORMIN HYDROCHLORIDE", "METFORMIN METFORMIN TABLET METFORMIN", "METFORMIN NO DSE PRODUCT METFORMIN METFORMIN", "GLUCORAN METFORMIN HYDROCHLORIDE", "METFORMIN UNKNWON", "METFORMIN ACTAVIS", "GLIFAGE XR METFORMIN", "METFORMIN METFORIN", "METFORMIN HEXAL SANDOZ", "SITAGLIPTINE METFORMINE", "JANUMET METFORMIN HYDROCHLORIDE SITAGLIPTIN PHOSPHATE 09 29 2009 TO", "GLYBURIDE METFORMIN METFORMIN HYDROCHLORIDE", "METFORMIN SITAGLIPTIN METFORMIN AND SITAGLIPTIN METFORMIN AND SITAGLI", "COMPETACT PIOGLITAZONE HYDROCHLORIDE METFORMIN HYDROCHLORIDE 15", "METFORMIN XR GLUCOPHAGE XR", "METFORMIN HYDROCHLORID", "METFORMINE HYDROCHLORDE", "METFORMINA GLIBENCLAMIDA", "SITAGLIPTIN METFORMIN 50 500", "SITAGLIPTIN METFORMIN HCL", "GALENIC GLIBENCLAMIDE METFORMIN", "METGLUCO METFORMIN HYDROCHLORIDE METFORMIN HYDROCHLORIDE", "METFORMIN METFORMN", "METFORMIN B", "METFORMIN HCL METFORMIN HYDROCHLORIDE UNKNOWN", "GLYBURIDE W METFORMIN HYDROCHLORID", "METFORMIN METFORMIN 500 MILLIGRAM TABLETS", "GLYBURIDE METFORMIN GLIBOMET", "DIANBEN METFORMIN HYDROCHLORIDE TABLET", "GLYCORAN METFORMIN HYDROCHLORIDE TABLET 250 MG", "AVANDA METFORMIN", "METFORMIN GLUCOPHAGE HYDROCHLORIDE", "TAB METGLUCO METFORMIN", "MERFORMIN METFORMIN", "METFORMIN WEIFA", "METFORMIN SITAGLIPTIN", "METFORMIN GLIMIPERIDE", "STAGMID METFORMIN EMBONATE", "VILDAGLIPTIN METFORMIN", "GLYBERIDE METFORMIN", "METACT PIOGLOITAZONE HYDROCHLORIDE METFORMIN HYDROCHLORIDE", "GLYCERIDE METFORMIN", "METFORMIN 3 A DAY 500 MG", "GLYBIRIDE METFORMIN", "METACT PIOGLITAZONE HYDROCHLORIDE METFORMIN HYDROCHLORIDE", "GLUMETZA METFORMIN HYDROCHLORIDE EXTENDED RELEASE TABLET", "METFONORM METFORMIN HYDROCHLORIDE", "GLIMEPIRIDE METFORMIN HCL", "GLUCOPHAGE SR METFORMIN", "GLYBURIDE W METFORMIN HYDROCHLORIDE TABLETS", "METFORMINE MYLAN", "METFORMINA GLIBENCLAMIDE SANDOZ", "BLINDED SITAGLIPTIN PHOSPHATE METFORMIN HYDROCHLORIDE", "AVANDAMET METFORMIN HYDROCHLORIDE ROSIGLITAZONE MALEATE TABLETS", "METFORMIN HCL TABS", "METFORMIN XR METFORMIN HYDROCHLORIDE 500 TABLET", "METFORMIN W SAXAGLIPTIN", "EUCREAS METFORMIN HYDROCHLORIDE VILDAGLIPTIN", "METFORMIN METFORMIN HYDROCHLORIDE 1000 MILLIGRAM", "METFORMIN HYDROCHLORIDE UNKNOWN", "SITAGLIPTIN PHOSPHATE METFORMIN HYDROCHLORIDE", "ER METFORMIN", "METACT COMBINATION TABLETS LD PIOGLITAZONE HYDROCHLORIDE METFORMIN HYDROCHLORIDE", "ROSIGLITAZONE MALEATE AND METFORMIN HYDROCHLORIDE", "METFORMIN WINTHROP", "COMPETACT PIOGLITAZONE HYDROCHLORIDE METFORMIN HYDROCHLORIDE PIOGLITAZONE HYDROCHLORIDE 15 MG METFORMIN HYDROCHLORIDE 850 MG 1 IN 1 D", "ACTOPLUS MET PIOGLITAZONE HYDROCHLORIDE METFORMIN HYDROCHLORIDE", "ROSIGLITAZONE METFORMIN", "ROSIGLITAZONE METFORMIN METFORMIN W ROSIGLITAZONE", "GLYBURIDE W METFORMIN", "GLYBURIDE W METFORMIN HYDROCHLORIDE GLIBENCLAMIDE METFORMIN HYDROCHLORIDE", "METFORMINHYDROCHLORID", "METFORMIN 2", "METFORMIN HYDROCHLORIDE SANDOZ", "METFORMIN METFORMIN HCL", "GLYBURIDE METFORMIN", "GLIMEPERIDINE METFORMIN", "METFORMIN ALPHARMA METFORMIN HYDROCHLORIDE", "BLINDED METFORMIN", "DIFORMIN RETARD METFORMIN 500 MILLIGRAM PROLONGED RELEASE TABLET", "SITAGLIPTIN PHOSPHATE METFORMIN HCL", "METFORMIN HCL TABS 500MG", "METFORMIN NON AZ PRODUCT", "GLUCOPHAGE METFORMIN HCL", "MEFORMIN METFORMIN HYDROCHLORIDE", "VILDAGLIPTIN METFORMIN HYDROCHLORIDE", "METFORMIN HYDROCHLORIDE W VILDAGLIPTIN DRUG USED IN DIABETES", "METFORMINE BASE", "DOM METFORMIN", "MERCK METFORMIN", "METFORMIN HYDROCHLORIDE METFORMINA", "METFORMIN NON ABBOTT", "METFORMIN AL METFORMIN HYDROCHLORIDE", "METFORMIN HCT", "GLUCOPHAGE METFORMIN HYDROCHLORIDE", "METFORMIN METFORMIN SUSTAINED RELEASE TABLET", "METFORMIN UNSPECIFIED BRANDNAME", "MYLAN METFORMIN", "METFORMINA TEVA", "METFORMIN HCL METFORMIN HYDROCHLORIDE TABLET", "METFORMINE TABLET 1000MG", "METFORMIN HYDROCHLORIDE TABLETS METFORMIN UNKNOWN METFORMIN", "GLUCOVANCE METFORMIN HYDROCHLORIDE GLIBENCLAMIDE", "METFORMINA MYLAN", "METFORALMILLE METFORMIN HYDROCHLORIDE", "METFORMIN METFORMIN 850", "METFORMIN HYDROCHLORIDE TABLETS", "METFORMIN GLITAZONE", "METFORMIN 500", "RAN METFORMIN", "ROSIGLITAZONE MALEATE METFORMIN HYDROCHLORIDE", "GLIPIZIDE METFORMIN HYDROCHLORIDE", "METFORMIN SITAGLIPTIN PHOSPHATE", "METFORMIN HYDROCHLORIDE EXTENDED RELEASE TABLETS 500 MG PUREPAC METFORMIN HYDROCHLORIDE", "METFORMINE TABLET 500MG", "EUCREAS METFORMIN HYDROCHLORIDE METFORMIN HYDROCHLORIDE", "METFORMIN 500MG UNKNOWN", "ACTOPLUS MET PIOGLITAZONE HYDROCHLORIDE METFORMIN HYDROCHLORIDE", "METFORMIN HC1", "METFORMIN HYDROCHLORIDE EXTENDED RELEASE TABLETS 750 MG PUREPAC METFORMIN HYDROCHLORIDE", "METFORMIN 500MG PO BID", "METFORMIN METFORMIN 500 MILLIGRAM TABLET METFORMIN", "GLUCORIDE METFORMIN", "METFORMIN SR 500 MG", "METFORMINA SANDOZ", "METFORMIN 1 000 MG", "METFORMIN TEVA", "METFORMIN HYDROCHLORIDE SAXAGLIPTIN", "METFORMIN EX", "METFORMIN NON ABBOTT", "GLYBERIDE METFORMIN NON ABBOTT", "METFORMIN 1A PHARMA", "METFORMINA ACTAVIS", "METFORMIN HYDROCHLORIDE EXTENDED RELEASE TABL ETS", "GLYBURIDE METFORMIN HYDROCHLORIDE", "METFORMIN HYDROCHLORIDE TABLETS 1000MG", "METFORMIN SITAGLIPTIN METFORMIN AND SITAGLIPTIN", "METFORMIN SITAGLIPTIN UNKNOWN", "METFORMINE LP", "METFORMIN FUROSEMIDE", "METEX METFORMIN HYDROCHLORIDE", "METFORMIN HCL 500MG TABLETS METFORMIN HYDROCHLORIDE TABLET 500MG HYDROCHLORIDE", "METGLUCO METFORMIN HYDROCHLORIDE", "GLIPIZIDE METFORMIN HCL", "GLYBL METFORMIN GLIBOMET", "METFORMIN HYDROCHLORIDE TABLETS METFORMIN UNKNOWN METFORMIN", "METFORMIN AL 850", "METFORMIN HCL TABLETS", "METFORMIN HYDROCHLORIDE METFORMIN UNKNOWN", "MEFORMIN HYDROCHLORIDE METFORMIN HYDROCHLORIDE", "METFORMIN HYDROCHLORIDE METFORMIN HYDROCHLORIDE METFORMIN HYDROCHLORIDE", "KOMBIGLYZE METFORMIN HYDROCHLORIDE SAXAGLIPTIN HYDROCHLORIDE", "EUCREAS METFORMIN HYDROCHLORIDE VILDAGLIPTIN TABLET METFORMIN HYDROCHLORIDE VILDAGLIPTIN", "EUREAS 50 MG 1000 MG METFORMIN HYDROCHLORIDE VILDAGLIPTIN COATED TABLET METFORMIN HYDROCHLORIDE VILDAGLIPTIN", "JANUMET METFORMIN HYDROCHLORIDE SITAGLIPTIN PHOSPHATE MOMOHYDRATE", "METFORMIN METFORMIN METFORMIN", "METRFORMIN METFORMIN", "GLIMEPIRIDE METFORMIN HYDROCHLORIDE", "METFORMIN 500 MG TABLETS", "GLUCOVANCE GLIBENCLAMIDE METFORMIN HYDROCHLORIDE", "METFORMINE PFIZER", "JANUMET SITAGLIPTIN METFORMIN SITAGLIPTIN METFORMIN", "GLUCOPHAGE METFORMIN HYDROCLORIDE", "JANUMET METFORMIN HYDROCHLIRDE SITGLIPTIN PHOSPHATE MONOHYDRATE", "METFORMIN METFORMIN METFORMIN", "METFORMIN METFORMIN METFORMIN", "GLUCOPHAGE METFORMIN HYDROCHLORIDE METFORMIN HYDROCHLORIDE", "T METFORMIN", "METFORMIN GENERICON", "METFORMINE METFORMINE METFORMINE", "METOFRMIN ER METFORMIN HYDROCHLORIDE", "METFORMIN 1500MG DAY", "JANUMAET METFORMIN HYDROCHLORIDE SITAGLIPTIN PHOSPHATE MONOHYDRATE", "GLYBURIDE AND METFORMIN HCL GLIBENCLAMIDE METFORMIN HYDROCHLORIDE", "GLUCOPHAGE METFORMIN HYDROCHLORIDE TABLET 500MG", "JANUMET METFORMIN HYDROCHLORIDE SITAGLIPTIN PHOPSHATE MONOHYDRATE", "GLOCOPHAGE METFORMIN HYDROCHLORIDE", "METFORMIN HCL METFORMIN HYDROCHLORIDE 1000 MILLIGRAM TABLETS", "SITAGLIPTINA METFORMINA", "GLIPIZIDE METFORMIN METAGLIP", "GLYBURIDE METFORMIN GLIBOMET", "METFORMIN HYDROCHLORIDE METFORMIN HYDROCHLORIDE METFORMIN HYDROCHLORIDE", "JANUMET SITAGLIPTIN PHOSPHATE METFORMIN HYDROCHLORIDE FILM COATED TABLET", "JANUMET SITAGLIPTIN PHOSPHATE METFORMIN HYDRLCHLORIDE FILM COATED TABLET 500 1000", "JANUMET SITAGLIPTIN PHOSPHATE METFORMIN HYDROCHLORIDE FILM COATED TABLET 50 MG 1000MG", "METFORMIN METFOMMIN", "SAXAGLIPTIN METFORMIN", "METFORMIN METFORMIN 1500 MILLIGRAM METFORMIN", "SLOW RELEASE METFORMIN", "GLIFAGE XR METFORMIN METFORMIN", "METFORALMILLE METFORMIN HYDROCHLORIDE METFORMIN HYDROCHLORIDE", "METFORMIN HYDROCHLORIDE METFORMIN HYDROCHLORIDE", "METFORMIN HYDROCHLORIDE GLUCOPHAGE", "GLIFAGE METFORMIN METFORMIN", "GLUCOPHAGE METFORMIN 1000 MG", "MK 9378 METFORMIN TABLET", "MK 9378 METFORMIN TABLET", "ML 9378 METFORMIN TABLET", "MK 9378 METFORMIN", "MK 9378 METFORMIN TABLET", "JANUMET SITAGLIPTIN PHOSPHATE METFORMIN HYDROCHLORIDE FILM COATED TABLET", "SAXAGLIPTIN HCL METFORMIN", "ER METFORMIN HYDROCHLORIDE", "GLUCOPHAGE METFORMIN HYDROCHLORIDE 50MG", "METFORMIN HCL 500MG", "METFORMIN HYDROCHLORIDE UNKNOWN METFORMIN HYDROCHLORIDE UNK UNKUNK", "METFORMIN PIOGLITAZONE METFORMIN", "METFORMINA 00082701", "PIOGLITAZONE HCL METFORMIN HCL", "METFORMIN HCL XR TABS 750 MG", "EUCREAS METFORMIN HYDROCLORIDE VALIDAGLIPTIN", "METFORMIN 850 MG", "METFORMINE METFORMIN HYDROCHLORIDE METFORMIN HYDROCHLORIDE", "METFORMIN TEVA 1000 MG", "METFORMIN ABZ", "METFORMINE TEVA 1000", "METFORMIN TEVA 500MG", "METFORMIN HYDROCHLORIDE ROSIGLITAZONE MALEATE", "METFORMION METFORMIN", "METFORMIN METFORMIN HYDROCHLORIDE", "METGLUCO METFORMIN HYDROCHLORIDE METFORMIN HYDROCHLORIDE", "GLYBURIDE METFORMIN HCL NOS", "METFORMIN HYDROCHLORIDE SITAGLIPTIN PHOSPHATE METFORMIN HYDROCHLORIDE W SITAGLIPTIN METFORMIN HYDROCHLORIDE SITAGLIPTIN", "METFORMIN HCL NON AZ DRUG", "METFORMINE ALFPHARA METFORMIN HYDROCHLORIDE METFORMIN", "METFORMIN FOR PCOS UNITHROID", "METFORMIN HIDROCHLORIDE", "METFORMINE ARROW", "JANUMET METFORMIN HYDROCHLORIDE SITAGLIPTIN PHOSPHAE MONOHYDRATE METFORMIN HYDRORIDE SITAGLIPTIN PHOSPHOSE MONOHYDRATE", "METFORMIN HCI METFORMIN HYDROCHLORIDE", "ONGLYZA METFORMIN", "METFORMIN 50", "METFORMIN ER METFORMIN HYDROCHLORIDE", "METFORMIN METFORMIN METFORMIN", "GLIBENCLAMIDE METFORMIN 2 5MG 500MG", "GLUCOPHAGE METFORMIN HYDROCHLORIDE", "METFORMIN SR 500MG", "METFORMIN HCL 1000", "METUGLUCO METFORMIN HYDROCHLORIDE", "METFORMIN A METFORMIN", "METFORMIN PLANTAGO OVATA", "METFORMINE SANDOZ", "METFORMIN HCL NON AZ PRODUCT", "METGLUCO METFORMIN HYDROCHLORIDE", "GLIBENCLAMIDE METFORMIN", "METFORMIN HCL METFORFIN HYDROCHLORIDE", "JANUMET 06902601 METFORMIN HYDROCHLORIDE SITAGLIPTIN PHOSPHATE", "METFORMIN METOFORMIN", "JANUMET 06535301 METFORMIN HYDROCHLORIDE SITAGLIPTIN PHOSPHATE MONOHYDRATE", "GLYBURIDE METFORMIN GLIBENCLAMIDE METFORMIN HYDROCHLORIDE", "ACTOPLUS MET METFORMIN HYDROCHLORIDE PIOGLITAZONE HYDROCHORIDE", "JANUMET 9METFORMIN HYDROCHLORIDE SITAGLIPTIN PHOSPHATE MONOHYDRATE", "MK 9373 METFORMIN TABLET", "GLUCOPHAGE METFORMIN HYDROCHLODIE", "METFORMIN METORMIN UNKNOWN", "METFORMIN HYDROCHLORIDE METFORMIN HYDROCHLORIDE METFORMIN HYDROCHLORIDE", "METFORMIN METFROMIN UNKNOWN", "GLUCOPHAGE METFORMIN HYDROCHLORIDE UNKNOWN", "METFORMIN HCL METFORMIN HYDROCHLORIDE UNKNOWN", "METFORM METFORMIN UNKNOWN", "METFORMIN METFORMIN 500 MILLIGRAM UNKNOWN", "METFORMINA SANDOZ 850 MG COMPRIMIDOS RECUBIERTOS", "PIOGLITAZONE METFORMIN", "METFORMIN HCL METFORMIN HYDROCHLORIDE 850 MILLIGRAM TABLETS", "METGLUCO METFORMIN", "METFORMIN HCL ER METFORMIN HYDROCHLORIDE", "GLUCOPHAGE METFORMIN UNKNOWN", "JANUMET SITAGLIPTIN PHOSPHATE METFORMIN HYDROCHLORIDE FILM COASTED TABLET", "METFORMIN HYDROCHLORIDE SITAGLIPTIN PHOSPHATE", "JANUMENT SITAGLIPTIN PHOSPHATE METFORMIN", "METFORMIN METFORMIN TABLET 1000 MG", "METFORMIN GLUCOPHAGE", "METFORMIN HYDROCHLORIDE METFORMIN HYDROCHLORIDE TABLET", "METFORMIN HCL ER", "GLUCOPHAGE METFORMIN HYDROCHLORIDE METFORMIN HYDROCHLORIDE", "METFORMIN HCL METFORMIN", "METFORMIN HYDROCHLORIDE AELLC", "METFORMIN HCL 1000MG TABLETS", "METFORMIN HYDROCHLORIDE GLUMETZA", "METFORMIN HCL METFORMIN HYDROCLORIDE", "ACTOPLUS MET METFORMIN HYDROCHLORIDE PIOGLITAZONE HYDROCHLORIDE", "ACTOPLUS MET METFORMIN HYDROCHLORIDE", "JANUMET 06535301 METFORMIN HYDROCHLORIDE SITAGLIPTIN PHOSPHATE MONOHYDRATE", "GLYBURIDE METFORMIN HYDROCHLORIDE", "JANUMET METFORMIN HYDROCHLORIDE SITAGLIPTIN PHOSPHAGE MONOHYDRATE", "COMPARATOR METFORMIN", "METFIN METFORMIN HYDROCHLORIDE FILM COATED TABLET", "METFORMINA TEVA 500MG", "BI 1356 METFORMIN", "D B I METFORMINA", "GLIPIZIDE METFORMIN METAGLIP", "EUCREAS VILDAGLIPTIN METFORMIN HYDROCHLORIDE", "GE METFORMIN", "METFORMIN 1G", "METFORMIN SR 500", "METFORMIN KERN PHARMA 850 MG", "METFORMIN HYDROCHLORIDE TABLETS 500MG", "SIOFOR 500 METFORMIN HYDROCHLORIDE", "METFORMIN RATIOPHARM 1000 MG FILMTABLETTEN", "METFORMIN GLUCOPHAGE XR 750 MG ORAL TABLET EXTENDED RELEASE", "METFOGAMMA METFORMIN HYDROCHLORIDE", "GLUCHPHAGE METFORMIN HYDROCHLORIDE", "GLUCOBAN GLIBENCLAMIDE METFORMIN", "METFIN METFORMIN HYDROCHLORIDE", "AMARYL METFORMIN HCL", "GLUCODOWN METFORMIN HCL", "ROSIGLITAZONE MALEATE METFORMIN HYDROCHLORIDE", "GLUCOVANCE METFORMIN", "METFORMIN HCL IR 500MG", "METFORMIN TAB ER", "METFORMIN HYDROCHLORIDE GLIPIZIDE", "METFORMIN TARTRATE", "GLIPIZIDE METFORMIN HCL", "GLICLAZIDE METFORMIN HYDROCHLORIDE", "METFORMIN 1000MG 1 TAB TWICE PER DAY", "JANUMET RISTFOR METFORMIN HYDROCHLORIDE SITAGLIPTIN PHOSPHATE MONOHYDRATE", "GLUCOLYTE METFORMIN HYDROCHLORIDE", "METFORMINE TEVA", "METFORMIN HCL 100", "METFORMIN PCH", "METFORMINA TEVA ITALIA 1000 MG", "METFORMINE TABET 850 MG", "METFORMIN ORIFARM", "METFORMIN ER GLUCOPHAGE XR", "METFORMIN HYDROCHLORIDE METFORMIN HYDROCHLORIDE METFORMIN HYDROCHLORIDE", "KOMBIGLYZE METFORMIN HYDROCHLORIDE SAXAGLIPIN HYDROCHLORIDE", "METFORMIN SITALGLIPTIN", "METFORMIN METFORMIN HYDRHCLORIDE", "METFORMIN UNKNOWN METFORMIN", "METFORMIN UNKOWN", "METFORMIN METFORMIN MEFTFORMIN", "GLUCOMET GLYBURIDE METFORMIN HYDROCHLORIDE", "METFORMIN METFORMIN UNKNOWN METFORMIN", "ZOMARIST METFORMIN HYDROCHLORIDE VILDAGLIPTIN", "METFORMIN METFORMIN TABLETS", "METFORMIN HYDROCHLORIDE ER TABLETS", "NELBIS METFORMIN HYDROCHLORIDE", "METFORMINE HYDROCHORIDE", "METFORMIN 100MG", "METFORMINA TEVA 500 MG", "METFORMIN SAXAGLIPTIN KOMBIGLYZE", "METFORMAX METFORMIN HYDROCHLORIDE 850 MILLIGRAM TABLET METFORMIN HYDROCHLORIDE", "METFORMINE MYLAN 850MG", "METFORMIN HCL BMS", "METFORMIN SAXAGLIPTIN", "SIOFOR METFORMIN", "NR METFORMIN", "DIAFORMIN METFORMIN HYDROCHLORIDE", "METFORMINE BIOGARAN 500 MG", "METFORMIN HCI ER", "METFORMIN HYDROCHLORIDE TABLETS USP", "METFORMIN HCL ER 500 MG", "METFORMIN CHLORIDRATE", "METFORMIN HCL 500MG TABLETS", "METFORMIN W PIOGLITAZONE", "ACOTOS METFORMIN", "KOMBIGLYZE W METFORMIN", "JANUMET SITAGLIIPTIN METFORMIN", "METFORMINE TABLET 850MG", "METFORMIN PLUS GLYBURIDE", "LINAGLIPTIN W METFORMIN", "METFORMIN EYR", "METFORMIN METFORMIN HYDROCHLORIDE TABLET", "METFORMIN COMBO MEDICATION", "METFORMIN HYDROCHLORIDE TABLETS USP 500 MG METFORMIN TABLET", "GLICLAZIDE METFORMIN", "GLUCOMIN METFORMIN", "METFORMINE HCL MERCK", "METFORMIN DOC GENERICI", "METFORMIN TABLETTEN", "GLYBURIDE METFORMIN 2 5MG 500MG HERITAGE PHARMACEUTICALS INC", "GLYCON METFORMIN HYDROCHLORIDE", "METFORMIN HCL METFORMIN HYDROCHLOIRIDE", "METFORMINE GLUCOPHAGE", "METFORMIN 750MG EXTENDED RELEASE TABLET", "METFORMIN 1000 MG KG", "METFORMINE NON AZ PRODUCT", "METFORMINE TEVA 850 MG", "METFORMIN NON AZ", "GLIBENCLAMIDE METFORMIN SIL NORBORAL", "METFORMIN HYDROCHLORIDE NON AZ PRODUCT", "MINT METFORMIN", "METFORMINE TEVA 500 MG", "GLYBURIDE AND METFORMIN COMBINATION", "METFORMIN HYDDROCHLORIDE METFORMIN HYDROCHLORIDE METFORMIN HYDROCHLORIDE", "METFORMIN HCL POWDER", "METFORMIN HCL ER OSM", "METFORMIN MEPHA 500 MG", "LINAGLIPTIN METFORMIN HYDROCHLORIDE", "METFORMIN A L", "METFORMIN UNKNOWN UNKNOWN", "METFORMIN HCL METFORMIN HYDROCHLORIDE", "METFORMIN HCL ER METORMIN HYDROCHLORIDE TABLETS", "METFORMIN METFORMIN CHLOROPHENOXYACETATE METFORMIN EMBONATE METFORMIN HYDROCHLORIDE", "METFORMIN EQL PHARMA 500 MG FILM COATED TABLET", "METFORMIN EQL PHARMA", "METFORMIN ISOMED", "METFORMIN HCL PFIZER", "METFORMIN GLIPTIN KOMBINATIONSPRAPARAT 50 1000 MG", "METFORMIN HCL OXYCODONE HYDROCHLORIDE", "METFORMIN HCL METFORMIN HYDROCHLORIDE", "METFORMIN HCL METFORMIN HYDROCHLORIDE TABLETS", "METFORMIN METFORMIN UKNOWN", "GLUCOPHAGE METFORMIN TABLETS", "METFORMIN METFORMIN UNKNOWN UNKNOWN", "METFORMIN XR METFORMIN XR METFORMIN XR", "METFORMIN CLORIDRATE", "GLUMEXA METFORMIN", "GALVUS MET METFORMIN", "METFORMIN XR METFORMIN METFORMIN", "METFORMIN METFORMIN UNKNWN UNKNOWN", "JANUMET METFORMIN HYDROCHLORIDE SITAGLIPTIN PHOSPHATE MONOHYDRATE", "JAMP METFORMIN", "METFORAL METFORMIN HYDROCHLORIDE", "METFORMIN HYDROCHLORIDE SITAGLIPTIN PHOSPHATE MONOHYDRATE", "METFORMIN 1000MG METFORMIN UNKNOWN 1000MG", "METFORMIN W VILDAGLIPTIN", "METFORMIN METOFRMIN", "METFORMIN NON AZ PRODUCT", "SITAGLIPTIN METFORMIN METFORMIN HYDROCHLORIDE SITAGLIPTIN PHOSPHATE", "METFORMINE NON AZ PRODUCT", "METFORMINE 850 MYLAN", "METFORMIN HCL METFORMIN HYDOCHLRODIE", "METFORMIN HYDROCHLORIDE METFORMIN MHYDROCHLORIDE METFORMIN", "GLUCOPHAGE METFORMIN HYDROCHLORIDE COATED TABLET", "METFORMIN HCL METFORMIN HYDROCHLORIDE", "METFORMIN HYDROCHLORIDE NON AZ DRUG", "METFORMIN HYDROCHLORIDE ROSIGLITAZONE MALEATE", "METFORMIN NON AZ DRUG", "METFORMIN HCL METFORMIN HYDROCHLORIDE TABLETS", "METFORMIN HYDROCHLORIDE GENERIC DRUG", "METFORMIN HCL METFORMIN HYDROCHLORIDE UNKNOWN", "METFORMIN TABLETS BP 500MG", "JENTADUETO LINAGLIPTIN METFORMIN ORAL ANTIDIABETICS", "METFORMIN HYDROCHLORIDE 100MG ML PL00427 0139", "METFORMIN HYDROCHLORIDE GLYBURIDE", "METFORMIN AUROBINDO FILM COATED TABLETS 850MG", "METFORGAMMA METFORMIN HYDROCHLORIDE", "MEFORAL 00082701 METFORMIN", "METFORMIN HCL 1000MG TABLETS", "METFORMIN ARROW 1000MG", "METFORMIN ARROW FILM COATED TABLET", "METFORMIN AUROBINDO FILM COATED TABLETS 500MG", "SITAGLIPINA AND METFORMIN", "METFORMIN HYDROCHLORIDE TABLETS USP 500 MG", "METFORMIN METFORIMIN", "GLUMETZA METFORMIN HYDROCLORIDE", "METFORMIN HCL ER TABLETS USP 500 MG MANUFACTURED BY APOTEC", "METFORMIN HYDROCHLORIDE TABLETS USP 1000 MG", "GLUMETZA METFORMIN HYDROCHLORIDE 1000 MILLIGRAM TABLETS", "JANUMET METFORMIN HYDROCHLORIDE SITAGLIPTIN PHOSPHATE MONOHYDRATE", "METFORMINA GENERIS", "METFORMIN SUSPENSION", "METFORMINA GENERIS 500 MG COMPRIMIDOS REVESTIDOS", "METFORMINE HCL A TABLET 500MG", "METFORMIN GLUCOPHAGE XR", "METFORMIN METRFORMIN", "METFORMIN AUROBINDO FILM COATED TABLETS 1000MG", "METFORMIN FILM COATED TABLETS 1000MG", "METFORMIN AUROBINDO FILMCOATED TABLETS 500MG", "GLYBURIDE METFORMIN HCL", "GLYBURIDE METFORMIN SIL NORBORAL UNKNOWN", "METFORMIN MEPHA 500MG", "METFORMIN HYDROCHLORIDE EXTENDED RELEASE TABLETS 500 MG", "METFORMIN ER METFORMIN HYDORCHLORIDE TABLET", "METFORMIN METFORMIN HYDROCHLORIDE", "KOMBIGLYZE XR METFORMIN HYDROCHLORIDE SAXAGLIPTIN", "GLIBURIDE METFORMIN", "SITAGLIPTIN W METFORMIN", "METFORMIN MEPHA 500 LACTAB", "METFORMIN HCI ER OSM", "METFORMIN HCL NON AZ PRODUCT", "METFORMIN BLUEFISH METFORMINHYDROCHLORID", "METFORMINA MYLAN ITALIA 850 MG CONPRESSE RIVESTIVE CON FILM", "GLIBENCLAMIDE METFORMIN 5MG 500MG", "METFORMIN QUILOSTIN 1000", "METFORMINA MYLAN 1000 MG COMPRESSE RIVESTITE CON FILM", "METFORMINE MYLAN PHARMA 1000 MG COMPRIME PELLICULE SECABLE", "METFORMIN HCL METFORMIN HCL NOT SPECIFIED", "PRO METFORMIN INN METFORMIN", "METFORMIN GLIFAGE", "GLIMEPIRIDE METFORMIN MERITOR", "GLICLAZIDE W METFORMIN", "SAXAGLIPTIN METFORMIN HYDROCHLORIDE", "METFORMIN HYDROCHLORIDE 1000 MG TABLETS METFORMIN HYDROCHLORIDE METFORMIN HYDROCHLORIDE", "METFORMIN METORMIN", "EUCREAS METFORMIN HYDROCHLORIDE VIDAGLIPTIN", "GLUMETAZA METFORMIN HYDROCHLORIDE", "METFORMIN METFORMIN 500 MILLIGRAM UNKNOWN", "METFORMIN AND PIOGLITAZONE", "METFORMIN GENERIC DRUG", "METFORMIN HYDROCHLORIDE METFORMIN ER", "METFORMIN HYDROCHLORIDE 500 MG TABLETS METFORMIN HYDROCHLORIDE METFORMIN HYDROCHLORIDE", "METFORMIN METFORMIN UNKNOWN", "JANUMET XR METFORMIN HYDROCHLORIDE SITAGLIPTIN PHOSPHATE MONOHYDRATE", "METFORMIN METFORMIN TABLETS", "APO METFORMIN ER", "METORMIN METFORMIN HYDROCHLORIDE", "GLUMETZA METFORMIN HCL", "GLIP METFORMIN", "GLYBURIDE MICRO METFORMIN", "LINAGLIPTIN METFORMIN", "JANMUET METFORMIN HYDROCHLORIDE", "METFORMINE MYLAN 500 MG COMPRIM DISPERSIBLE", "METFORMIN HYDROCHLORIDE METFORMIN TABLET", "METFORMINA GLIBENCLAMIDA", "METFORMIN PUREN", "GLICLAZIDE METFORMIN", "METFORMIN HEUMANN", "METFORMIN DURA 1000 MG FILMTABLETTEN", "METFORMINA VILDAGLIPTINA", "METFORMIN AUROBINDO FILMCOATED TABLET 500MG", "GLYBURIDE METFORMIN GLUCOVANCE", "METFIN 850 MG METFORMIN HYDROCHLORIDE", "NELBIS METFORMIN HYDROCHLORIDE TABLET", "GLIBENCLAMIDE METFORMIN HYDROCHLORIDE", "GLUCOVANCE METFORMIN", "METFORMIN BLUEFISH 00082702", "METFORMIN HCL XR NON AZ PRODUCT", "METFORMIN HYDROCHLORIDE TABLET", "METFORMIN NON AZ", "METFORMINE 500 MG", "METFORMIN HCL GLUCOPHAGE", "METFORMIN RATIOPHARM", "METFORMIN SAXAGLIPTIN", "METFORMIN BLUEFISH", "METFORMINA TEVA ITALIA TEVA ITALIA S R L", "METFORMIN AND GLIMEPRIDE", "METFORMIN HCL 500 MG", "GLIPIZIDE METFORMIN GLIPIZIDE METFORMIN", "GLIPIZIDE METFORMIN GLIPIZIDE METFORMIN UNKNOWN", "METFORMIN GENERIC GLUCO PHAGE ER 500 MG TAB AMNEA", "METFORMIN MYLAN 1000 MG FILM COATED TABLETS", "ACTO METFORMIN", "METFORMIN HYROCHLORIDE", "METFORMINA PENSA PHARMA", "NOVOFORMIN METFORMIN HYDROCHLORIDE", "PIOGLITAZONE HCL METFORMIN HL", "GLIFAGE METFORMIN HYDROCHLORIDE", "PIOGLITAZONE AND METFORMIN", "METFORMINA DOC", "METFORMIN HCL 850MG TABLETS", "GLUCOPHAGE METFORMIN HYDROHCLORIDE", "METFORMINE MYLAN 850 MG COMPRIM DISPERSIBLE", "GLUCOPHAGE SLOW RELEASE METFORMIN HYDROCHLORIDE", "ACTOS PLUS MET PIOGLITAZONE HYDROCHLORIDE METFORMIN HYDROCHLORIDE", "METFORMIN 750 MG", "GLUCOPHAGE XR METFORMIN", "METFORMINA DOC GENERICI", "GLYBURIDE METFORMIN METFORMIN HYDROCHLORIDE GLIB ENCLAMIDE", "METFORMIN ASTEC", "FORMATE METFORMIN HYDROCHLORIDE", "GLYBURIDE METFORMIN METFORMIN HYDROCHLORIDE GLIBENCLAMIDE", "METFONNIN METFORMIN HYDROCHLORIDE", "GLUCOPHAGE METFORMINE HYDROCHLORIDE", "GLYBURIDE METFORMIN HC", "JANUMET METFORMIN HYDROCHLORIDE SITAGLIPTIN PHOSPHATE MONOHYDRATE TABLET", "METFORMIN HCL TAB 500MG", "GLICLAZIDE METFORMIN HYDROCHLORIDE GLICLAZIDE METFORMIN HYDROCHLORIDE", "METFORMIN ARROW LAB FILM COATED TABLETS 1000MG", "CLORIDRATO DE METFORMINA", "METFORMIN HCL METFORMIN", "GLYBEN METFORMIN", "METFORMINE METFORMINE HYDROCHLORIDE", "A COMBINATION OF GLYBURIDE AND METFORMIN", "METFORMIN HYDROCHLORIDE 500 MG FILM COATED TABLETS", "AMARYL M GLIMEPRIDE 1 MG METFORMIN 250 MG", "METFORMIN HYDROCHLORIDE TABLETS 850MG", "METFORMINVESICARE", "LINAGLIPTIN WITH METFORMIN", "C PAP MACHINE METFORMIN", "METFORMINE METFROMIN HYDROCHLORIDE", "METFORMIN HYDROCHLORIDE AND GLIBENCLAMIDE", "VELMETIA SITAGLIPTIN PHOSPHATE METFORMIN HYDROCHLORIDE", "ICANDRA METFORMIN VILDAGLIPTIN", "SAVI METFORMIN", "METFORATIO METFORMIN", "METFORMIN GENERICS 850 MG TABLETS", "MEDFORMIN METFORMIN HYDROCHLORIDE", "VILDAGLIPTINE AND METFORMIN", "GENERIC METFORMIN", "METFORMIN BLUEFISH 00082701", "GLUCOPHAGE UNKNOWN METFORMIN HYDROCHLORIDE", "SUKKARTO METFORMIN HYDROCHLORIDE", "METFORMIN INN", "METFORMIN UNKNOWN METFORMIN HYDROCHLORIDE METFORMIN HYDROCHLORIDE", "METFORMINA TEVA COMPRESSE RIVESTITE 500 MG TEVA ITALIA S R L", "METFORMIN GLUCOPHAGE", "SAXAGLIPTIN METFORMIN XR", "METFORMIN SPIRIG HC", "METFORMIN HELXR", "METFORMINE GLIPIZIDE", "METFORMIN AUROBINDO", "PIOGLITAZONE HCL AND METFORMIN HCL", "AURO METFORMIN 850", "METFORMIN 500MG AMNEAL PHARMACEUTICALS", "METFORMINA AUROBINDO COMPRIMIDOS RECUBIERTOS CON PELICULA 850MG", "GLIPIZIDE METFORMINE", "METOPROLOL METFORMIN", "METFORMIN HCL ER TABLETS 500 MG", "GLIP METFORMIN 5 500MG MYLAN", "EUCREAS METFORMIN HYDROCHLORIDE VILDAGLIPTIN METFORMIN HYDROCHLORIDE VILDAGLIPTIN", "CANAGLIFLOZIN HEMIHYDRATE W METFORMIN HYDROCH", "JANUMET METFORMIN HYDROCHLORIDE SITAGLIPTIN PHOSPHATE", "METFORMIN FORTAMET", "METFORMIN GLIBENCLAMIDE", "DAPAGLIFLOZIN AND METFORMIN 5MG AND 1000MG ASTRAZENECA", "METFORMINE ACTAVIS", "METFORMINA TEVA 56 COMPRESSE RIVESTITE TEVA ITALIA S R L", "BLINDED METFORMIN HYDROCHLORIDE", "GLIPIZIDE AND METFORMIN HYDROCHLORI 01643101", "LINAGLIPTIN METFORMIN", "IC METFORMIN HCL ER 500 MG TEVA USA", "METFORMIN ER TAB GENERIC GLUCOPHAGE XR 500 MG ASCEND LABORATORIES LLC", "METFORMIN 500MG TAB HER HERITAGE", "METFONORM 1000MG COMPRESSE RIVESTITE CON FILM METFORMIN", "METFORMINA MYLAN 500 MG COMPRESSE RIVESTITE CON FILM", "GLIMEPIRIDE METFORMIN", "METFORMIN HCL SUGAR", "METFORMIN HCL GLUCOPHAGE", "ROSIGLITAZONE MALEATE METFORMIN HYDROCHLORIDE TABLET", "METFORMIN EXTENDED RELEASE 24 HR", "METFORMIN MODIFIED RELEASE TABLET", "GLY MET GLYBURIDE METFORMIN", "JUNEVIA METFORMIN", "DAPAGLIFLOZIN METFORMIN XR", "DAPAGLIFLOZIN WITH METFORMIN XR", "METFORMIN ER 750MG AMNEAL PHARMACEUTICALS", "METFORMIN GENERIS", "DAPAGLIFLOZIN METFORMIN", "METFORMIN XR SITAGLIPTIN", "METFORMINE 850 MG", "DAPAGLIFLOZIN W METFORMIN", "VILDAGLIPTIN METFORMIN CHLORIDRATE 50 1000MG", "METFORMIN GLUMETZA", "METFORMIN ER 500 MG AMNEAL", "METFORMIN HYDROCHLORIDE EXTENDED RELEASETABLETS 1000 MG", "METFORMIN HYDROCHLORIDE EXTENDED RELEASE TABLETS USP 500 MG", "METFORMIN 500MG TEVA", "VILDAGLIPTIN WITH METFORMIN", "GLYCOL GLIBENCLAMIDE METFORMIN HYDROCHLORIDE", "METFORMINE HCL ACTAVIS", "METFORMIN ARROW LAB FILM COATED TABLETS 500MG", "METFORMIN FC", "METFORMINA MYLAN GENERICS 1000 MG COMPRESSE RIVESTITE CON FILM", "METFORMIN GLICLAZIDE", "METFORMIN HCL", "METFORMIN ALKEM", "METFORMIN HCL TABLETS USP 500MG", "METFORMINA 00082701 METFORMIN", "METFORMIN HYDROCHLORIDE TABLET FILM COATED", "METFORMIN STADA", "GLYBURIZIDE METFORMIN", "GLYBURIZIDE METFORMINE", "METFORMINA TEVA COMPRESSE RIVESTITE 850 MG", "METFORMINA TEVA 850 MG CPR RIVESTITE TEVA ITALIA S R L", "METFORMIN 500 MILLIGRAMS ENDURE PHARMACEUTICAL LED INDIA", "METFORMINA CLORHIDRATO GLIBOMET", "JARDIANCE METFORMIN", "GLYBURIDE METFORMIN HYDROCHLORIDE", "ACTAVIS UK METFORMIN", "METFORMINA METFORMIN", "METFORMIN HCL 500MG 2X A DAY", "METFORMIN GLICLZIDE", "METFORMIN HYDROCHLORIDE SAXAGLIPTIN HYDROCHLORIDE", "GLUCOMET METFORMIN HYDROCHLORIDE", "METFORMINHCL", "METFORMIN ER 500MG SUN PHARMA", "METFORMIN TABLET 1000MG AUROBINDO", "METFORMIN CHLOROPHENOXYACETATE", "GLIPIZIDE WITH METFORMIN", "BLINDED GLYBURIDE METFORMIN HYDROCHLORIDE", "GENERICS UK METFORMIN", "METFORMIN NON ABBVIE", "GLYBURIDE METFORMIN 5 MG 500 MG", "AURO METFORMIN", "METFORMIN 500 MG GENERIC", "METFORMINE HCL", "METFORMIN MEPHA", "METFORMIN DAPAGLIFLOZIN", "METFORMIN LICH 1000 MG", "METFORMIN 1000 MG TAB HERITAGE PHARMACEUTICALS", "GLYBURIDE METFORMIN 2 5 500MG TAB", "METFORMIN LIPATOZIDE", "VILDAGLIPTIN METFORMIN", "ALOGLIPTIN METFORMIN 12 5 10", "METFORMIN 500MG TAB ZYD WLAMART", "LINAGLIPTIN METFORMINE", "METFORMIN ER 500 MG TABLET CODE ON TABLETS IP178 WHITE OBLONG", "METFORMIN NONTIME RELEASE", "METFORMINE ARROW 500 MG COMPRIM PELLICUL", "METFORMIN HYDROCHLORIDE W PIOGLITAZ 02910801", "DAPAGLIFLOZIN METFORMIN", "METFORMIN HYDROCHLORIDE INGENUS", "METFORMIN DR", "GLYUBRITE METFORMIN", "METFORMIN 1000 MG AMNEAL", "METFORMIN ATID", "GLIMIPERIDE METFORMIN", "METFORMIN HYDROCHLORIDE TABLETS USP", "GLUCOPHAGE METFORMIN", "METFORMINA TEVA COMPRESSE RIVESTITE TEVA ITALIA S R L", "IC METFORMIN HCL 500MG TABLET", "GIB METFORMIN", "GLIPIZIDE AND METFORMIN HYDROCHLORI 01182201", "METFORMINA CINFA", "METFORMIN XR 750", "ALOGLIPTIN METFORMIN", "METFORMIN HYDROCHLORIDE MODIFIED RELEASE", "METFORMIN HCL 500 MG ZYDUS PHARMACEUTICAL", "CANAGLIFLOZIN W METFORMIN HYDROCHLORIDE", "ACARBOSE W METFORMIN", "METFORMIN 850 MG", "METFORMIN ARMOUR", "METFORMINA APOTEX", "METFORMIN HYDROCHLORIDE MT", "METFORMIN CARARO", "METFORMIN ER 500 MG TAB GENERIC FOR GLUCOPHAGE XR 500 MG TAB", "METFORMIN HYDROCHLORIDE VOGLIBOSE", "METFORMIN HC 500 MG CORACO PHARM", "ACTOS WITH METFORMIN", "SYNJARDY EMPAGLIFLOZIN METFORMIN", "GLYBURIDE METFORMIN AELLC", "METFORMIN LICH", "METFORMIN ER 500MG", "METFORMINA TEVA 300 CPR RIVESTITE", "METFORMIN 1 000MG MYLAN", "METFORMIN SLOW RELEASE", "METFORMINA TEVA ITALIA 1000 MG COMPRES SE RIVESTITE CON FILM", "METFORMIN HCL UNK", "METFORMIN SITAGLIPTAN", "METFORMIN REPORTED AS METFORMIN HCL", "VILDAGLIPTIN PLUS METFORMIN", "METFORMIN 50 1000", "PIOGLITA METFORMIN", "APO METFORMIN TAB 500MG", "METFORMIN HEXAL PHARMA", "METFORMIN PROLONGED RELEASE TABLET", "METFORMIN GLIPIZIDE", "METFORMIN HCL TABS 1000MG 1000 MG", "JANUVIA METFORMIN", "SITAGLIPTIN METFORMIN", "GLIFOR 1000MG METFORMIN HCL BILIM ILAC SAN VE TIC A S", "METFORMIN MULTIVITAMIN", "METFORMIN 1000 MG VILDAGLIPTIN 50 MG", "SITAGLIPTIN AND METFORMIN", "METFORMIN GLUCOFAGE", "METFORMIN HCL W PIOGLITAZONE", "METFORMIN HCL 550MG", "GLISULIN METFORMIN HYDROCHLORIDE", "METFORMIN XARALTO SIMVASTATIN LIPITOR", "METFORMINA TEVA ITALIA", "METFORMINE HCI AUROBINDO 500 MG FILM COATED TABLETS", "GLYBURIDE METFORMIN TAB", "METFORMIN AUROBINDO", "METFORMIN HYDRCHLORIDE", "METFORMIN N", "APO METFORMIN 850 MG TABLETS", "METFORMIN 500MG TAB ZYDUS", "METFORMIN HCL ER 500MG TB24", "CANAGLIFLOZIN METFORMIN", "METFORMINA TEVA TEVA ITALIA S R L", "GLUCOPHAGE GENERIC METFORMIN ER BRAND 500 MG", "METFORMIN HCL ER 500 MG TABS AOTEX CORP", "GLUCOPHAGE METFORMIN HCL", "METFORMINA PNS", "METFORMIN HCL 500MG PHARMA USA INS", "CANAGLIFLOZIN METFORMIN", "METFORMIN IR", "GLIMEPIRIDE VOGLIBOSE METFORMIN", "METFORMIN HCL 500 MG", "ACTAVIS METFORMIN", "ALOGLIPTIN W METFORMIN", "SAXAGLIPTIM METFORMIN", "METFORMINE HCL APOTEX OMHULDE TABLETTEN", "METFORMIN FORTE", "METFORMIN HYDROCHLORIDE EXTENDED", "TENELIGLIPTIN METFORMIN", "METFORMIN EXTENDED RELASE", "METFORMIN GLIPISIDE", "METFORMINE HCL SANDOZ TABLET FILMOMHULD 500MG", "VICTOZA JANUVIA METFORMIN EFFEXOR ADVAIR SIMBICORT", "REPAGLINIDE METFORMIN", "METFORMIN ER 500 MG TABLET TEVA USA", "METFORMINA KERN PHARMA 850 MG", "METFORMIN ACCEDO", "METFORMIN HCZL", "METFORMIN HCL 500 MGTABS", "PIOGLITAZONE AND METFORMIN HYDROCHLORIDE TABLETS", "ADIMET METFORMIN", "METFORMIN ER 1000 MG", "METFORMIN HCL ER TABLETS", "METFORMIN RATIOPHARM COMPRIMIDOS RECUVIERTOS CON PELICULA 500MG", "METFORMIN ASCEND", "METFORMIN HYDROCHLORIDE VILDAGLIPTIN", "GLYBURIDE AND METFORMIN HYDROCHLORIDE TABLETS 2 5 500 MG", "VIDAGLIPTINA METFORMINA", "GLIPIZIDE METFORMIN HYDROCHLORIDE", "METFORMIN HCI EXTENDED RELEASE SUN PHARMACEUTICALS", "METFORMIN SITAGLIPTINE", "METFORMIN ACTAVIS 500 MG FILM COATED TABLET", "VYTORIN METFORMIN ER", "METFORMIN HYDORCHLORIDE ER", "METFORMIN HYDROCHLORIDE PIOGLITAZONE HYDROCHLORIDE", "GLIPIZIDE METFORMIN ER", "METFORMIN 500MG TABLET ZYDUS", "METFORMIN HYDROCHLORIDE GLIBENCLAMIDE", "ACT METFORMIN", "SAXAGLIPIN METFORMIN", "METFORMINMETFORMIN", "METFORMIN BLUEFISH 00082702", "METFORMINA MYLAN ITALIA", "METFORMIN INVENTIA", "METFORMIN HYDROCHLORIDE 1000 MG FILM COATED TABLETS", "METFORMIN TABLET 500 MG", "METFORMIN HCL ER MOD", "APO METFORMIN 1000 MG", "METFORMIN MEPHA", "METFORMIN HYDROCHLORIDE EXTENDED RELEASE TABLETS 500 MG 1000 MG FOR", "METFORMIN HYDROCHLORIDE TABLETS USP 850 MG", "COMBINATION OF GLICLAZIDE 40 MG WITH METFORMIN 250 MG", "MF METFORMIN", "METFORMINE MYLAN 500 MG", "METFORMIN ARROW 850MG", "MYLAN METFORMIN 850 MG", "METFORMIN HYDROCHLORIDE ER", "METFORMIN SCL ER", "METFORMIN HCL ER 500 MG TABLET", "METFORMIN HCL 500MG TAB", "METFORMIN GLIFAGE MR", "METFORMIN HLC", "SITAGLIPTAN METFORMIN", "METFORMIN RATIOPHARM 1000MG", "METFORMIN TOLBUTAMIDE", "METFORMIN TE 250", "METFORMIN MAH UNKNOWN", "METFORMIN N GLUCOPHAGE XR", "METFORMIN BIOMO", "METFORMINA TEVA 500 MG CPR RIVESTITE TEVA ITALIA S R L", "METFORMIN 1000 XR ESTELLE CYPROTERONE COMPOUND", "METFORMIN HDL", "METFORMIN 25 MG", "METFORMIN HYDROCHLORIDE SITAGL IPTIN PHOSPHATE MONOHYDRATE", "APO METFORMIN 500 MG", "METFORMINA SITAGLIPTINA", "METFORMIN HCL 500 MG TABLET", "GLIPIZIDE W METFORMIN", "PIOGLITAZONE 15MG METFORMIN 850MG", "METFORMIN HYDROCHLORIDE EXTENDED RELEASE TABLET 500MG AND 1000MG", "METFORMINA TEVA 30 CPR RIVESTITE TEVA ITALIA S R L", "SITAGLIPTIN METFORMIN", "METFORMIN HYDROCHLORIDE EXTENDED RELEASE TABLET USP 1000 MG", "METFORMIN E R", "EXTENDED RELEASE METFORMIN", "METFORMIN ER 500MG TAB AMN", "GALVUS MET VILDAGLIPTIN METFORMIN HYDROCHLORIDE", "METFORMIN ACTAVIS 500 MG", "METFORMINE HCL TEVA", "JAMP METFORMIN BLACKBERRY", "METFORMIN HYDROCHLORIDE W SITAGLIPT 06535301", "SAXAGLIPTINE METFORMINA", "SAXAGLIPTINE METFORMINE", "METFORMIN HYDROCHLORIDE W SITAGLIPT 06902601", "EMPAGLIFLOZIN W METFORMIN HYDROCHLORIDE", "METFORMIN INSULIN", "METFORMINA CLORHIDRATO GLIBENCLAMIDA", "METFORMIN NON TEVA", "LINAGLIPTIN W METFORMIN HYDROCHLORIDE", "LINAGLIPTIN W METFORMIN HYDROCHLORIDE", "A PRODUCT SIMILAR TO METFORMIN", "METFORMIN HCL AUROBINDO PHARMA 5", "METFORMIN HYDROCHLORIDE GLICLAZIDE", "GLIMEPIRIDE AND EXTENDED RELEASE METFORMIN HYDROCHLORIDE TABLET", "GLIMEPIRIDE 1 MG METFORMIN 500 MG VOGLIBOSE 0 2 MG TABLETS", "METFORMIN HCL 500 MG TABS ER", "APO METFORMIN XR 500", "GLYCOMIN METFORMIN HYDROCHLORIDE", "METFORMIN ARROW FILM COATED TABLET 500 MG", "METFORMIN AUROBINDO FILMTABLETTEN 500MG", "METFORMIN HYDROCHLORIDE EXTENDED RELEASE TABLET", "AUSTELL METFORMIN", "METFORMINE 500", "METFORMIN 40", "METFORMIN XR 24H", "METFORMIN HCL 1 000MG", "METFORMIN HYDROCHLORIDE W SITAGLIPT", "APO METFORMINA", "VIDAGLIPITINA METFORMINA", "JANUMET XR METFORMIN SITAGLIPTIN", "METFORMIN HCL 1 000MG TA", "METFORMIN HCL ER TABLET 500 MG", "METFORMIN ER AMNEAL", "METFORMIN HYDROCHLORIDE EXTENDED RELEASE TABLETS USP", "METFORMINA GLIBENCLAMIDA 01182201", "ALOGLIPTIN METFORMIN 12 5 1000 MG", "SANDOZ METFORMIN", "METFORMIN MEPHA LACTAB", "METFORMINE 00082701", "JANUMET METFORMIN HYDROCHLORIDE SITAGLIPTIN PHOSPHATE MONOHYDRATE", "METFORMIN SITAGLIPTIN EFFICIB", "METFORMIN 1000MG TABLETS", "CANAGLIFLOZIN W METFORMIN", "SITAGLIBIN METFORMIN", "METFORMIN AXCOUNT", "METFORMIN 750 ER", "METFORMIN DOC GENERICI FILM COATED TABLETS 850MG", "ACTOS 15MG WITH METFORMIN 500MG", "METFORMIN ARROW FILM COATED TABLET 850MG", "GLIPIZIDE METFORMIN 5MG 500MG TABS", "METFORMIN EXACT PRODUCT NAME NOT REPORTED", "METFORMIN SAXAGLIPTINE", "METFORMIN NIHFI", "METFORMIN STAGLIPTIN", "METFORMIN GENERIC FOR GLUCOPHAGE", "METFORMIN HCL ER 500MG IC METFORMIN HCL", "METFORMIN HCI GLUCOPHAGE", "WINSULIN METFORMIN HYDROCHLORIDE", "METFORMIN HYDROCHLORIDE EXTENDED RELEASE TABLET USP 1000 MG", "METFORMIN HYDROCHLORIDE EXTENDED RELEASE TABLET USP 500 MG", "METFORMIN HYDROCHLORIDE EXTENDED RELEASE TABLET USP 500 M G", "GLUCONORM METFORMIN HYDROCHLORIDE", "METFORMIN AND LINAGLIPTIN", "SANDOZ METFORMIN 500 MG", "METFORMIN HYDROCHLORIDE GLIBENCLAMIDE", "METFORMIN ER 24 HR TABS V", "VILDAGLIPTIN METFORMINE HYDROCLORIDE", "METFORMINE 00082702", "METFORMIN SUCCINATE", "DEXIDE METFORMIN HYDROCHLORIDE", "VILDAGLIPTIN METFORMIN HYDROCHLORIDE", "METFORMINE 00082701", "METFORMIN HYDROCHLORIDE 1000MG TABLET", "METFORMIN APS", "METFORMINE NOS", "METFORMIN HYDROCHLORIDE 500MG MT PFIZER", "METFORMINE TABLET 1000 MG MILLIGRAM", "METFORMIN HYDROCHLORIDE EMPAGLIFLOZIN", "CANAGLIFLOZIN METFORMIN HYDROCHLORIDE", "SAXAGLIPTIN HYDROCHLORIDE METFORMIN HYDROCHLORIDE", "METFORMIN 850 1A PHARMA", "ALOGLIPTIN BENZOATE METFORMIN HYDROCHLORIDE", "METFORMIN HYDROCHLORIDE W SAXAGLIPTIN", "METFORMIN HYDROCHLORIDE EXTENDED RELEASE TABLET 750MG", "METFORMINA HCL", "METFORMIN HYDROCHLORIDE 500MG TABLET", "METFORMINE HCL TABL 500MG", "PIOGLITAZONE AND METFORMIN HYDROCHLORIDE", "JANUMET RISTFOR METFORMIN HYDROCHLORIDE SITAGLIPTIN PHOSPHATE MONOHY", "METFORMINA ABEX 850 COMPRIMIDOS RECUBIERTOS CON PELICULA EFG 50 C", "METFORMINA VILDAGLIPTINA", "METFORMINA 1359A", "METFORMIN DAPAGLIFLOZIN", "DAPAGLIFLOZINA METFORMINA", "METFORMINA ABEX 850 COMPRIMIDOS RECUBIERTOS CON PELICULA EFG 50 COMP", "METFORMINA ABEX 850 COMPRIMIDOS RECUBIERTOS CON PELICULA EFG 50 COMP", "METFORMIN HYDROCHLORIDE ER 1000 MG", "SITAGLIPTINA METFORMINA", "MYLAN METFORMIN 500 MG", "VILDAGLIPTIN METFORMIN HYDROCHLORIDE COMBINED DRUG", "METFORMIN BYSTOLIC", "METFORMIN HYDROCHLORIDE 850MG", "METFORMIN FILM COATED TABLET", "METFORMINE CHLORHYDRATE DE", "GALVUS MET VILDAGLIPTIN METFORMIN0", "METFORMINE ARROW 1000 MG", "METFORMINA ABEX 850 COMPRIMIDOS", "METFORMIN ER TAB 500MG GP AMNEAL PHARMACEUTICALS", "EMPAGLIFLOZIN METFORMIN", "METFORMINA 850", "METFORMINA BIOCHEMIE", "PIOGLITAZONE AND METFORMIN HCL", "METFORMIN HCL DR 500 MG TABS", "METFORMINA TEVA ITALIA 1000 MG COMPRESSE RIVESTITE CON FILM", "METFORMIN HYDROCHLORIDE 500MG", "METFORMIN 100MG 1 PO BID", "METFORMIN HYDROCHLORIDE SITAGLIPTIN PHOSPHATE", "METFORMIN W SITAGLIPTIN METFORMIN W SITAGLIPTIN", "GLIFOR METFORMIN HYDROCHLORIDE", "METFORMIN HCIER", "METFORMIN TABLETS 750MG XR", "METFORMIN ACTAVIS 1000 MG FILMTABLETTEN", "METFORMIN TEVA 500 MG FILM COATED TABLETS", "METFORMIN 1359A", "METFORMIN FILM COATED TABLET", "METFORMIN ER 500 MG", "METFORMINE MYLAN 850 MG COMPRIM PELLICUL S CABLE", "METFORMIN CLORIDRATO", "METFORMIN ACTAVIS 500 MG FILMDRAGERAD TABLETT", "METFORMIN HYDROCHLORIDE SITAGLIPTIN PHOSPHATE MONOHYDRATE", "METFORMIN XR 24 HOUR TAB", "METFORMIN ER 1000 MG PO BID", "METFORMIN TAB 1000MG", "METFORMIN 1000MG BID", "GALVUS METFORMINA", "METFORMIN HYDOCHLORIDE EMPAGLIFLOZIN", "METFORMIN METFORMIN HCL", "GLYBURIDEMETFORMIN", "METFORMINE ARROW 850 MG COATED TABLET", "METFORMIN HYDROCHLORIDE SUSTAINED RELEASE TABLET", "METFORMIN 500MG BID", "METFORMINA HIDROCLORURO", "METFORMIN HYDROCHLORIDE 1359CH", "METFORMIN HYDROCHLORIDE FILM COATED TABLET", "METFORMIN 850 MG 50 TABLETS", "METFORMIN HYDROCHLORIDE 850MG TABLET", "METFORMIN 1 0000 MG TWICE DAILY", "METFORMIN 500MG TAB", "SAXAGLIPTIN METFORMIN", "COMPETACT METFORMIN PIOGLITAZONE", "METFORMIN TYLENOL", "METFORMIN HCL FILM COATED TABLETS 500MG", "METFORMINE TABLET 500 MG", "METFORMINE WINTHROP", "METFORMIN GLIMEPIRID", "METFORMINE 500MG TABL", "METFORMIN 500 MG TWICE A DAY", "SITAGLIPTIN PHOSPHATE METFORMIN HCL", "METFORMINE TEVA 500 MG COMPRIM PELLICUL", "METFORMIN VILDAGLIPTIN", "METFORMINA TEVA 500 MG COMPRESSE RIVESTITE CON FILM", "ALOGLIPTIN BENZOATE METFORMIN HYDROCHLORIDE", "METFORMIN HYDROCHLORIDE LINAGLIPTIN", "PIOGLITAZONE AND METFORMIN HYDROCHOLRIDE", "METFORMINE ACTAVIS 850 MG COMPRIM PELLICUL", "METFORMIN XR 500MG", "EMBONATE DE METFORMINE", "METFORMIN AND GLYPIZIDE", "METFORMINA HIDROCLORURO 1359CH", "METFORMIN DOC GENERICI FILM COATED TABLETS 1000MG", "GLUCOVANCE GLIBENCLAMIDE METFORMIN HYDROCHLO", "METFORMIN 750 ML", "METFORMIN COMP", "METFORMINA 850 MG COMPRIMIDO", "METFORMIN HCL FILM COATED TABLETS 1000MG", "METFORMINA RATIOPHARM", "METFORMIN NOS", "METFORMINE ARROW 1000 MG COMPRIM PELLICUL S CABLE", "METFORMINE ARROW 850 MG COMPRIM PELLICUL", "METFORMIN VIDAGLIPTIN", "VOKANAMET CANAGLIFLOZIN HEMIHYDRATE METFORMIN HYDROCHLORIDE", "EMPAGLIFLOZIN METFORMIN HYDROCHLORIDE", "METFORMINA RATIOPHARM ITALIA", "METFORMIN HYDROCHLORIDE UNKNOWN FORMULATION", "METFORMIN 1000MG PO BIDWM", "METFORMIN TABLETS BP 850 MG", "VILDAGLIPTINA METFORMINA", "CHLORHYDRATE DE METFORMINE", "METFORMIN AUROBINDO 500 MG FILM COATED TABLETS", "METFORMINA MYLAN ITALIA 1000 MG COMPRESSE RIVESTITE CON FILM", "METFORMINE 00082702", "METFORMIN ER TAB GP", "METFORMINA METFORMIN HYDROCHLORIDE", "ALOGLIPTIN AND METFORMIN HCL", "METFORMIN 750MG ER", "METFORMIN ACTAVIS 500 MG FILMUH A AR T FLUR", "METFORMIN MEPHA LACTAB 1000MG", "METFORMINE HCL ACTAVIS TABLET 500MG", "METFORMINE ZENTIVA", "METFORMIN GLIBENCLAMID", "VILDAGLIPTINA METFORMINA", "DM2 METFORMIN HYDROCHLORIDE", "METFORMIN 500MG 1 PO BID", "METFORMINE RATIOPHARM", "METFORMIN HYDROCHLORIDE SAXAGLIPTIN HYDROCHLORIDE", "METFORMIN MEPHA LACTAB 850 MG TABLETS", "METFORMIN RICHTER", "METFORMIN 500 ER", "METFORMIN 500 BID", "ALOGLIPTIN BENZOATE W METFORMIN HYDROCHLORIDE", "METFORMIN HYDROCHLORIDE 500 MG TABLETS USP", "METFORMIN 1000 MG TWICE DAILY", "METFORMIN MODERATE RELEASE 500 MG", "GLUFORMIN METFORMIN HYDROCHLORIDE", "METFORMIN HYDROCHLOROIE", "VOKANAMET CANAGLIFLOZIN METFORMIN HYDROCHLORIDE", "METFORMINA ALOGLIPTINA", "METFORMIN HYDROCHLORIDE TENELIGLIPTIN HYDROBROMIDE", "METFORMIN AND INSULIN SLIDING SCALE", "METFORMIN ALOGLIPTIN", "CALCIUM METFORMIN", "LITHIUM METFORMIN", "METFORMIN500MG", "METFORMIN HYDROCHLORIDE TABLET USP 1000 MG", "METFORMIN HYDROCHLORIDE TABLET 500MG 850 MG AND 1000MG", "METFORMINE TEVA 1000 MG COMPRIM PELLICUL", "METFORMINE ARROW 500 MG COATED TABLET", "METFORMIN HCL E R", "METFORMINE SITAGLIPTIN", "TEFOR METFORMIN", "METFORMINA 875", "METFORMIN XR 500 MG", "METFORMIN CYMBALTA", "METFORMIN HCL ER 500 MG TA ER 24H", "METFORMIN 1000 MG BID AC", "METFORMIN 1000 TABLET", "SITAGLIPTINE METFORMINE", "SITAGLIPTINA Y METFORMINA", "METFORMIN ER 1000MG TWICE DAILY", "METFORMINE 500MG", "CLOPIDOGREL METFORMIN", "GLYBURIDE METFORMIN 2 5 MG 400 MG", "METFORMIN 500 MG TABLET", "METFORMIN TAB 500MG", "ISRADIPINE METFORMIN", "METFORMIN MOLECULE FROM UK MARKET", "DYNARB METFORMIN HYDROCHLORIDE", "METFORMIN HCI 500 MG", "METFORMIN 1000 MG SITAGLIPTIN 50 MG", "METFORMIN GENERIS 1000 MG FILM COATED TABLETS", "METFORMIN ER 1000MG BID", "METFORMIN HYDROCHLORIDE SITAGLIPTIN PHOSPHATE", "METFORMINA AUROBINDO", "METFORMIN HYDROCHLORIDA TABLETS USP 1000 MG", "METFORMIN HCL 1000 MG TABLET", "METFORMINA 850 MG 50 COMPRIMIDOS", "METFORMIN AUROVITAS FILM COATED TABLET 1000 MG", "PIOGLITAZONE METFORMIN ACTOPLUS MET 15 500 MG TABLET", "METFORMIN 1000 MG ER PO BID", "METFORMIN PFIZER FILMDRAGERADE TABLETTER 500MG", "EMPAGLIFLOZIN METFORMIN HYDROCHLORIDE", "METFORMIN AUROBINDO 1000 MG FILM COATED TABLET", "METFORMINA PLUS VILDAGLIPTINA", "DAPAGLIFLOZIN PROPANEDIOL METFORMIN HYDROCHLORIDE", "METFORMIN W VILDAGLIPTIN", "METFORMIN HCTZ", "METFORMIN ER 500", "GLYBURIDE AND METFORMIN TEVA", "METFORMINUM METFORMIN HYDROCHLORIDE", "ZOMARIST METFORMIN HYDROCHLORIDE VILDAGLIPTIN", "VILDAGLIPTIN METFORMIN HCL", "METFORMIN HCL 1 00 MG TABLET", "METFORMIN ER GASTRIC", "METFORMIN HCL AUROBINDO FILM COATED TABLETS 500MG", "METFORMIN PICKED UP AT LOCAL PHARMACY CVS IN NEW BERN NC", "METFORMIN AND DPP4 INHIBITOR SITAGLIPTIN", "AURO METFORMIN TABLET", "METFORMINUM METFORMIN HYDROCHLORIDE", "METFORMIN METOPROL", "METFORMIN HYDROCHLORIDE VILDAGLIPTIN", "METFORMIN XR 2000 MG", "METFORMIN ER 2000 MG", "METFORMIN XGEVA ATORVASTATIN", "METFORMIN CLOBETASOL", "METFORMIN BLUEFISH", "METFORMINA TEVA 850 MG COMPRESSE RIVESTITE CON FILM", "GLYBURIDE AND METFORMIN HYDROCHLORIDE TABLETS 1 25 250 MG", "METFORMIN HYDROCHLORIDE 850 MG TABLETS USP", "METFORMIN HCL EXTENDED RELEASE METFORMIN ER", "METFORMIN HCL TAB 500 MG", "METFORMIN HYDROCHLORIDE SUSTAINED RELEASE TABLETS", "METFORMIN AMNEAL", "METFORMINA MYLAN GENERICS 500 MG COMPRESSE RIVESTITE CON FILM", "METFORMIN 50MG", "METFORMIN HCL METFORMIN HCL", "APOTEX METFORMIN XR 500", "METFORMIN 1000MG TWICE DAILY", "METFORMIN HYDROCHLORIDE SITAGLIPTIN PHOSPHATE MONOHYDR", "METFORMIN WITH SITAGLIPTIN", "METFORMIN GLUCOPHAGE 500 MG TABLET", "MELOXICAM GABAPENTIN METFORMIN PREMARIN GEMFIBROZIL FREESTYLE", "METFORMIN 750 MG ER", "METFORMIN SULPHONYLUREA", "METFORMINE METFORMIN HYDROCHLORIDE", "METFORMIN XR GLUCOPHAGE", "ALOGLIPTIN METFORMIN", "METFORMIN HCL FILM COATED TABLETS 850 MG", "METFORMIN HCL 100MG", "EMPAGLIFLOZIN METFORMIN XR", "METFORMIN HYDROCHLORIDE EXTENDED RELEASE TABLET 500MG AND 1000MG TEVA", "METFORMINA ALMUS", "METFORMIN ER OSMOTIC", "METFORMIN ARROW 500 MG FILM COATED TABLETS", "METFORMINA 850 MG", "HYDROCHLOROT METFORMIN METOCLOPRAM", "ZOMARIST METFORMIN HYDROCHLORIDE VILDAGLIPTI", "METFORMIN 500MG AT LUNCH", "METFORMIN HYDROCHLORIDE EXTENDED RELEASE TEVA", "METFORMIN VITABALANS", "METFORMINE ZYDUS", "METFORMIN HYDROCHLORIDE ER ACTAVIS", "METFORMIN HYDROCHOLRIDE HCL 1000MG TAB", "GLICLAZIDE METFORMIN ROSIGLITAZONE MALEATE", "METFORMIN HYDROCHLORIDE 00082702", "METFORMIN 1000MG 2 X DAILY", "METFORMIN DULERA MONTELUKAST BACTROBAN", "METFORMIN 1000 MG TABLETS", "METFORMINA 1359A", "METFORMINE ARROW 500 MG COMPRIME PELLICULE", "METFORMIN POW", "PIOGLITAZONE METFORMIN 15 850 MG 04 28 2018BID", "METFORMINE TABLET 500 MG MILLIGRAM", "METFORMIN 1000 MG BY MOUTH TWICE DAILY", "LINAGLIPTIN METFORMIN HYDROCHLORIDE", "METFORMINA KERN PHARMA 850 MG COMPRIMIDOS RECUBIERTOS CON PELICULA EFG", "METFORMIN EXTENDED RELEASE TABLET TEVA", "METFORMIN HCL 1000 MG", "METFORMIN PREDNISONE", "METFORMIN 500 MG PO BID", "METFORMIN AND JANUVIA COMBINATION", "METFORMIN DAPAGLIFLOZIN", "METFORMIN AND SITAGLIPTIN PHOSPHATE", "METFORMINE CHLORHYDRATE DE VILDAGLIPTINE", "METFORMIN METOPROLOL", "METFORMIN PCOS", "DAPAGLIFLOZIN METFORMIN SAXAGLIPTIN", "METFORMINE ACTAVIS 1000 MG FILM COATED TABLET", "METFORMINE HCL 500MG", "DAPAGLIFLOZIN PROPANEDIOL MONOHYDRATE METFORMIN HYDROCHLORIDE", "METFORMIN LINAGLIPTIN EMPAGLIFLOZIN", "METFORMIN 800 MG", "METFORMIN 2000 MG XR DAILY", "METFORMIN HYDROCHLORIDE NON GSK COMPARATOR", "METFORMINE HCI 550MGMETFORMINE TABLET 500MG", "METFORMIN ER OSM", "METFORMIN HYDROCHLORIDETABLETS", "MAGNESIUM MELATONIN METFORMIN", "METFORMINE ER", "METFORMIN ER 500 MG TAB", "GLIMEPIRIDE METFORMIN", "METFORMIN HYDROCHLORIDE 1000 MG", "METFORMIN EXTENDED RELEASE TABLETS", "METFORMIN HCL ER 500MG TABLET GENERIC FOR GLUCOPHAGE XR 500MG TAB", "METFORMIN HYDROCHLORIDE MT PFIZER", "METFORMINE HCL ER", "METFORMIN HYDROCHLORIDE TABLET 500MG TEVA", "METFORMIN 1000 MG DAILY", "MIRTAZAPINE METFORMIN PRAVASTATIN", "METFORMINE TABLET 500MG ORAAL", "METFORMINE 500 MG", "METFORMIN HCL 500 MG TAB", "METFORMINA 850 MG COMPRIMIDOS RECUBIERTOS CON PELICULA EFG 50 COMPRIM", "METFORMIN HYDROCHLORIDE SITAGLIPTIN PHOSPHATEIPTIN PHOSPHATE", "METFORMIN HYDROCHLORIDE EXTENDED RELEASE TABLET TEVA", "BRISTOL LABS METFORMIN", "METFORMIN ENTERIC COATED TABLETS", "METFORMIN BRISTOL LABS", "METFORMIN HYDROCHLORIDE TOLBUTAMIDE", "METFORMIN TAB 1000 MG", "JUNLIDA METFORMIN HYDROCHLORIDE", "METFORMINA EUROGENERICI 500 MG COMPRESSE RIVESTITE CON FILM", "METFORMIN MELEATE", "TEVA S METFORMIN HYDROCHLORIDE EXTENDED RELEASE", "METFORMIN HCL ER 24HR 500MG TAB GENERIC FOR GLUCOPHAGE", "METFORMIN HYDROCHLORIDE SITAGIIPTIN PHOSPHATE MONOHYDRATE", "METFORMIN BASICS", "METFORMIN LOVASTATIN DOCUSATE LIDOCAINE", "METFORMIN 850MG TID", "METFORMINE 1000MG", "DAPAGLIFLOZINA METFORMINA", "METFORMIN HYDROCHLORIDE ROSIGLITAZONE MALEATE", "GLUCOMINE METFORMIN HYDROCHLORIDE", "FUROSEMIDE LISINOPRIL METFORMIN", "METFORMIN HCL POW", "DONEPEZIL METFORMIN IBUPROFEN", "METFORMIN AN", "METFORMIN 1000 MG PO BID", "METFORMIN 500MG XR", "METFORMIN HCL GENERIC FOR GLUCOPHAGE", "METFORMIN TAB 500 MG ER", "METFORMINA AUROBINDO 1000 MG COMPRESSE RIVESTITE CON FILM", "METFORMINE HYDROCHLORIDE MYLAN 500 MG FILMOMHULDE TABLETTEN", "GLUCOVANCE GLIBENCLAMIDE METFORMIN", "VILDAGLIPTIN AND METFORMIN HYDROCHLORIDE", "METFORMINE ARROW 850 MG COMPRIME PELLICULE", "METFORMIN ATORVASATIN LEVOTHYROXINE", "METFORMIN 500 MG PO DAILY", "ZOMARIST MET METFORMIN HYDROCHLORIDE VILDAGLIPTIN", "METFORMIN ESTENDED RELEASE", "METFORMIN HCL 500MG TABLET", "METFORMIN ER CAP", "METFORMINE MYLAN 1000 MG COMPRIM S PELLICUL S", "METFORMIN SITAGLIPTINE", "METFORMIN 500MG TABLET TWIC A DAY", "LEVOTHYROXIN METFORMIN NYSTATIN", "ORAMET METFORMIN", "METFORMIN 500MG 2 TAB BID", "METFORMIN ARCANA", "METFORMIN MEDA 500 MG FILMDRAGERADE TABLETTER", "METFORMIN SANDOZ 500 MG TABLETTER FILMDRASJERTE", "METFORMIN HCL EXTENDED", "CHLORPROPAMIDE METFORMIN", "METFORMIN MYLAN 500 MG FILM COATED TABLETS", "GLIPIZIDEM METFORMIN", "METFORMINA MYLAN GENERICS", "METFORMINA CLORHIDRATO", "METFORMIN 1000MG 2XDAY", "METFORMIN HCL ER 750 MG", "METFORMIN 500MG 2 DAY", "GLYBURIDE METFORMIN 2 5 MG 500MG TAB", "METFOERTAB A ACTAVIS METFORMIN", "METFORMINE HCL 850 MGMETFORMINE TABLET 850MG", "METFORMIN THIOCTACID", "METFORMIN HEX", "METFORMIN HCL ER TABS", "METFORMIN HYDROCHLORIDE ALOGLIPTIN BENZOATE", "METFORMIN TEVA 1000 MG APVALKOTAS TABLETES", "GLIFOR GLIBENCLAMIDE METFORMIN HYDROCHLORIDE", "METFORMIN HYDROCHLORIDE TOLBUTAMIDE", "METFORMIN HEXAL 500 MG FILMTABLETTEN", "METFORMIN 1G 2 D", "ALOGLIPTIN WITH METFORMIN KAZANO", "METFORMINA DAPAGLIFLOZINA", "VIDAGLIPTIN METFORMIN", "METFORMIN DC", "VILDAGLIPTIN METFORMIN SANDOZ", "METFORMIN 100MG TWICE DAILY", "METFORMIN COMMON MOLECULE FROM UK MARKET", "METFORMIN HYDROCHLORIDE W TENELIGLIPTIN HYDRO", "GLUCONORM TAB UNCOA SR 1000 MG METFORMIN HYDROCHLORIDE", "METFORMIN 500MG TABS", "METFORMIN HYDROCHLORIDE EXTENDED RELEASE TABLET USP 500 MG", "METFORMINE HCL TEVA TABLET 500 MGMETFORMINE HCL TEVA TABLET 500MGM", "METFORMINA AWP", "LANTUS AND METFORMIN", "METFORMINE TABLET 850 MG MILLIGRAM", "METFORMIN GLYCON", "METFORMINA PFIZER", "MEFORAL METFORMIN HYDROCHLORIDE", "METFORMINE HCL MYLAN TABLET 850MG", "METFORMIN GABAPENTIN JARDIANCE ENTRESTO METOPROLOL SUCCINATE", "METFORMIN 1GM", "GLYBURIDINE METFORMIN", "LEVOTHYROXIN LISINOPRIL METFORMIN", "METFORMIN HYDROCHLORIDE ER TABLET", "GLUCONORM SR METFORMIN HYDROCHLORIDE", "METFORMIN HYDROCHLORIDE 250MG MT PFIZER", "GLYBURIDE METFORMIN 5 500MG 2 TABLETS BID", "GLIPIZIDE METFORMIN 5 500MG 2 TABLETS BID", "METFORMINE ARROW 1000 MG COMPRIME PELLICULE SECABLE", "METFORMIN HCL PIOGLITAZONE HCL", "METFORMINE EG", "METFORMIN 1000 MG TABLET", "METFORMIN TAB 500MG ER", "METFORMINSR 1000 MG", "METFORMIN CINFA", "METFORMINE EG 1000 MG COMPRIM PELLICUL S CABLE", "METFORMIN HYDROCHLORIDE EXTENDED RELEASE TABLETS USP 500 MG", "METFORMIN HYDROCHLORIDE E", "METFORATIO METFORMIN HYDROCHLORIDE", "METFORMIN HYDROCHLORIDE TEVA", "METFORMIN 2000MG PO DAILY AT BEDTIME", "METFORMIN HCL ER 500 MG PO LUNCH", "METFORMINA KERN PHARMA", "METFORMIN TABLETS 500 MG WHITE FRONT H 102", "METFORMIN HCL XR 500 MG", "METFORMIN HCL XR 750 MG", "METFORMIN 1G QPM", "METFORMIN SANDOZ 500 MG FILMDRAGERAD TABLETT", "METFORMIN ER 500MG DIGITEK", "METFORMINE KR METFORMIN HYDROCHLORIDE", "GLYBURIDE METFORMIN GLYBURIDE 5MG METFORMIN 500MG TAB", "METFORMIN TWICE A DAY", "METFORMIN HCL 100 POWDER", "METFORMIN XR 1500MG", "GLYCON METFORMIN HYDROCHLORIDE", "GLYBURIDE METFORMIN 2 5 500MG", "METFORMIN ER ACTAVIS", "METFORMIN ER GRANULES PHARMA", "DPP METFORMIN HYDROCHLORIDE", "METFORMIN HCL ER 500 MG TAB", "METFORMIN HYDROCHLORIDE EXTEND RELEASE", "GLIPIZIDE METFORMIN COMBINATION", "METFORMINA HEXAL", "METFORMIN HYDROCHLORIDE W SAXAGLIPTIN HYDROCH", "GLUCOVANCE GLIBENCLAMIDE METFORMIN HYDROCHLORIDE", "WELCHOL DIOVAN METFORMIN AMOXICILLIN CLAVANUATE SYNTHROID TOPROL", "METFORMIN METFORMIN HCL 500MG TAB", "METFORMINE MYLAN 1000 MG COMPRIM DISPERSIBLE", "METFORMIN HYDROCHLORIDE MT DSEP", "METFORMIN EXTENDED RELEASE TEVA", "METFORMIN METFORMIN HCL 500MG TABS SA", "METFORMIN 500 1 A PHARMA", "METFORMIN 1500MG DAILY", "SITAGLIPTIN METFORMIN COMBINED TABLET", "METFORMIN 50 ER", "METFORMIN TAB 500 MG", "METFORMIN HYDROCHLORIDE MITIGLINIDE CALCIUM HYDRATE", "METFORMINE ALMUS 850 MG COMPRIM PELLICUL", "METFORMIN AXAPHARM", "METFORMIN W GLIBENCLAMIDE", "METFORMIN DIOVAN", "ACTAVIS METFORMIN EXTENEDED RELEASE", "METFORMIN TIME RELEASE", "METFORMIN METFORMIN HCL 1000MG TAB", "METFORMIN GLUCOPHAGE 1000 MG TABLET", "METFORMIN 550MG", "LINAGLIPTINA METFORMINA", "GLICLAZIDE METFORMIN ROSIGLITAZONE", "POTASSSIUM METFORMIN", "METFORMIN HCL ORAL TABLET 1000MG", "EMPAGLIFLOZIN AND METFORMIN HYDROCHLORIDE", "METFORMIN ALMUS", "METFORMINE ARROW 500 MG COMPRIMEPELLICULE", "METFORMIN TENELIGLIPTIN", "METFORMIN 500 MG ORAL", "METFORMIN METFORMIN HCL 1000MG TAB SA", "METFORMIN HCL ER 500MG TABLETS", "METFORMIN HCL TAB 1000 ER", "METFORMIN WITH VILDAGLIPTIN", "METFORMIN 1000MG ORAL", "METFORMIN 1000MG EXTENDED RELEASE", "METFORMIN 500MG ORAL", "SAXAGLIPTIN AND METFORMIN", "ALOGLIPTIN BENZOATE METFORMIN HYDROCHLORIDE", "METFORMIN ER 1000MG TABLETS", "METFORMIN SITAGLIPTIN", "METFORMIN 100MG TABLETS", "METFORMIN HCL 1000MG TABLET", "DAPAGLIFLOZIN METFORMIN", "GALVUS MET METFORMIN HYDROCHLORIDE VILDAGLIPTIN", "JANUMET METFORMIN HYDROCHLORIDE SITAGLIPTIN", "METAPHAGE METFORMIN", "METFORMIN ER 500MG TWICE DAILY", "METFORMIN HYDROCHLORIDE EXTENDED RELEASE TABLET 500MG ACTAVIS", "METFORMINER OSMOTIC", "EMPAGLIFLOZIN METFORMIN HYDROCHLORIDE", "METFORMIN 850MG BID FOR DIABETES", "RIOMET METFORMIN HYDROCHLORIDE", "METFORMIN HCL 500 MG ER", "SITAGLIPTINE METFORMIN", "METFORMIN AND VILDAGLIPTIN", "METFORMIN ACTAVIS 850 MG FILMDRAGERAD TABLETT", "METFORMIN LINAGLIPTIN", "METFORMINA GLIBENCLAMIDA GLIBENCLAMIDE METFORMIN HYDROCHLORIDE", "METFORMIN 500MG QAM MEAL", "METFORMIN HYDROCHLORIDE EXTENDED RELEASE TABLETS USP 500MG", "METFORMIN EXT REL 500", "METFORMIN 100 MG PO DAILY", "METFORMIN HCL ER 500 MG TABLETS", "METFORMIN ER 500MG 24HR TABS", "METFORMINA CINFA 850 MG COMPRIMIDOS RECUBIERTOS CON PELICULA EFG 50 C", "METFORMIN ER 500 MG TABLET EXTENDED RELEASE 24 HR", "METFORMIN METFORMIN HCL 500MG TAB SA", "WANQUAN METFORMIN HYDROCHLORIDE", "METFORMIN HYDROCHLORIDE EXTENDED RELEASE USP 500 MG NDC65162 178 10", "METFORMIN ER 500MG 24 HR TABS", "METFORMIN ER 500MG TABLET GENERIC", "METFORMIN HYDROCHLORIDE EXTENDED RELEASE TABLETS USP 500MG", "AMNEAL METFORMIN ER 500 MG", "METFORMIN ER 500 MG TAB AMN", "METFORMIN HYDROCHLORIDE EXTENDED RELEASE TABLETS USP", "METFORMIN HYDROCHLORIDE MT JG", "ACARBOSE METFORMIN HYDROCHLORIDE", "METFORMINA MYLAN ITALIA 850 MG COMPRESSE RIVESTITE CON FILM", "METFORMIN HYDROCHLORIDE ER 500MG", "METFORMIN ER 500MG TAB AMN NDC 65162 178 10", "METFORMINA DAPAGLIFLOZINAV", "METFORMIN XR 500 MG 2 TABLETS 2 TIMES DAILY WITH MEALS", "METFORMIN HCI EXTENDED RELEASE 500 MG TABLETS", "METFORMIN HYDROCHLORIDE ER TEVA", "AURO METFORMIN 500 MG TABLETS", "METFORMIN HYDROCHLORIDE EXTENDED RELEASE TABLETS 750MG ND", "GLUCOMET GLIBENCLAMIDE METFORMIN HYDROCHLORIDE", "METFORMIN ECL ER 500 MG", "GLIPIZIDE AND METFORMIN HYDROCHLORIDE GLIPIZIDE METFORMIN HYDROCHLORI", "METFORMIN HCL ER TABS 750 MG", "METFORMIN 500MG EXTENDED RELEASE TABLETS", "GLUCOFIT METFORMIN HYDROCHLORIDE", "METFORMIN HCL 750 MG TABLET", "METFORMIN HCL TABS 500 MG GENERIC FOR GLUCOPHAGE TABS", "METFORMIN HCL ER 500MG TABLET", "GALVUS MET METFORMIN VILDAGLIPTIN", "METFORMIN HCL ER TABS 750MG", "CLORHIDRAT DE METFORMIN", "METFORMIN HYDROCHLORIDE PIOGLITAZONE HYDROCHLORIDE", "RECALL METFORMIN HYDROCHLORIDE EXTENDED RELEASE TABLETS USP 500MG", "METFORMIN HYDROCHLORIDE 500 MG", "GLYFORMIN METFORMIN", "METFORMIN 500 MG TWICE DAILY", "METFORMIN OG SITAGLIPTIN", "METFORMIN HCL TABS 500MG", "METFORMIN HCLXR 750MG", "SITAGLIPTIN PLUS METFORMIN HYDROCHLORIDE", "METFORMIN HCL AUROBINDO", "00082702001 METFORMIN HYDROCHLORIDE", "METFORMIN HYDROCHLORIDE SITAGLIPTIN", "METFORMINE NOS 850 MG", "AMN METFORMIN", "METFORMIN 1000 MG ORAL TWICE DAILY WITH MEALS", "METFORMIN HYDROCHLORIDE TAGI", "GLYBURIDE METFORMIN GLUCOVANCE 5 500", "GLIMEPIRIDE AND METFORMIN", "CALCIUM SINGULAIR FUROSEMIDE AMLODIPINE VITAMIN D METFORMIN", "METFORMINHCL ER", "METFORMIN ANDA 213651", "METFORMIN 500 MG 1BID", "AMARYL GLIMEPIRIDE METFORMIN HYDROCHLORIDE", "METFORMIN HYDROCHLORIDE SITAGL IPTIN PHOSPHATE MON", "PIOGLITAZONE METFORMIN 15 500MG", "METFORMIN 1 A PHARMA", "METFORMIN METFORMIN HCL 850MG TAB", "METFORMIN ER TIME CAP LABS", "GLIMEPIRIDE W METFORMIN HYDROCHLORIDE", "METFORMIN GENERIS 850 MG FILM COATED TABLETS", "KOMBIGLYZE XR METFORMIN SAXAGLIPTIN", "METFORMIN HYDROCHLORIDE SITAGL IPTIN PHOSPHATE", "METFORMIN LONG", "METFORMIN HCL AUROBINDO FILM COATED TABLETS 850MG", "YUHAN METFORMIN XR", "METFORMINE TABLET 500MG BRAND NAME NOT SPECIFIEDMETFORMINE TABL", "METFORMIN MYLAN 850 MG FILM COATED TABLETS", "LOFIN METFORMIN HYDROCHLORIDE", "GLIMEPIRIDE AND METFORMIN HYDROCHLORIDE", "METFORMIN HYDROCHLORIDE NOT EXTENDED RELEASE NON COMPANY PRODUCT", "METFORMIN 750MG ER TABLETS", "METFORMIN HYDROCHLORIDE ANDA 213651", "METFORMIN 1500 MG BID", "METFORMIN BLUEFISH METFORMIN", "METFORMIN ER 750", "EMPAGLIFLOZIN METFORMIN EMPAGLIFLOZIN 12 5MG METFORMIN 1000MG 24HR", "METFORMIN HYDROCHLORIDE GRANULES PHARMACEUTICALS", "METFORMINE TABLET 500MG BRAND NAME NOT SPECIFIED", "METFORMIN HYDROCHLORIDE 850 MG FILM COATED TABLETS", "GLIMEPIRIDE METFORMIN ULTRA", "METFORMIN HCL ER 750MG", "GLYCON METFORMIN", "METFORMIN METFORMIN HCL 100MG TAB", "METFORMIN FILM COATED TABLET 500 MG", "METFORMINE SANDOZ 500 MG FILMOMHULDE TABLETTEN", "METFORMIN 500 MG IMMEDIATE RELEASE", "METFORMINE TABLET MGA 500MG BRAND NAME NOT SPECIFIED", "SITAGLIPTIN PHOSPHATE MONOHYDRATE METFORMIN HYDROCHLORIDE", "METFORMIN HYDROCHLORIDE ACTAVIS", "METFORMINE BLUEFISH", "METFORMINE EG 500 MG COMPRIME PELLICULE", "METFORMIN HYDROCHLORIDE EXTENDED RELEASE 500 MG TABLETS USP", "METFORMIN HCL ER MOD 1000 MG ER TABLETS", "GLUCAMINOL METFORMIN", "METFORMINE TABLET 500MG BRAND NAME NOT SPECIFIED", "METFORMIN 1A", "METFORMIN HCL 500 MG TAB 180 METFORMIN HYDROCHLORIDE", "METFORMIN CHINOIN", "METFORMIN 850 1 A PHARMA", "METFORMIN 500 MG ORAL TABLET ER", "METFORMIN HCL ZYDUS MANUFACTURER", "METFORMIN HYDROCHLORIDE EXTENDED RELEASE TABLET USP 500MG", "AURO METFORMIN 850 MG TABLETS", "METFORMIN HCL ER 750MG TB24", "METFORMIN ATORVASTATIN", "METFORMIN 500MG DAILY", "METFORMINE GLIBENCLAMIDE TABLET FO 500 5MG BRAND NAME NOT SPECIFIED", "METFORMIN HYDROCHLORIDE EXTENDED RELEASE TABLETS 500 MG", "METFORMIN EXTENDED RELEASE GASTRIC", "METFORMIN NICHIIKO", "METFORMIN HCL ER 750 MG TABLET", "METFORMIN HCL 500MG 24 HR SA TAKE 4 PILLS DAILY", "METFORMIN RATIOPHARM 500 MG APVALKOTAS TABLETES", "GLUCAMIN METFORMIN HYDROCHLORIDE", "SIBET METFORMIN HYDROCHLORIDE", "METFORMINE TEVA 850 MG COMPRIME PELLICULE", "LINAGLIPTIN METFORMIN 2 5MG 1000MG 2 TABS DAILY", "METFROMIN METFORMIN HCL 850MG TAB", "METTFORMIN METFORMIN HCL 500MG TAB", "METFORMIN METFORMIN HCL 500MG TAB SA", "METFORMIN 1000MG FTA", "GLIMEPRIDE METFORMIN HYDROCHLORIDE", "DBI METFORMINA", "METFORMIN HCL 500MG ORAL", "METFORMIN ORAL", "METFORMIN HYDROCHLORIDE W SITAGLIPTIN PHOSPHA", "METFORMIN 599", "LINAGLIPTIN METFORMIN 5MG 1000MG DAILY", "METFORMIN HCL 1000MG ORAL", "METFORMIN HCL XR 750MG TAB", "DAPAGLIFOZIN METFORMIN", "METFROMINMETFORMIN", "METFORMIN ERT", "METFORMIN STRENGTH", "METFORMIN HCL ER 1000MG ORAL", "METFORMINE TABLET 850MG BRAND NAME NOT SPECIFIED", "METFORMIN HRDROCHLORIDE TABLETS 500MG", "METFORMIN 500 MG DAILY", "METFORMIN 500 MG ER TAB PAR", "METFORMINE TABLET 850MG BRAND NAME NOT SPECIFIEDMETFORMINE TABL", "NAPROXEN TIZANIDINE MONTELUKAST METFORMIN LISINOPRIL NUCYNTA", "ALBUTEROL PREGABALIN METFORMIN NEXLIZET BUSPIRONE", "METFORMINE SUN", "METFORMIN 100 MG TWICE DAILY", "METFORMINE HYDROCHLORIDE", "GLYBURIDE AND METFORMIN HYDROCHLORIDE TABLETS 5 500 MG", "METFORMIN 1000 MG TAB", "GLUCONORM TAB UNCOA ER 500 MG METFORMIN HYDROCHLORIDE", "METFORMIN 100", "METFORMIN HCL ER 750MG TABLET", "CLONIDINE OZEMPIC METFORMIN", "METFORMIN 1000MG PO BID", "DAPAGLIFLOZIN METFORMIN HCL ER 5 1000MG", "METFORMINE ARROW LAB 500 MG COMPRIME PELLICULE", "METFORMIN 500 MG BID", "METFORMIN KERN PHARMA", "METFORMIN HYDRCOCHLORIDE TABLETS USP RX ONLY", "YUAN TANG METFORMIN HYDROCHLORIDE", "EMPAGLIFLOZIN METFORMIN EMPAGLIFLOZIN 5MG METFORMIN 1000MG 24 HR TAB", "METFORMIN GASTRIC", "METFORMINE TABLET MGA 1000MG BRAND NAME NOT SPECIFIED", "METFORMIN 1000 1 A PHARMA", "METFORMIN GLIPIZDE", "METFORMIN HYDROCHLORIDE EXTENDED RELEASE TABLETS 750 MG", "METFORMIN EMPAGLIFLOZIN", "GLUCOMET METFORMIN", "METFORMIN HCI 750 MG", "TEVA METFORMIN HCL", "GLIMET GLIMEPIRIDE METFORMIN HYDROCHLORIDE", "METFORMIN HEXAL 500 MG FILMTABLETTEN", "GLIPZIDE METFORMIN", "METFORMIN HCL ER TABLET EXTENDED RELEASE 24 HOUR 500 MG", "METFORMIN VOGLIBOSE", "METFORMIN ROSUVASTATIN", "METFORMIN HYDROCHLORIDE ALOGLIPTIN", "METFORMIN ER 500MG SUBSTITUTED FOR GLUCOPHAGE XR500MG", "METFORMIN 1 000 MG TWICE DAILY", "METFORMIN 100MG BID", "GLIPIZIDE METFORMIN 5MG 500MG", "METFORMIN ARROW 850 MG FILM COATED TABLETS", "METFORMIN ER 750 MG DAILY", "METFORMIN HEXAL 500 MG", "METFORMINUM METFORMIN", "METFORMINE HYDROCHLORIDE BRAND NAME NOT SPECIFIED", "METFORMINA AUROVITAS", "METFORMINA AUROVITAS 850 MG COMPRIMIDOS RECUBIERTOS CON PELICULA", "METFORMINE TABLET 500MG BRAND NAME NOT SPECIFIEDMETFORMINE TABL", "METFORMIN HYDROCHLORIDE TAB 250MG MT TE", "APO METFORMIN XR", "METFORMINXR", "GALVUMET METFORMIN HYDROCHLORIDE VILDAGLIPTIN", "GLIPIZIDE AND METFORMIN HYDROCHLORIDE GLIBENCLAMIDE METFORMIN HYDROCHLORIDE", "LISINOPRIL METFORMIN METHOTREXATE", "METFORMIN 500MG DAILY WITH DINNER", "EMPAGLIFLOZIN LINAGLIPTIN METFORMIN", "METFORMIN HCL ER TABS 500MG GENERIC FOR GLUCOPHAGE XR TABS", "METFORMIN LISINOPRIL SIMVASTATIN LANTUS", "DAPAGLIFLOZIN METFORMIN XIGDUO", "METFORMIN HERITAGE", "METFORMIN HCL 1000MG", "METFORMIN HYDROCHLORIDE AND GLIPIZIDE", "METFORMIN 100 MG TAB", "METFORMINE 850MG", "METFORMIN 1000MG 2 DAY", "YUHAN METFORMIN XR TAB", "METFORMIN HCL ORAL TABLET 500 MG", "METFORMIN HEXAL 850 MG FILMTABLETTEN", "KOMBIGLYZE XR METFORMIN HYDROCHLORIDE SAXAGLIPTIN HYDROCHLORIDE", "METFORMIN 200 MG", "MF METFORMIN HYDROCHLORIDE", "METFORMIN AXAPHARMA", "METFORMIN 500 MG TAB", "METFORMIN NORCO", "METFORMINA CINFA 850 MG COMPRIMIDOS RECUBIERTOS CON PELICULA EFG", "GLUCOPHAGE COMPARATOR METFORMIN", "REVATIO METFORMIN", "METFORMINA TEVA 850 MG COMPRIMIDOS RECUBIERTOS CON PELICULA EFG", "EMPAGLIFLOZIN METFORMIN EMPAGLIFLOZIN 5MG METFORMIN 1000MG TAB ORAL", "OMEPRAZOLE AMLODIPINE LATANOPROST BRIMONIDINE METFORMIN", "METFORMINN 1000MG BID", "METAMUCIL METFORMIN HYDROCHLORIDE", "METFORMIN SANDOZ", "METFORMIN METFORMIN HCL 500MG 24HR TAB SA", "ALOGLIPTAN METFORMIN", "METFORMIN XR 500MG TABLETS", "METFORMIN HCL XR 500MG TABLET", "ERTUGLIFLOZIN PIDOLATE METFORMIN HYDROCHLORIDE", "500 MG METFORMIN HYDROCHLORIDE", "METFORMIN ER GSTRIC", "METFORMINA XR", "METFORMINE HCL CF", "METFORMIN HCL ER 500MG COMMON BRAND SEE DRUG MONOGRAPH", "EMPAGLIFLOZIN METFORMIN EMPAGLIFLOZIN 12 5MG METFORMIN 1000MG TAB O", "METFORMIN R A N", "DAPAGLIFLOZIN METFORMIN XR 5 1000MG", "METFORMINA SANDOZ 850 MG COMPRIMIDOS REVESTIDOS", "METFORMIN AUROBINDO FILMCOATED TABLETS 1000MG", "METFORMINE TABLET 1000MG BRAND NAME NOT SPECIFIED", "METFORMIN AUROBINDO FILMTABLETTEN 500 MG", "SEMAGLUTIDE METFORMIN", "METFORMINE TABLET 1000MG BRAND NAME NOT SPECIFIEDMETFORMINE TABL", "METFORMIN AUROBINDO FILMTABLETTEN 1000MG", "METFORMIN BDC", "METFORMIN HCL XR 500 MG TABLET IMPRINT G7", "METFORMIN HYDROCHOLORIDE", "VILDAGLIPTIN METFORMIN HYDROCHLORIDE", "METFORMIN GLUCOPHAGE 1 000 MG TABLET", "PMS METFORMIN TAB 500 MG", "METFORMIN 500 MG PO", "METFORMIN 1000 MG QD", "GLIPIZIDE AND METFORMIN", "CRISOMET METFORMIN HYDROCHLORIDE SITAGLIPTIN PHOSPHATE", "SITAGLIPTIN METFORMIN JANUMET 50 1000 MG TAKE TWO TABLETS PO QD", "KOMBIGLYZE XR METFORMIN HYDROCHLORIDE SAXAGL", "METFORMIN 500MG PO DAILY", "METFORMINE MGA BRAND NAME NOT SPECIFIED", "PIOGLITAZONE HYDROCHLORIDE AND METFORMIN HYDR", "METFORMINA SANDOZ 850 MG COMPRIMIDOS RECUBIERTOS CON PELICULA EFG", "METFORMIN ATID 500 MG", "METFORMIN FOR DIABETES", "AD METFORMIN", "PIOGLITAZONE CLORIDRATO METFORMINA CLORIDRATO", "METFORMIN 1000 1A PHARMA", "KOMBIGLYZE XR METFORMIN HYDROCHLORIDE SAXAGLIPTIN MONOHYDRATE", "SUGAMET EVOGLIPTIN TARTRATE METFORMIN HYDROCHLORIDE", "METFORMIN 1000 MG TWICE DAILY HOME MED", "METFORMIN ER 500MG AB1", "METFORMINE TABLET MGA 500MG BRAND NAME NOT SPECIFIEDMETFORMINE T", "METFORMIN TAB 850MG", "METFORMIN 500 1A PHARMA", "EMPAGLIFLOZIN METFORMIN", "GLIPIZIDE METFORMIN 5 500MG", "MECLIZINE 25MG RX TABLETS METFORMIN 500MG TABLETS NAMENDA 10MG TABLETS", "METFORMIN SITAGLIPTIN PHOSPHATE", "ALOGLIPTIN AND METFORMIN", "METFORMIN REPAGLINIDE", "METFORMINE METFORMINE HCL", "METFORMIN 750MG", "METFORMIN GRANULES", "METFORMIN 1000MG DAILY", "METFORMIN ACCORD 500 MG FILMDRAGERAD TABLETT", "EMPAGLIFLOZIN LINAGLIPTIN METFORMIN HYDROCHLORIDE", "METFORMIN 500 MG TABLET BID W MEALS", "METFORMMETFORMININ", "METFORMIN XR 750 MG", "METFORMIN 500MG 4 PER DAY", "METFORMIN GLUCOPHAGE 1000MG TABLET", "METFORMIN HCL TAB", "METFORMIN XL PCOS", "METFORMIN 1000 X 2", "METFORMIN MEDICAL VALLEY", "METFORMIN SITAGLIPIN", "METFORMIN 850MG TABLET", "XIGDUO DAPAGLIFLOZIN METFORMINHYDROCHLORID", "RELONCHEM METFORMIN", "VILDAGLIPTIN METFORMIN TEVA", "MET METFORMIN HYDROCHLORIDE", "ALOGLIPTIN AND METFORMIN HYDROCHLORIDE", "METFORMIN XR 500 MG 2X DAY", "METFORMIN GA", "SITGLIPTIN METFORMIN 50 1000", "NEO METFORMIN METFORMIN HYDROCHLORIDE", "GEMIGLIPTIN METFORMIN", "SITAGLIPTIN METFORMIN 50 1000", "EMNORM METFORMIN", "METFORMINA ACCORD", "ATORVASTATIN METFORMIN", "METFORMIN MYLAN ITALY", "METFORMIN CT", "METFORMIN HCL KOREAUNITED", "METROMIN METFORMIN HYDROCHLORIDE", "VICTOZA 1 8 METFORMIN HCI 24HR 500 MG", "METFORMINE TABLET MGA 500MG GLUCIENT SR TABLET MVA 500MGGLUCIENT", "TABLET METFORMIN", "1327063 GLOBALC3SEP21 METFORMIN HCL", "DAPAGLIFLOZIN METFORMIN HYDROCHLORIDE", "ARB METFORMIN", "METFORMIN PILL", "METFORMIN XR 1000MG DAILY", "METFORMIN ACCORD", "METFORMIN POW HCL", "METFORMIN ER 500MG 1XD PM", "METFORMIN XR 2000 MG PO DAILY", "METFORMIN HYDROCHLORIDE MITIGLINIDE CALCIUM", "METFORMIN 1000 MG CANAGLIFLOZIN 150 MG", "VILDAGLIPTIN METFORMIN TEVA", "METFORMIN LICH 1000 MG FILM COATED TABLETS", "METFORMIN TEMIS", "MEGLUMINE METFORMIN HYDROCHLORIDE", "METFORMIN TRAZADONE", "METFORMIN 500MG SA TAB", "METFORMIN CANAGLIFLOZIN", "METFORMINE ALMUS 850 MG COMPRIME PELLICULE", "METFORMIN TAB 500MG METFORMIN TAB 500MG ER", "METFORMIN 500 TWICE DAILY", "CANAGLIFLOZIN HEMIHYDRATE METFORMIN HYDROCHLORIDE", "ANAGLIPTIN METFORMIN HYDROCHLORIDE", "GLINEXT METFORMIN HYDROCHLORIDE", "METFORMIN KARO PHARMA", "METFORMIN HCL ORAL TABLET", "METFORMIN 1000MG PO BID", "METFORMIN EXTENDED RELEASE ER", "SAXAGLIPTIN METFORMIN ER", "METFORMINE EMBONATE DE", "METFORMIN XR 750MG TABLET", "METFORMINA EG", "GLYCOMET METFORMIN", "METFORMIN HTCZ", "METFORMIN HYDROCHLORIDE TABLETS GLUCOPHAGE", "METFORMIN HEXAL 1G", "MET METFORMIN", "METFORMIN 1000MG TWICE A DAY", "LEVOTHYROXINE METFORMIN", "DAPAGLIFLOZIN METFORMIN HYDROCHLORIDE SAXAGLIPTIN HYDROCHLORIDE", "METFORMIN ER FOR PRE DIABETES", "METFORMIN HCI BASAGLAR KWIKPEN", "ALOGITIPIN METFORMIN", "METFORMIN HCL ORAL TABLET 1000 MG", "METFORMIN 750MG XR ORAL TABLET", "METFORMIN 1000MG TABLETS 1BID", "500 MG METFORMIN HCL METFORMIN HYDROCHLORIDE", "METFORMIN ER GASTRIC 500 MG TABERGR24H", "METFORMIN JANUMET XR", "SITAGLIPTIN METFORMIN JANUMET", "METFORMIN HCL 1000 MG TWICE DAILY", "METFORMIN ABZ 1000 MG FILMTABLETTEN", "METFORMIN HC TB2 750MG", "METFORMINUM SIOFOR", "LINAGLIPTIN METFORMIN", "METFORMIN COMBO", "METFORMIN HCL ER ORAL TABLET EXTEND 500 MG", "500 MG METFORMIN HCL", "METFORMIN MEFORAL", "METFORMIN TAB 750MG ER", "METFORMIN METFORMTN", "SUGAMET METFORMIN HYDROCHLORIDE", "CLOTRIMAZOLE GLIPIZIDE METFORMIN", "METFORMINA AUROVITAS SPAIN", "DONGSUNG METFORMIN HCL", "METFORMIN HC TAB 1000MG", "METFORMIN HCI DONGSUNG", "METFORMIN SUSTAINED RELEASE TABLETS", "SYNJARDY EMPAGLIFLOZIN METFORMIN HYDROCHLORIDE", "METFORMIN HC 1000MG", "ELIQUIS METFORMIN", "METFORMIN HC TAB 500MG", "GLYBURIDE METFORMIN 5 500 MG 2 TABLETS TWO TIMES DAILY", "METFORMIN ER 1000 MG DAILY", "METFORMIN HCL TAB 1000 MG", "1000 MG METFORMIN HCL", "DAPAGLIFLOZIN METFORMIN TABX 5 1000 MG", "METFOGAMMA METFORMIN HYDROCHLORIDE 1000 MG", "SITAGLIPTIN METFORMIN JANUMET XR", "METFORMINA STADA", "METFORMIN HC TB2 500MG", "METFORMIN HCI 500MG", "METFORMIN HCL 1000 MG", "DAPAGLIFLOZIN METFORMIN HCI", "METFORMINE 850 TABLETS", "METFORMINE TABLET MGA 500MG GLUCIENT SR TABLET MVA 500MG", "METFORMINE ALTER 500 MG COMPRIME PELLICULE", "METFORMIN H TAB 1000MG", "VILDAGLIPTIN GLYBURIDE METFORMIN", "METFORMIN 500 MG 1X DAILY", "CLORHIDRATO DE METFORMINA", "METFORMIN TAB 1000MG", "800MG METFORMIN", "METFORMIN HYDROCHLORIDE PIOGLITAZONE", "ACARBOSE METFORMIN", "METFORMIN HYDROCHLORIDE ENTERIC COATED TABLETS", "GLIPIZIDE METFORMIN HYDRO", "METFORMINA CINFA 850 MG COMPRIMIDOS RECUBIERTOS CON PELICULA", "METFORMIN HYDROCHLORIDE SUSTAINED RELEASE TABLETS II", "METFORMIN MOUNJARO", "METFORMIN 1500 MG AM AND 1000 MG PM", "METFORMIN ER 500 MG TABLET EXTENDED RELEASE", "METFORMINA SITAGLIPTINA", "METFORMIN HYDROCHLORIDE EXTENDED RELEASE 500MG", "METFORMIN EQL", "ERTUGLIFLOZIN METFORMIN HYDROCHLORIDE", "METFORMIN HCL EXTENDED RELEASE 500 MG TABLET 2 DAY", "GLUCOPHAGE XR METFORMIN XR", "METFORMIN GLIFOR", "METFORMIN XR 1000MG BID", "METFORMIN HC TAB 500MG", "ALOGLIPTIN METFORMIN VIPDOMET", "METFORMIN JANUVIA", "METFORMINE ALMUS 850 MG FILM COATED TABLET", "METFORMINE CHLORHYDRATE VILDAGLIPTINE", "METFORMIN 500MG TWICE DAILY", "METFORMIN M R", "LINAGLIPTIN METFORMIN JENTADUETO", "METFORMIN LICH 500 MG FTA", "METFORMIN ER 500MG 1X DAY", "METFORMINA GLIBENCLAMIDA GLIBENCLAMIDE METFORMIN", "METFORMIN TABLET 850MG BRAND NAME NOT SPECIFIED", "DESMOPRESSIN CYMBALTA METFORMIN", "METFORMIN ER 500 MG TABLET", "METFORMIN HYDROCHLORIDE SUSTAINED RELEASC TABLETS", "METFORMINA MILLET", "METFORMIN SOL", "EMPAGLIFLOZIN METFORMIN SYNJARDY", "METFORMIN LINAGLIPTIN", "INVOKAMET CANAGLIFLOZIN METFORMIN HYDROCHLORIDE TABLET", "METFORMIN HC TAB", "GLYCOMET METFORMIN HYDROCHLORIDE", "METFORMIN 850 MG 2X1 TABLET DAILY", "METFORMIN HEXAL 850 MG FILMTABLETS", "METFORMIN HYDROCHLORIDE CAPSULES", "METFORMIN HCI ER MOD", "METFORMIN HYDROCHLORIDE AND GLICLAZIDE", "METFORMINA 1 000 MG COMPRIMIDO", "METFORMINA NORMON METFORMIN HYDROCHLORIDE", "PIOGLITAZONE METFORMIN GLIFIX PLUS", "GLIFORMIN METFORMIN HYDROCHLORIDE", "METFORMIN 750 MG ONCE DAILY", "METFORMIN XR 500 MG 2000 MG DAIL", "METFORMINE ALMUS 1000 MG COMPRIM PELLICUL", "EMPAGLIFLOZINE METFORMIN", "EVOGLIPTIN METFORMIN", "XIGDUO DAPAGLIFLOZIN METFORMIN HYDROCHLORIDE", "GLIFAGE XR 750 MG METFORMIN HYDROCHLORIDE", "SITAGLIPTINE METFORMINE", "METFORMIN 1 000 MG PO QD", "METFORMIN GLUCOPHAGE XR", "METFORMINACARBOSE", "JANUMET METFORMIN HYDROCHLORIDE SITAGLIPTIN PHOSPH", "ATORVASTATIN CALCIUM GLIMEPIRIDE METFORMIN HYDROCHLORIDE RAMIPRIL", "METFORMIN NATEGLINIDE", "METFORMIN HYDROCHLORIDE REPAGLINIDE", "GLIMEPIRIDE METFORMIN VOGLIBOSE", "GLIMEPIRIDE METFORMIN PIOGLITAZONE", "METFORMIN HYDROCHLORIDE TYROSINE", "Dimethylbiguanidine", "Dimethylguanylguanidine", "GLUCOPHAGE", "GLUCOPHAGE UNS", "GLUCOPHAGE ABIC", "GLUCOPHAGE XR", "GLUCOPHAGE METFORMN HYDROCHLORIDE", "GLUCOPHAGE XR METFORIN HYDROCHLORIDE", "GLUCOPHAGE HOECHST", "GLUCOPHAGE ARON", "GLUCOPHAGE X", "GLUCOPHAGE XL", "GENERIC GLUCOPHAGE 500 MG", "GLUCOPHAGE LIPHA", "GLUCOPHAGE 850 MILLIGRAM", "GLUCOPHAGE AB1C", "GLUCOPHAGE THERAPY UNSPECIFIED", "GLUCOPHAGE UNS", "GLUCOPHAGE UNKNOWN", "GLUCOPHAGE METROMIN HYDROCHLORIDE", "GLUCOPHAGE GLYBURIDE", "GLUCOPHAGE METFORMIM", "GLUCOPHAGE 500 MG TID", "GLUCOPHAGE METOFIMIN HYDROCHLORIDE", "METOFORMIN HYDROCHLORIDE GLUCOPHAGE SLOW RELEASE", "GLUCOPHAGE R", "GLUCOPHAGE UNKNOWN", "GLUCOPHAGE TABLETS 800 MG", "GLUCOPHAGE BRISTOLE MYERS SQUIBB", "GLUCOPHAGE BRISTOLMYER SQUIBB", "GLUCOPHAGE BRISTOLMYERS SQIBB", "GLUCOPHAGE METFROMIN HYDROCHLORIDE0", "GLUCOPHAGE MORTFORMIN HYDROCYHLORIDE", "GLUCOPHAGE BRISTOL MYERS SQUIBE", "LASIX GLUCOPHAGE", "GLUCOPHAGE METFORIM HYDROCHLORIDE", "GLUCOPHAGE 00082701", "GLUCOPHAGE BRISTOL MYEARS SQUIBB", "GLUCOPHAGE BRISTORL MYERS SQUIBB", "GLUCOPHAGE BRISTOR MYERS SQUIBB", "GLUCOPHAGE SLOW", "GLUCOPHAGE BRISTOL MYERS SQIBB", "GLUCOPHAGE BRISTOL SQUIBB", "GLUCOPHAGE BRISTOL MYERSE SQUIBB", "GLUCOPHAGE BRISTOL", "GLUCOPHAGE APIC", "GLUCOPHAGE USA", "GLUCOPHAGE BRISTOL MYEARS SQUIBB", "GLUCOPHAGE ER", "GLUCOPHAGE 00082702", "GLUCOPHAGE BRISTOL MYERS SQUIB", "GLUCOPHAGE BRISTOR MYERS SQUIBB", "GLUCOPHAGE 00082701", "AVANDAMET GLUCOPHAGE", "GLUCOPHAGE BRISOL MYERS SQUIBB", "GLUCOPHAGE BRISTOL BYERS SQUIBB", "GLUCOPHAGE BRISTROL MYERS SQUIBB", "GLUCOPHAGE BRISTOL MYERS SQIUBB", "GLUCOPHAGE 00082701", "ACTOS USA GLUCOPHAGE BRISTOL MYERS SQUIBB", "GLYBURIDE GLUCOPHAGE", "GLUCOPHAGE BRISTOL MYYERS SQUIBB", "GLUCOPHAGE BRITSOL MYERS SQUIBB", "GLUCOPHAGE SLOW RELEASE", "GLUCOPHAGE BRISTOL MYERS SQUBB", "GLUCOPHAGE GLUCOPHAGE", "GLUCOPHAGE SLOW RELEASE", "GLUCOPHAGE GLYBURIDE", "GLUCOPHAGE BRITOL MYERS SQUIBB", "ACTOS GLUCOPHAGE", "LANTUS GLUCOPHAGE BRISTOL MYERS SQUIBB", "GLUCOPHAGE ACTOS", "GLUCOPHAGE BRISTOL MYERS SQUIBB KEFLEX", "GLUCOPHAGE BRISTO MYERS SQUIBB", "GLUCOPHAGE PLUS ACTOS", "GLYBURIDE GLUCOPHAGE", "GLUCOPHAGE BRISTOL MYERS SQUIIBB", "INSULIN GLUCOPHAGE", "GLUCOPHAGE 00082701 MEFORMIN", "GLUCOPHAGE JNJ16269110 COMPARATOR TABLET", "GLUCOPHAGE METFOMRIN HYDROCHLORIDE", "GLUCOPHAGE BRISTOL MYERS SUIBB", "GLUCOPHAGE METMORMIN", "GLUCOPHAGE METFORMN", "GLUCOPHAGE MEFORMIN", "GLUCOPHAGE RETARD", "GLUCOPHAGE MTFORMIN", "GLUCOPHAGE 0082701 CON", "GLUCOPHAGE 00082701 MFR NOT SPECIFIED", "GLUCOPHAGE EXENATIDE PEN DISPOSABLE DEVICE EXENATIDE PEN", "GLUCOPHAGE BRISTOL MYERS SQUIBB PILL EXCEPT TABLETS", "GLUCOPHAGE USA METFORM HYDROCHLORIDE", "GLUCOPHAGE 0082702", "AM GLUCOPHAGE", "GLUCOPHAGE 500 MILLIGRAM TABLETS", "GLUCOPHAGE CAPSULES", "GLUCOPHAGE METPORMIN", "GLUCOPHAGE 000082701", "GLUCOPHAGE 0082701", "GLUCOPHAGE 500 MILLIGRAM", "GLUCOPHAGE SR", "GLUCOPHAGE CON", "GLUCOPHAGE AMARYL", "GLUCOPHAGE EQ", "JANUMET GLUCOPHAGE", "GLUCOPHAGE XR 500 MILLIGRAM TABLETS", "GLUCOPHAGE METFORMXN", "GLUCOPHAGE MFR NOT SPECIFIED", "GLUCOPHAGE FORTE", "GLUCOPHAGE USA", "GLUCOPHAGE XR TABS", "GLUCOPHAGE TABS 1000 MG", "GLUCOPHAGE TABS", "GLUCOPHAGE TABS 850 MG", "GLUCOPHAGE TABS 500 MG", "GLUCOPHAGE MEFORMIN HYDROCHLORIDE", "GLUCOPHAGE 1000 MG TABLET", "GLUCOPHAGE LA", "GLUCOPHAGE SCH", "GLUCOPHAGE MERCK", "GLUCOPHAGE GENERIC", "GLUCOPHAGE 00082701", "GLUCOPHAGE XR TABS 500 MG", "GLUCOPHAGE S", "GLUCOPHAGE 500 MG", "GLUCOPHAGE 500 MG BAGS", "GLUCOPHAGE CAPSULES", "GLUCOPHAGE POWDER", "GLUCOPHAGE 850 MG", "GLUCOPHAGE XR TABS 750 MG", "GLUCOPHAGE BRISTOL MYERS SQUIBB", "GLUCOPHAGE NON AZ PRODUCT", "GLUCOPHAGE F", "GLUCOPHAGE 1G", "GLUCOPHAGE GLUCOPHAGE", "GLUCOPHAGE CORIANDER OIL", "GLUCOPHAGE BRISTOL MYERS SQUIBB", "GLUCOPHAGE 850", "GLUCOPHAGE ACE INHIBITOR 500MG", "METFORIN HCL GLUCOPHAGE", "GLUCOPHAGE 850 MG COMPRESSE RIVESTITE BRUNO FARMACEUTICI S P A", "GLUCOPHAGE 500", "GENERIC GLUCOPHAGE", "GLUCOPHAGE LONG", "GLUCOPHAGE 1000 MG", "GLUCOPHAGE 1000", "GLUCOPHAGE GENERIC", "GLUCOPHAGE 500 MG COMPRESSE RIVESTITE", "GLUCOPHAGE 500 MG FILMTABLETTEN", "GLUCOPHAGE 1000 MG COMPRESSE RIVESTITE CON FILM", "GLUCOPHAGE 850 MG COMPRESSE RIVESTITE CON FILM", "GLUCOPHAGE MR", "GLUCOPHAGE 850 MG COMPRIME PELLICULE", "GLUCOPHAGE XR 1000MG BID", "EMLA GLUCOPHAGE", "GLUCOPHAGE 500MG", "GLUCOPHAGE 1000 MG COMPRIME PELLICULE", "GLUCOPHAGE 850 MG COMPRIM PELLICUL", "GLUCOPHAGE 1000 MG COMPRIME PELLICULE SECABLE", "GLUCOPHAGE 1000 MG COMPRIM PELLICUL", "GLUCOPHAGE 1000 MG COMPRIM PELLICUL S CABLE", "GLUCOPHAGE 500 MG COMPRESSE RIVESTITE CON FILM", "GENERIC GLUCOPHAGE XR", "GLUCOPHAGE RET", "GLUCOPHAGE 500 MG COMPRIM PELLICUL", "GLUCOPHAGE 500 MG COMPRIME PELLICULE", "GLUCOPHAGE 1000 MG BID", "DIAZEPAM GLUCOPHAGE", "GLUCOPHAGE XR TABLET", "GLUCOPHAGE XR 2000MG PO QDAY", "GLUCOPHAGE XR 1000MG", "GLUCOPHAGE XR 24 HR", "GLUCOPHAGE XR", "GLUCOPHAGE 1000MG", "GLUCOPHAGE 850MG", "GLUCOPHAGE 2000MG", "GLUCOPHAGE TAB", "GLUCOPHAGE 1000 MG FILM COATED", "GLUCOPHAGE WATER PILL", "GLUCOPHAGE 1000 MG COATED TABLET", "GLUCOPHAGE 1 000 MG TABLET", "METAFORMIN GLUCOPHAGE", "Hydrochloride, Metformin", "HCl, Metformin"
